# Supplementary material for: Mechanochemistry Frees Thiourea Dioxide (TDO) from the ‘Veils’ of Solvent, Exposing All Its Reactivity
Source: Molecules. 2023 Feb 28;28(5):2239. doi: 10.3390/molecules28052239 (PMC10005452; doi:10.3390/molecules28052239)
Supplement: Supplementary file 1 [file molecules-28-02239-s001.zip › molecules-2223116-supplementary.pdf]

SUPPORTING INFORMATION

Mechanochemistry Frees Thiourea Dioxide (TDO) from the 'Veils' of Solvent, Exposing All Its Reactivity

Francesco Basoccu [1], Federico Cuccu [1], Pietro Caboni [1], Lidia De Luca [2] and Andrea Porcheddu [1,*]

[1] Department of Chemical and Geological Sciences, University of Cagliari, 09042 Monserrato, Italy
[2] Department of Chemical, Physical, Mathematical, and Natural Sciences, University of Sassari, Via Vienna 2, 07100 Sassari, Italy
* Correspondence: porcheddu@unica.it

Number of pages: 48

Number of figures: 2

Number of schemes: 2

Number of tables: -

Table of Content

1. General information 2

2. Mechanochemical synthesis of **3a-j, 3o-p, 3s and 4d-n, 4o, 4q-r** 3

3. Green Metrics calculations 13

4. Spectra 19

5. References 47

1. General information

Commercially available reagents were purchased from Acros, Aldrich, Strem Chemicals, Alfa-Aesar, TCI Europe, and used as received. All reactions were monitored by thin-layer chromatography (TLC) performed on glass-backed silica gel 60 F254, 0.2 mm plates (Merck), and compounds were visualized under UV light (254 nm) or using cerium ammonium molybdate solution with subsequent heating. The eluents were technical grade. Mechanochemical reactions were carried out using a Retsch MM500 Vario apparatus. The reagents were milled using a stainless-steel grinding jar (10 mL) equipped with two balls (ϕ = 7.0 mm, 2.67 g) of the same material. These parameters were applied if not stated otherwise. ^{1}H and ^{13}C liquid NMR spectra were recorded on a Varian 600 MHz and Bruker Avance III HD 600 MHz NMR spectrometer at 298 K and were calibrated using trimethylsylane (TMS). Proton chemical shifts are expressed in parts per million (ppm, δ scale) and are referred to the residual hydrogen in the solvent ($CHCl_3$, 7.27 ppm or DMSO 2.54 ppm). Data are represented as follows: chemical shift, multiplicity (s = singlet, d = doublet, t = triplet, q = quartet, m = multiplet and/or multiple resonances, bs = broad singlet, and combination of thereof), coupling constant (J) in Hertz (Hz) and integration. Carbon chemical shifts are expressed in parts per million (ppm, δ scale) and are referenced to the carbon resonances of the NMR solvent ($CDCl_3$, δ 77.0 ppm or δ DMSO-d_6 δ 39.5 ppm). Deuterated NMR solvents were obtained from Aldrich. Samples were analyzed using an Agilent 5977B MS interfaced to the GC 7890B equipped with a DB-5ms column (J & W), injector temperature at 230 °C, detector temperature at 280 °C, helium carrier gas flow rate of 1 ml/min. The GC oven temperature program was 60 °C initial temperature with 4 min hold time and ramping at 15°C/min to a final temperature of 270°C with 7 min hold time. 1 μL of each sample was injected in split (1:20) mode. After a solvent delay of 3 minutes mass spectra were acquired in full scan mode using 2.28 scans/s with a mass range of 50–500 Amu. Retention times of different compounds were determined by injecting pure compound under identical conditions. All the experiments were carried out in duplicate to ensure reproducibility of the experimental data. Yields refer to pure isolated materials.

2. Mechanochemical synthesis of 3a-j, 3o-p, 3s and 4d-n, 4o, 4q-r

General procedure **A** for anilines and *o*-phenylendiamines **3a-j**, **3o-p**, **3s** synthesis from 2-nitroanilines **2a-j**, **2o-p**, **2s**

A 10 mL stainless steel jar equipped with 2 stainless steel milling balls (7 mm diameter, 2.67 g) was filled with nitrobenzenes **2a-j**, **2o-p**, **2s** (1.00 mmol), NaOH (6.00 mmol), **1** (3.00 mmol) and 250 µL of distilled water. The vessel was then closed and the mechanochemical reaction was conducted, ranging from 60 to 180 min at a frequency of 30 Hz. Whenever necessary, further purification through flash column chromatography has been made. Lastly, the solvent was removed under reduced pressure to afford the pure anilines **3a-j**, **3o-p**, **3s**.

General procedure **B** for heterocycles **4l-n, 4q-r** synthesis from *o*-phenylenediamines **3l-n, 3q-r**

A 10 mL stainless steel jar equipped with 2 stainless steel milling balls (7.0 mm diameter, 2.67 g) was filled with *o*-phenylenediamines **3l-n**, **3q-r** (1.00 mmol), **1** (3.00 mmol) and 200 µL of distilled water. The vessel was then closed, and the mechanochemical reaction was conducted, ranging from 60 min to 180 min at a frequency of 30 Hz. At the end of the reaction, an additional silica pad (SiO_2, heptane/ethyl acetate/methanol = 1:1:0→6:3:1) was made to purify the reaction mixture. Lastly, the solvent was removed under reduced pressure to afford the pure heterocycle **4l-n**, **4q-r**.

General procedure **C** for heterocycles **4d-k, 4o** synthesis from 2-nitroanilines **2d-k, 2o**

A 10 mL stainless steel jar equipped with 2 stainless steel milling balls (7.0 mm diameter, 2.67 g) was filled with 2-nitroanilines **2d-k**, **2o** (1.00 mmol), NaOH (6.00 mmol), **1** (3.00 mmol) and 250 µL of distilled water. The vessel was then closed and the mechanochemical reaction was conducted, ranging from 60 to 180 min at a frequency of 30 Hz. At the end of the reaction, it was made an additional refill of **1** (3.00 mmol) and 60 µL of distilled water and the mechanochemical reaction was run ranging from 60 to 180 min at a frequency of 30 Hz. At the end of the reaction, an additional silica pad (SiO_2, hexane/ethyl acetate/methanol = 1:1:0→6:3:1) was made to purify the reaction mixture. Lastly, the solvent was removed under reduced pressure to afford the pure heterocycle **4d-k**, **4o**.

Aniline **3a**

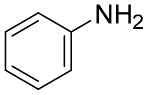

The title compound was synthesized according to the general procedure **A**. **2a** (123.11 mg, 1.00 mmol), NaOH (239.98 mg, 6.00 mmol), **1** (324.36 mg, 3.00 mmol), and 250 µL of distilled water were used, to afford **3a** as a colorless liquid (90.34 mg, 0.97 mmol, 97%).

^{1}H NMR (600 MHz, $CDCl_3$) δ = 7.20 (t, *J* = 9.0 Hz, 2H), 6.81 (t, 1H), 6.71 (d, *J* = 9.0 Hz, 2H), 3.65 (bs, 2H).

^{13}C NMR (151 MHz, $CDCl_3$) δ = 146.5, 129.3, 118.6, 115.2.
The spectroscopic data closely match the ones previously reported in the literature.[107]

2-Ethylaniline **3b**

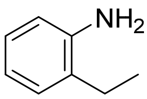

The title compound was synthesized according to the general procedure **A**. **2b** (151.2 mg, 1.0 mmol), NaOH (239.98 mg, 6.0 mmol), **1** (324.36 mg, 3.0 mmol), and 250 µL of distilled water were used, to afford **3b** as a colourless liquid (117.54 mg,

0.97 mmol, 97%).

^{1}H NMR (600 MHz, $CDCl_3$) δ = 7.10 – 7.07 (m, 1H), 7.05 (td, J = 7.6, 1.6 Hz, 1H), 6.76 (td, J = 7.6, 1.6 Hz, 1H), 6.69 (dd, J = 7.9, 1.3 Hz, 1H), 3.62 (s, 2H), 2.53 (q, J = 7.5 Hz, 2H), 1.26 (t, J = 7.5 Hz, 3H).

^{13}C NMR (151 MHz, $CDCl_3$) δ = 144.1, 128.5, 128.2, 126.9, 119.0, 115.5, 24.1, 13.1.

The spectroscopic data closely match the ones previously reported in the literature.[108]

4-Methoxyaniline **3c**

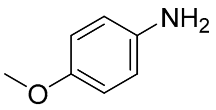

The title compound was synthesized according to the general procedure **A**. **2a** (153.14 mg, 1.00 mmol), NaOH (239.98 mg, 6.00 mmol), **1** (324.36 mg, 3.00 mmol), and 250 µL of distilled water were used, to afford **3a** as a colorless liquid (118.23 mg, 0.96 mmol, 96%).

^{1}H NMR (600 MHz, $CDCl_3$) δ = 6.75 (d, J = 8.8 Hz, 2H), 6.65 (d, J = 8.8 Hz, 2H), 3.75 (s, 3H), 3.35 (bs, 2H).

^{13}C NMR (151 MHz, $CDCl_3$) δ = 152.9, 140.1, 116.5, 114.9, 55.8.

The spectroscopic data closely match the ones previously reported in the literature.[108]

Benzene-1,2-diamine **3d**

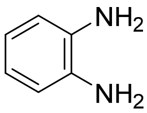

The title compound was synthesized according to the general procedure **A**. **2d** (138.13 mg, 1.00 mmol), NaOH (239.98 mg, 6.00 mmol), **1** (324.36 mg, 3.00 mmol), and 250 µL of distilled water were used, to afford **3d** as a light brown solid (105.98 mg, 0.98 mmol, 98%).

^{1}H NMR (600 MHz, $CDCl_3$ + DMSO-d_6) δ = 6.58 (dt, J = 6.0, 3.4 Hz, 2H), 6.51 (dd, J = 6.0, 4.9, 3.4 Hz, 2H).

^{13}C NMR (151 MHz, $CDCl_3$ + DMSO-d_6) δ = 134.5, 118.5, 115.5, 115.4.

The spectroscopic data closely match the previously reported in the literature[109].

3-Methylbenzene-1,2-diamine **3e**

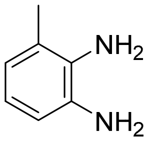

The title compound was synthesized according to the general procedure **A**. **2e** (152.2 mg, 1.00 mmol), NaOH (239.98 mg, 6.00 mmol), **1** (324.36 mg, 3.00 mmol) and 250 µL

of distilled water were used, to afford **3e** as a brownish solid (118.50 mg, 0.97 mmol, 97%).

^{1}H NMR (600 MHz, CDCl$_3$) δ = 6.67 – 6.71 (m, 3H), 3.38 (bs, 4H), 2.21 (s, 3H).

^{13}C NMR (151 MHz, CDCl$_3$) δ = 134.0, 133.6, 123.5, 122.2, 119.2, 115.2, 17.5.

The spectroscopic data closely match the ones previously reported in the literature [110].

N-Methylbenzene-1,2-diamine **3f**

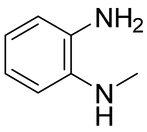

The title compound was synthesized according to the general procedure **A**. **2f** (152.15 mg, 1.00 mmol), NaOH (239.98 mg, 6.00 mmol), **1** (324.36 mg, 3.00 mmol) and 250 µL of distilled water were used, to afford **3f** as a black liquid (117.28 mg, 0.96 mmol, 96%).

^{1}H NMR (600 MHz, CDCl$_3$) δ = 6.96 (ddd, *J* = 7.8, 6.7, 2.2, Hz, 1H), 6.82 – 6.72 (m, 3H), 3.41 (s, 3H), 2.91 (bs, 3H).
^{13}C NMR (151 MHz, CDCl$_3$) δ = 138.9, 134.1, 120.7, 118.3, 116.2, 110.9, 30.9.

The spectroscopic data closely match the ones previously reported in the literature [111].

4-Bromobenzene-1,2-diamine **3g**

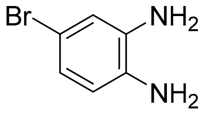

The title compound was synthesized according to the general procedure **A**. **2a** (217.02 mg, 1.00 mmol), NaOH (239.98 mg, 6.00 mmol), **1** (324.36 mg, 3.00 mmol), and 250 µL of distilled water were used, to afford **3a** as a yellowish solid (181.43 mg, 0.97 mmol, 97%).

^{1}H NMR (600 MHz, CDCl$_3$) δ = 6.74 – 6.69 (m, 3H), 3.28 (bs, 4H).

^{13}C NMR (151 MHz, CDCl$_3$) δ = 134.9, 120.4, 116.9.

The spectroscopic data closely match the ones previously reported in the literature [112].

4,5-Dichlorobenzene-1,2-diamine **3h**

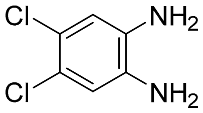

The title compound was synthesized according to the general procedure **A**. **2h** (207.01 mg, 1.00 mmol), NaOH (239.98 mg, 6.00 mmol), **1** (324.36 mg, 3.00 mmol), and 250 µL of distilled water were used, to afford **3h** as a yellow solid (168.18 mg, 0.95 mmol, 95%).
^{1}H NMR (600 MHz, DMSO-*d*$_6$) δ = 6.62 (s, 2H), 4.85 (bs, 4H).

^{13}C NMR (151 MHz, DMSO-*d*$_6$) δ = 135.6, 117.3, 114.3.

The spectroscopic data closely match the ones previously reported in the literature [113].

4-Methoxybenzene-1,2-diamine **3i**

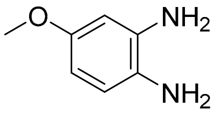

The title compound was synthesized according to the general procedure **A**. **2i** (168.15 mg, 1.00 mmol), NaOH (239.98 mg, 6.00 mmol), **1** (324.36 mg, 3.00 mmol), and 250 μL of distilled water were used, to afford **3i** as a dark red solid (105.01 mg, 0.76 mmol, 76%).

^{1}H NMR (600 MHz, $CDCl_3$) δ = δ 6.63 (d, J = 8.4 Hz, 1H), 6.32 (d, J = 2.8 Hz, 1H), 6.26 (dd, J = 8.4, 2.7 Hz, 1H), 3.72 (s, 3H).

^{13}C NMR (151 MHz, $CDCl_3$) δ = 154.6, 137.1, 127.4, 118.3, 104.1, 103.0, 55.6.

The spectroscopic data closely match the ones previously reported in the literature [114].

4-(Trifluoromethoxy) benzene-1,2-diamine **3j**

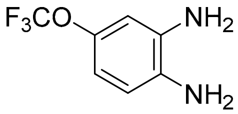

The title compound was synthesized according to the general procedure **A**. **2j** (222.12 mg, 1.00 mmol), NaOH (239.98 mg, 6.00 mmol), **1** (324.36 mg, 3.00 mmol), and 250 μL of distilled water were used, to afford **3j** as a yellow solid (178.69 mg, 0.93 mmol, 93%).

^{1}H NMR (600 MHz, $CDCl_3$) δ =6.65 (d, J = 8.1 Hz, 1H), 6.58 – 6.56 (m, 2H), 3.40 (bs, 4H).

^{13}C NMR (151 MHz, $CDCl_3$) δ = 142.9, 136.0, 133.2, 121.6, 119.9, 117.0, 112.4, 109.6.

^{19}F NMR (565 MHz, $CDCl_3$) δ = -58.19.

HRMS: calculated for $C_7H_7F_3N_2O$: 193.0589 [M+H]$^+$; found: 193.0580.

Pyridine-2,3-diamine **3o**

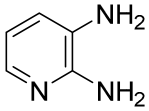

The title compound was synthesized according to the general procedure **A**. **2o** (139.11 mg, 1.00 mmol), NaOH (239.98 mg, 6.00 mmol), **1** (324.36 mg, 3.00 mmol), and 250 μL of distilled water were used, to afford **3o** as a yellow solid (103.67 mg, 0.95 mmol, 95%).

^{1}H NMR (600 MHz, $CDCl_3$) δ = 7.65 (dd, J = 5.0, 1.5 Hz, 1H), 6.88 (dd, J = 7.5, 1.5 Hz, 1H), 6.62 (dd, J = 7.5, 5.0 Hz, 1H), 4.26 (bs, 2H), 3.31 (bs, 2H).

^{13}C NMR (151 MHz, $CDCl_3$) δ = 149.4, 138.9, 129.2, 122.6, 115.8.

The spectroscopic data closely match the ones previously reported in the literature [112].

N[1]-Benzylbenzene-1,2-diamine **3p**

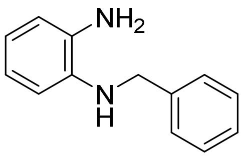

The title compound was synthesized according to the general procedure **A**. **2p** (228.25 mg, 1.00 mmol), NaOH (239.98 mg, 6.00 mmol), **1** (324.36 mg, 3.00 mmol), and 250 µL of distilled water were used, to afford **3p** as a white solid (158.62 mg, 0.80 mmol, 80%).

^{1}H NMR (600 MHz, CDCl$_3$) δ = 7.42 – 7.40 (m, 2H), 7.36 (dd, *J* = 8.4, 6.8 Hz, 2H), 7.30 (d, *J* = 7.3 Hz, 1H), 6.81 (td, *J* = 7.5, 1.6 Hz, 1H), 6.75 (dd, *J* = 7.6, 1.6 Hz, 1H), 6.72 – 6.68 (m, 2H), 4.33 (s, 2H), 3.47 (bs, 3H).

^{13}C NMR (151 MHz, CDCl$_3$) δ = 139.6, 137.9, 134.3, 128.8, 127.9, 127.4, 120.9, 119.0, 116.7, 112.2, 48.8.

The spectroscopic data closely match the ones previously reported in the literature [115].

(3-Amino-4-chlorophenyl) (phenyl)methanone **3s**

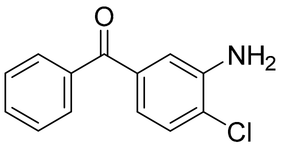

The title compound was synthesized according to the general procedure **A**. **2s** (261.66 mg, 1.00 mmol), NaOH (239.98 mg, 6.00 mmol), **1** (324.36 mg, 3.00 mmol), and 250 µL of distilled water were used, to afford **3s** as a yellowish solid (220.10 mg, 0.94 mmol, 94%).

^{1}H NMR (600 MHz, CDCl$_3$) δ = 7.79 – 7.77 (m, 2H), 7.60 – 7.57 (m, 1H), 7.47 (t, *J* = 7.7 Hz, 2H), 7.34 (d, *J* = 8.2 Hz, 1H), 7.22 (d, J = 2.0 Hz, 1H), 7.07 (dd, *J* = 8.2, 2.0 Hz, 1H), 4.21 (bs, 2H).

^{13}C NMR (151 MHz, CDCl$_3$) δ = 196.1, 143.1, 137.6, 137.2, 132.6, 130.1, 129.3, 128.4, 123.6, 120.9, 116.9.

HRMS: calculated for $C_{13}H_{10}ClNO$: 232.0529 [*M*+H]$^+$; found: 232.0523.

1H-Benzo[d]imidazole **4d**

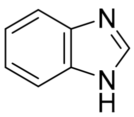

The title compound was synthesized according to the general procedure **C**. **2d** (138.13 mg, 1.00 mmol), NaOH (239.98 mg, 6.00 mmol), **1** (324.36 mg, 3.00 mmol), and 250 µL of distilled water were used. After that, **1** (324.36 mg, 3.00 mmol) and 60 µL of distilled water were added. Lastly, an additional silica pad was made to afford **4d** as a white solid (111.05 mg, 0.94 mmol, 94%).

^{1}H NMR (600 MHz, DMSO-*d$_6$*) δ = 12.42 (bs, 1H), 8.20 (s, 1H), 7.58 (s, 2H), 7.19 – 7.17 (m, 2H).

^{13}C NMR (151 MHz, DMSO-*d$_6$*) δ = 141.9, 122.1, 121.3, 119.0, 111.6.

The spectroscopic data closely match the ones previously reported in the literature [116].

4-Methyl-1H-benzo[d]imidazole **4e**

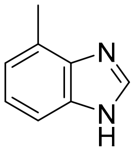

The title compound was synthesized according to the general procedure **C**. **2e** (152.15 mg, 1.00 mmol), NaOH (239.98 mg, 6.00 mmol), **1** (324.36 mg, 3.00 mmol), and 250 µL of distilled water were used. After that, **1** (324.36 mg, 3.00 mmol) and 60 µL of distilled water were added. Lastly, an additional silica pad was made to afford **4e** as a white solid (120.27 mg, 0.91 mmol, 91%).

^{1}H NMR (600 MHz, DMSO-*d$_6$*) δ = 12.44 (bs, 1H), 8.17 (s, 1H), 7.39 (d, *J* = 8.0 Hz, 1H), 7.07 (t, *J* = 7.6 Hz, 1H), 6.98 (d, *J* = 7.2 Hz, 1H), 2.52 (s, 3H).

^{13}C NMR (151 MHz, DMSO-*d$_6$*) δ = 141.3, 122.0, 121.7, 16.8.

The spectroscopic data closely match the ones previously reported in the literature [117].

4-Methyl-1H-benzo[d]imidazole **4f**

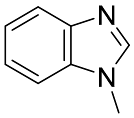

The title compound was synthesized according to the general procedure **C**. **2f** (152.15 mg, 1.00 mmol), NaOH (239.98 mg, 6.00 mmol), **1** (324.36 mg, 3.00 mmol) and 250 µL of distilled water were used. After that, **1** (324.36 mg, 3.00 mmol) and 60 µL of distilled water were added. Lastly, an additional silica pad was made for affording **4f** as a white solid (122.92 mg, 0.93 mmol, 93%).

^{1}H NMR (600 MHz, DMSO-*d$_6$*) δ = 8.17 (bs, 1H), 7.67 – 7.63 (m, 1H), 7.55 (dd, *J* = 8.0, 1.2 Hz, 1H), 7.27 (t, *J* = 7.5 Hz, 1H), 7.21 (td, *J* = 7.5, 1.2 Hz, 1H), 3.83 (s, 3H).

^{13}C NMR (151 MHz, DMSO-*d$_6$*) δ = 144.5, 143.3, 134.6, 122.2, 121.4, 119.3, 110.1, 30.6.

The spectroscopic data closely match the ones previously reported in the literature [117].

4-Methyl-1H-benzo[d]imidazole **4g**

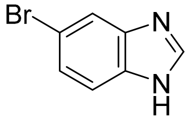

The title compound was synthesized according to the general procedure **C**. **2g** (217.02 mg, 1.00 mmol), NaOH (239.98 mg, 6.00 mmol), **1** (324.36 mg, 3.00 mmol), and 250 µL of distilled water were used. After that, **1** (324.36 mg, 3.00 mmol) and 60 µL of distilled water were added. Lastly, an additional silica pad was made to afford **4g** as a white solid (181.28 mg, 0.92 mmol, 92%).

^{1}H NMR (600 MHz, DMSO-*d$_6$*) δ = 12.61 (s, 1H), 8.25 (s, 1H), 7.79 (s, 1H), 7.55 (d, *J* = 8.5 Hz, 1H), 7.32 (dd, *J* = 8.5, 1.9 Hz, 1H).

^{13}C NMR (151 MHz, DMSO-*d$_6$*) δ = 143.3, 124.7, 114.1.
The spectroscopic data closely match the ones previously reported in the literature [118].

5,6-Dichloro-1H-benzo[d]imidazole **4h**

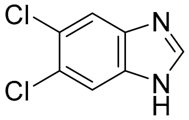

The title compound was synthesized according to the general procedure **C**. **2h** (207.01 mg, 1.00 mmol), NaOH (239.98 mg, 6.00 mmol), **1** (324.36 mg, 3.00 mmol)

and 250 μL of distilled water were used. After that, **1** (324.36 mg, 3.00 mmol) and 60 μL of distilled water were added. Lastly, an additional silica pad was made for affording **4h** as a white solid (168.32 mg, 0.90 mmol, 90%).

^{1}H NMR (600 MHz, DMSO-*d_6*) δ = 8.33 (s, 1H), 7.86 (s, 2H).

^{13}C NMR (151 MHz, DMSO-*d_6*) δ = 144.8, 124.3, 116.6.

The spectroscopic data closely match the ones previously reported in the literature [119].

5-Methoxy-1H-benzo[d]imidazole **4i**

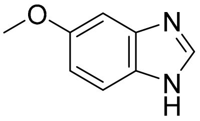

The title compound was synthesized according to the general procedure **C**. **2i** (168.15 mg, 1.00 mmol), NaOH (239.98 mg, 6.00 mmol), **1** (324.36 mg, 3.00 mmol), and 250 μL of distilled water were used. After that, **1** (324.36 mg, 3.00 mmol) and 60 μL of distilled water were added. Lastly, an additional silica pad was made to afford **4i** as a reddish solid (100.76 mg, 0.68 mmol, 68%).

^{1}H NMR (600 MHz, DMSO-*d_6*) δ = 8.10 (d, *J* = 5.1 Hz, 1H), 7.47 (dd, *J* = 8.7, 2.7 Hz, 1H), 7.08 (t, *J* = 2.9 Hz, 1H), 6.81 (dd, *J* = 8.7, 2.7 Hz, 1H), 3.77 (s, 3H).

^{13}C NMR (151 MHz, DMSO-*d_6*) δ = 155.6, 141.5, 116.4, 111.4, 97.5, 55.4.

The spectroscopic data closely match the ones previously reported in the literature [117].

5-(Trifluoromethoxy)-1H-benzo[d]imidazole **4j**

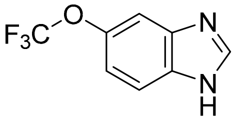

The title compound was synthesized according to the general procedure **C**. **2j** (222.12 mg, 1.00 mmol), NaOH (239.98 mg, 6.00 mmol), **1** (324.36 mg, 3.00 mmol), and 250 μL of distilled water were used. After that, **1** (324.36 mg, 3.00 mmol) and 60 μL of distilled water were added. Lastly, an additional silica pad was made to afford **4j** as a brownish solid (185.97 mg, 0.92 mmol, 92%).

^{1}H NMR (600 MHz, DMSO-*d_6*) δ = 12.69 (s, 1H), 8.34 (s, 1H), 7.67 (d, *J* = 8.7 Hz, 1H), 7.59 (d, *J* = 2.3 Hz, 1H), 7.18 (dd, *J* = 8.7, 2.3 Hz, 1H).

^{13}C NMR (151 MHz, DMSO-*d_6*) δ = 144.2, 143.6, 122.9, 121.3, 119.6, 115.6.

The spectroscopic data closely match the ones previously reported in the literature [120].

1H-Benzo[d]imidazol-5-amine **4k**

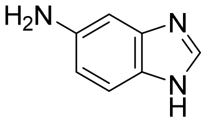

The title compound was synthesized according to the general procedure **C**. **2k** (153.14 mg, 1.00 mmol), NaOH (239.98 mg, 6.00 mmol), **1** (324.36 mg, 3.00

mmol), and 250 μL of distilled water were used. After that, **1** (324.36 mg, 3.00 mmol) and 60 μL of distilled water were added. Lastly, an additional silica pad was made to afford **4k** as a brownish solid (59.9 mg, 0.45 mmol, 45%).

^{1}H NMR (600 MHz, DMSO-*d$_6$*) δ = 7.85 (s, 1H), 7.24 (d, *J* = 8.5 Hz, 1H), 6.66 (s, 1H), 6.56 (s, 2H), 6.51 (dd, *J* = 8.5, 2.1 Hz, 1H).

^{13}C NMR (151 MHz, DMSO-*d$_6$*) δ = 144.6, 139.1, 134.6, 118.9, 111.3, 94.8.

The spectroscopic data closely match the ones previously reported in the literature [116].

1H-Benzo[d]imidazole-5-carbonitrile **4l**

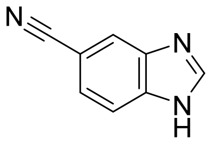

The title compound was synthesized according to the general procedure **B**. **3l** (133.15 mg, 1.00 mmol), **1** (324.36 mg, 3.00 mmol), and 200 μL of distilled water were used. Lastly, an additional silica pad was made to afford **4l** as a brownish solid (35.79 mg, 0.25 mmol, 25%).

^{1}H NMR (600 MHz, DMSO-*d$_6$*) δ = 8.46 (s, 1H), 8.15 (m, 1H), 7.75 (dd, *J* = 8.3, 0.7 Hz, 1H), 7.58 (dd, *J* = 8.3, 1.5 Hz, 1H).

^{13}C NMR (151 MHz, DMSO-*d$_6$*) δ = 145.3, 125.3, 121.4, 120.0, 116.0, 103.8.

The spectroscopic data closely match the ones previously reported in the literature [121].

5-Nitro-1H-benzo[d]imidazole **4m**

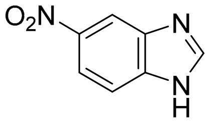

The title compound was synthesized according to the general procedure **B**. **3m** (153.14 mg, 1.00 mmol), **1** (324.36 mg, 3.00 mmol), and 200 μL of distilled water were used. Lastly, an additional silica pad was made to afford **4m** as a brownish solid (16.31 mg, 0.10 mmol, 10%).

^{1}H NMR (600 MHz, DMSO-*d$_6$*) δ = 8.54 (s, 1H), 8.50 (t, *J* = 1.7 Hz, 1H), 8.11 – 8.09 (m, 1H), 7.76 (dd, J = 8.8, 1.3 Hz, 1H).

^{13}C NMR (151 MHz, DMSO-*d$_6$*) δ = 146.8, 142.7, 117.6, 115.0, 112.8.

The spectroscopic data closely match the ones previously reported in the literature [116].

1-Phenyl-1H-benzo[d]imidazole **4n**

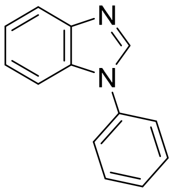

The title compound was synthesized according to the general procedure **B**. **3n** (184.24 mg, 1.00 mmol), **1** (324.36 mg, 3.00 mmol), and 200 μL of distilled

water were used. Lastly, an additional silica pad was made to afford **4n** as a pink solid (23.31 mg, 0.12 mmol, 12%).

^{1}H NMR (600 MHz, DMSO-d_6) δ = 8.55 (s, 1H), 7.82 – 7.76 (m, 1H), 7.67 – 7.63 (m, 2H), 7.62 – 7.56 (m, 3H), 7.49 – 7.45 (m, 1H), 7.33 – 7.28 (m, 2H).

^{13}C NMR (151 MHz, DMSO-d_6) δ = 143.9, 143.3, 136.0, 133.1, 130.1, 127.8, 123.7, 123.5, 122.5, 120.0, 110.7.

The spectroscopic data closely match the ones previously reported in the literature [117].

3H-Imidazo[4,5-b]pyridine **4o**

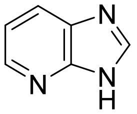

The title compound was synthesized according to the general procedure **C**. **2o** (139.11 mg, 1.00 mmol), NaOH (239.98 mg, 6.00 mmol), **1** (324.36 mg, 3.00 mmol), and 250 µL of distilled water were used. After that, **1** (324.36 mg, 3.00 mmol) and 60 µL of distilled water were added. Lastly, an additional silica pad was made to afford **4o** as a white solid (66.71 mg, 0.56 mmol, 56%).

^{1}H NMR (600 MHz, DMSO-d_6) δ = 12.95 (bs, 1H), 8.52 – 8.29 (m, 2H), 8.01 (d, *J* = 8.1 Hz, 1H), 7.23 (dd, *J* = 8.0, 4.7 Hz, 1H).

^{13}C NMR (151 MHz, DMSO-d_6) δ = 143.9, 143.7, 139.6, 117.8, 116.6, 114.4.

The spectroscopic data closely match the ones previously reported in the literature [120].

6-Bromo-3H-imidazo[4,5-b]pyridine **4q**

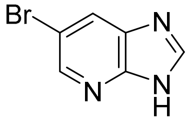

The title compound was synthesized according to the general procedure **B**. **3n** (188.03 mg, 1.00 mmol), **1** (324.36 mg, 3.00 mmol), and 200 µL of distilled water were used. Lastly, an additional silica pad was made to afford **4n** as a pink solid (21.78 mg, 0.11 mmol, 11%).

^{1}H NMR (600 MHz, DMSO-d_6) δ = 8.48 (s, 1H), 8.43 (d, *J* = 2.2 Hz, 1H), 8.29 (d, *J* = 2.2 Hz, 1H).

^{13}C NMR (151 MHz, DMSO-d_6) δ = 146.0, 144.6, 126.7, 113.2.

The spectroscopic data closely match the ones previously reported in the literature [122].

1H-Perimidine **4r**

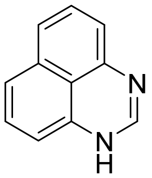

The title compound was synthesized according to the general procedure **B**. **3r** (158.20 mg, 1.00 mmol), **1** (324.36 mg, 3.00 mmol), and 200 µL of distilled

water were used. Lastly, an additional silica pad was made to afford **4r** as a purple solid (100.92 mg, 0.60 mmol, 60%).

^{1}H NMR (600 MHz, DMSO-*d*$_6$) δ = 7.29 (s, 1H), 7.07 (t, *J* = 7.9 Hz, 2H), 6.97 (d, *J* = 8.3 Hz, 2H), 6.48 – 6.30 (m, 2H).

^{13}C NMR (151 MHz, DMSO-*d*$_6$) δ = 146.5, 135.4, 128.4, 123.0, 118.4.

The spectroscopic data closely match the ones previously reported in the literature [123].

3. Green Metrics calculations

Calculation of the Green Chemistry Metrics for the Mechanochemical Preparation of **4d**

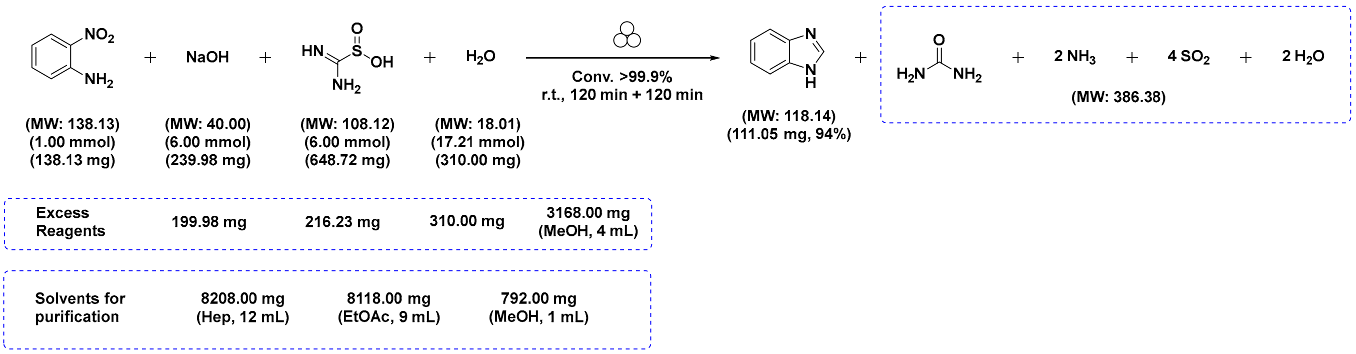

Scheme **M1**. Mechanochemical preparation of **4d**.

Calculation of Green Chemistry Metrics

$$\text{Atom Economy} = \frac{\text{Mass of desired useful product}}{\text{Total Mass of all reactants}} \times 100 = \frac{118.14}{138.13 + 40.00 + 108.12 + 18.01} \times 100 = 38.8\%$$

$$\text{Environmental Factor} = \frac{\text{Mass of total waste}}{\text{Mass of desired product}} = \frac{199.98 + 216.23 + 310.00 + 386.38 + 3168.00 + 17118.00}{111.05} = 192.7$$

$$\text{Reaction Mass Efficiency} = \frac{\text{actual mass of desired product}}{\text{mass of reactants}} \times 100 = \frac{111.05}{138.13 + 239.98 + 648.72 + 310.00} \times 100 = 8.31\%$$

DOZN™ score for the mechanochemical synthesis of 4d

DOZN SCORE RESULT

Download

Aggregate Score

2

Scoring Matrix

1 … 100

Principle	Score
#1 Prevention	0.00
#2 Atom Economy	6.58
#3 Less Hazardous Chemical Synthesis	2.63
#4 Designing Safer Chemicals	0.40
#5 Safer Solvents and Auxiliaries	0.00
#6 Design for Energy Efficiency	0.00
#7 Use of Renewable Feedstocks	5.05
#8 Reduce Derivatives	0.00
#9 Catalysis	0.55
#10 Design for Degradation	0.78
#11 Real-time analysis for Pollution Prevention	0.00
#12 Inherently Safer Chemistry for Accident Prevention	2.63

Note: For more information on above Principles (click here)

Groups	Principles	Score
#1 Improved Resource Use	1, 2, 7, 8, 9, 11	2.03
#2 Increased Energy Efficiency	6	0.00
#3 Reduced Human and Environmental Hazards	3, 4, 5, 10, 12	1.28

Note: The intent of the DOZN tool is to compare relative "greenness" for similar products or processes, as indicated by a lower DOZN score.

Figure S1

Calculation of the Green Chemistry Metrics for the microwave preparation of **4d**

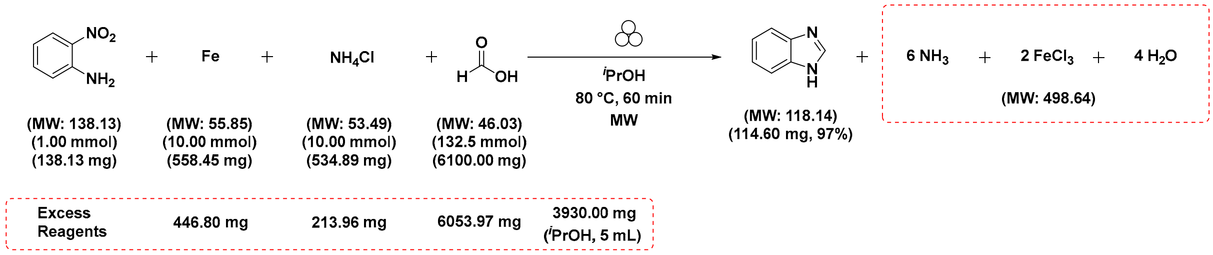

Scheme M2. Microwave preparation of benzimidazole **4d** [20].

To the total amount of waste, the following quantities must be added:

1) 10 mL of 2-propanol (**7860.00 mg**) used for diluting the reaction mixture at the end of the reaction;
2) 20 mL of dichloromethane (**26600.00 mg**) and 5 mL of sat. aq $NaHCO_3$. With regards to the quantity of $NaHCO_3$, we based our calculations on the solubility of $NaHCO_3$ in water:

Solubility of $NaHCO_3$ in water = 96 mg/mL
Quantity of $NaHCO_3$ in a 5 mL sat. solution = 96 mg/mL*5 mL = 480 mg $NaHCO_3$
Weight of a 5 mL sat. aq $NaHCO_3$ = 1,00 mg/mL*5 mL + 480 mg $NaHCO_3$ = **5480 mg**

3) 100 mL of dichloromethane (**133000.00 mg**) for extracting the compound from the aqueous phase.
4) Quantity of $MgSO_4$ used for drying the organic mixture (NO DATA). In this case, we postulated it by considering as follows:

The quantity of water that can be found in dichloromethane is generally not higher than the 0.24% of the entire mass considered [124]. So, we assigned the quantity of anhydrous $MgSO_4$ used based on such data. Herein, we report all the calculi.

159600.00 mg CH_2Cl_2 : 100 = **X** mg of H_2O dissolved in 120 mL of CH_2Cl_2 : 0.24

X= 383.04 mg of H_2O dissolved in 120 mL of CH_2Cl_2

Now we estimate the moles of H_2O dissolved in 120 mL CH_2Cl_2.

18.00 mg H_2O : 1.00 mmol = 383.04 mg of H_2O dissolved in 120 mL of CH_2Cl_2: **X** mmol of H_2O dissolved in 120 mL of CH_2Cl_2

X= 21.28 mmol of H_2O dissolved in 120 mL of CH_2Cl_2

Considering the drying capacity of anhydrous $MgSO_4$ (1 mol of $MgSO_4$ is able to complex 7 mol of H_2O [124]), we can then evaluate the mg of anhydrous $MgSO_4$ necessary for the drying step:

$$MgSO_4 + 7\,H_2O \longrightarrow MgSO_4 \cdot 7\,H_2O$$

Scheme M3. $MgSO_4$ drying ability (CIT.)

1.00 mmol of $MgSO_4$: 7.00 mmol of H_2O = **X** mmol of $MgSO_4$ required : 21.28 mmol of H_2O dissolved in 120 mL of CH_2Cl_2

X= 3.04 mmol of $MgSO_4$ required

1.00 mmol of $MgSO_4$: 120.37 mg of $MgSO_4$ = 3.04 mmol of $MgSO_4$ required : **X** mg of $MgSO_4$ required

X= **365.92** mg of $MgSO_4$ required

Calculation of Green Chemistry Metrics

$$\textbf{A}\text{tom }\textbf{E}\text{conomy} = \frac{\text{Mass of desired useful product}}{\text{Total Mass of all reactants}} \times 100 = \frac{118.14}{138.13 + 55.85 + 53.49 + 46.03} \times 100 = 40.3\%$$

$$\textbf{E}\text{nvironmental }\textbf{F}\text{actor} = \frac{\text{Mass of total waste}}{\text{Mass of desired product}}$$

$$\textbf{EF} = \frac{446.80 + 213.96 + 6053.97 + 3930.00 + 498.64 + 7860 + 26600 + 5480 + 133000 + 365.92}{114.60} = 1609.5$$

$$\textbf{R}\text{eaction }\textbf{M}\text{ass }\textbf{E}\text{fficiency} = \frac{\text{actual mass of desired product}}{\text{mass of reactants}} \times 100$$

$$\textbf{RME} = \frac{114.60}{138.13 + 558.45 + 534.89 + 6100} \times 100 = 1.6\%$$

DOZN™ score for the microwave synthesis of 4d

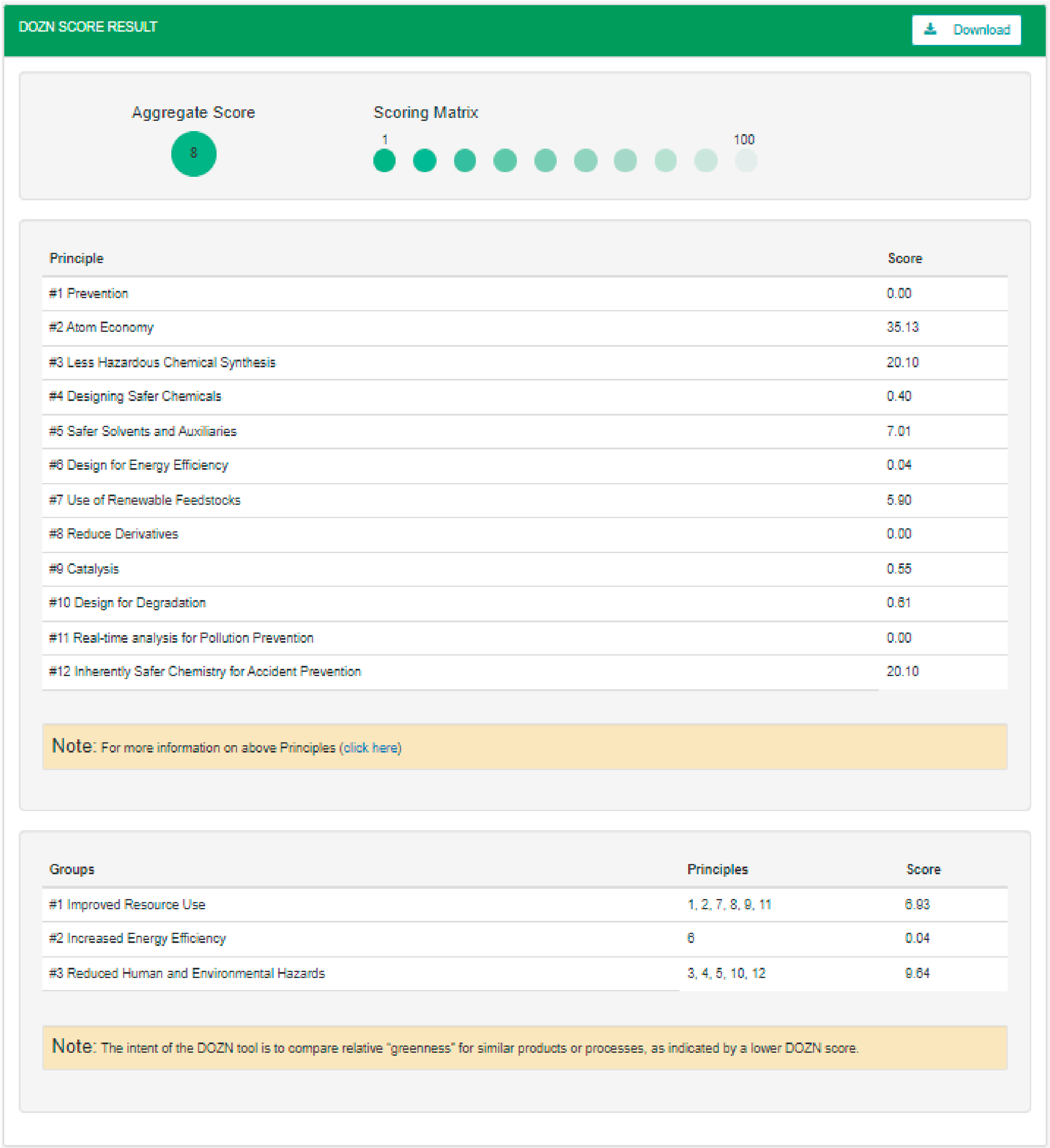
DOZN SCORE RESULT

Download

Aggregate Score

8

Scoring Matrix

1 100

Principle	Score
#1 Prevention	0.00
#2 Atom Economy	35.13
#3 Less Hazardous Chemical Synthesis	20.10
#4 Designing Safer Chemicals	0.40
#5 Safer Solvents and Auxiliaries	7.01
#6 Design for Energy Efficiency	0.04
#7 Use of Renewable Feedstocks	5.90
#8 Reduce Derivatives	0.00
#9 Catalysis	0.55
#10 Design for Degradation	0.61
#11 Real-time analysis for Pollution Prevention	0.00
#12 Inherently Safer Chemistry for Accident Prevention	20.10

Note: For more information on above Principles (click here)

Groups	Principles	Score
#1 Improved Resource Use	1, 2, 7, 8, 9, 11	6.93
#2 Increased Energy Efficiency	6	0.04
#3 Reduced Human and Environmental Hazards	3, 4, 5, 10, 12	9.64

Note: The intent of the DOZN tool is to compare relative "greenness" for similar products or processes, as indicated by a lower DOZN score.

Figure S2

4. Spectra

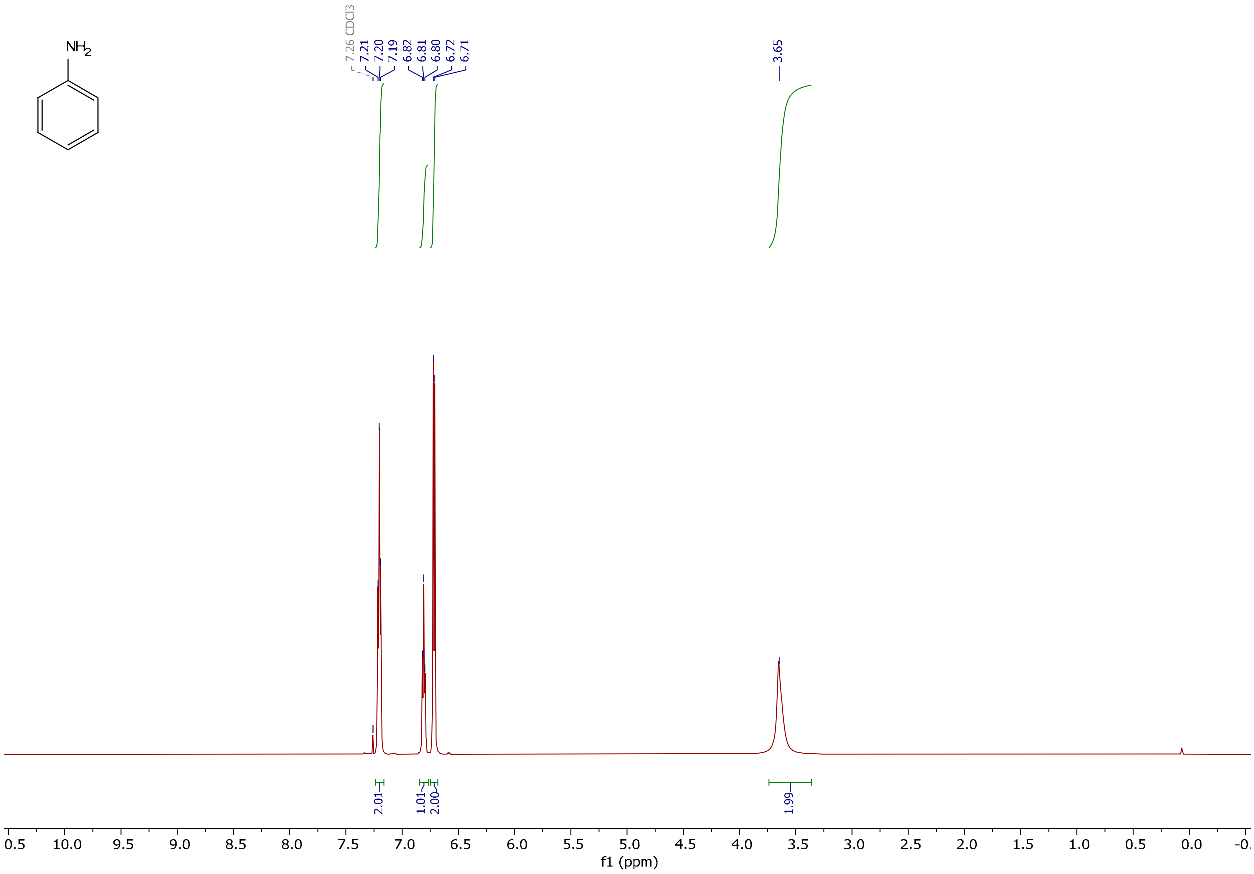
NH2
7.26 CDCl3
7.21
7.20
7.19
6.82
6.81
6.80
6.72
6.71
3.65
2.01
1.01
2.00
1.99
f1 (ppm)

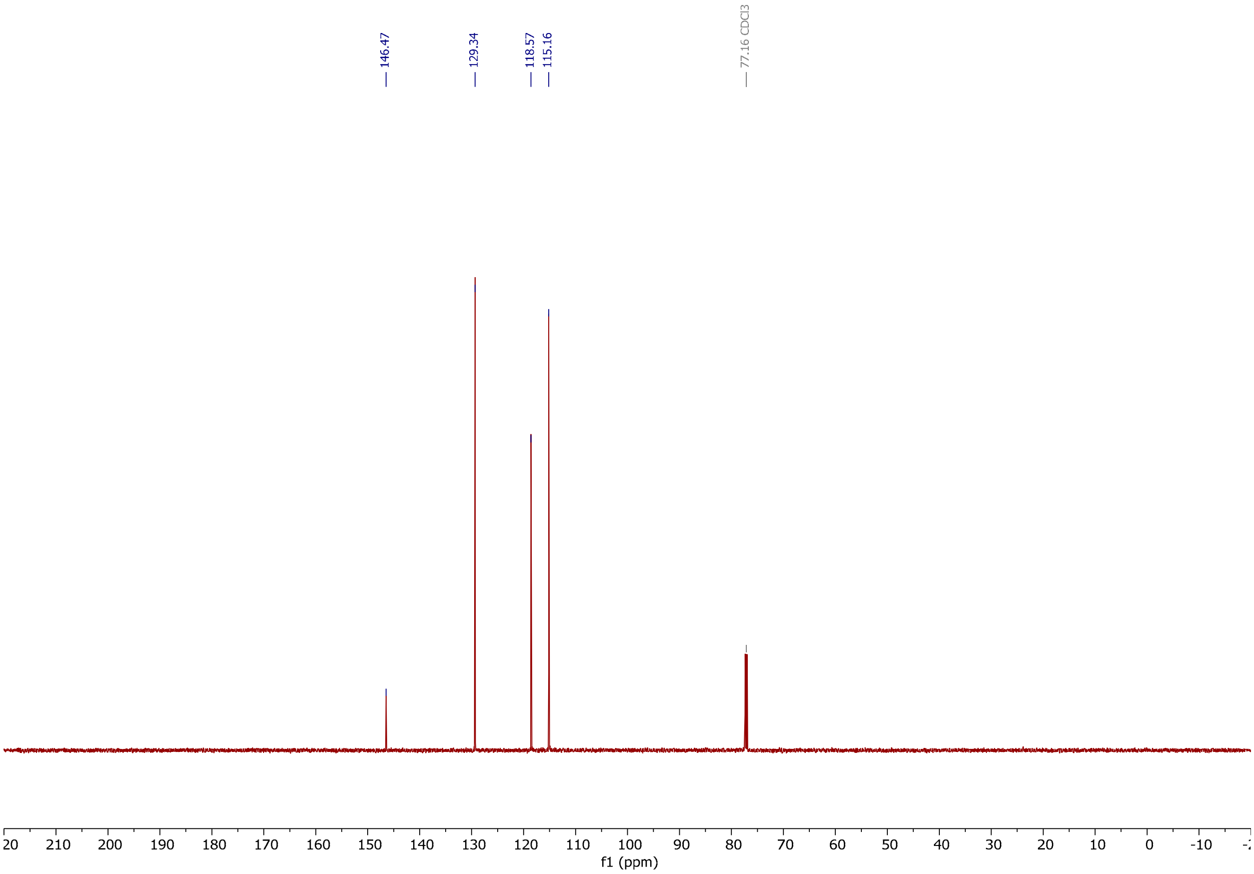
146.47
129.34
118.57
115.16
77.16 CDCl3
f1 (ppm)

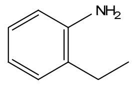
NH2

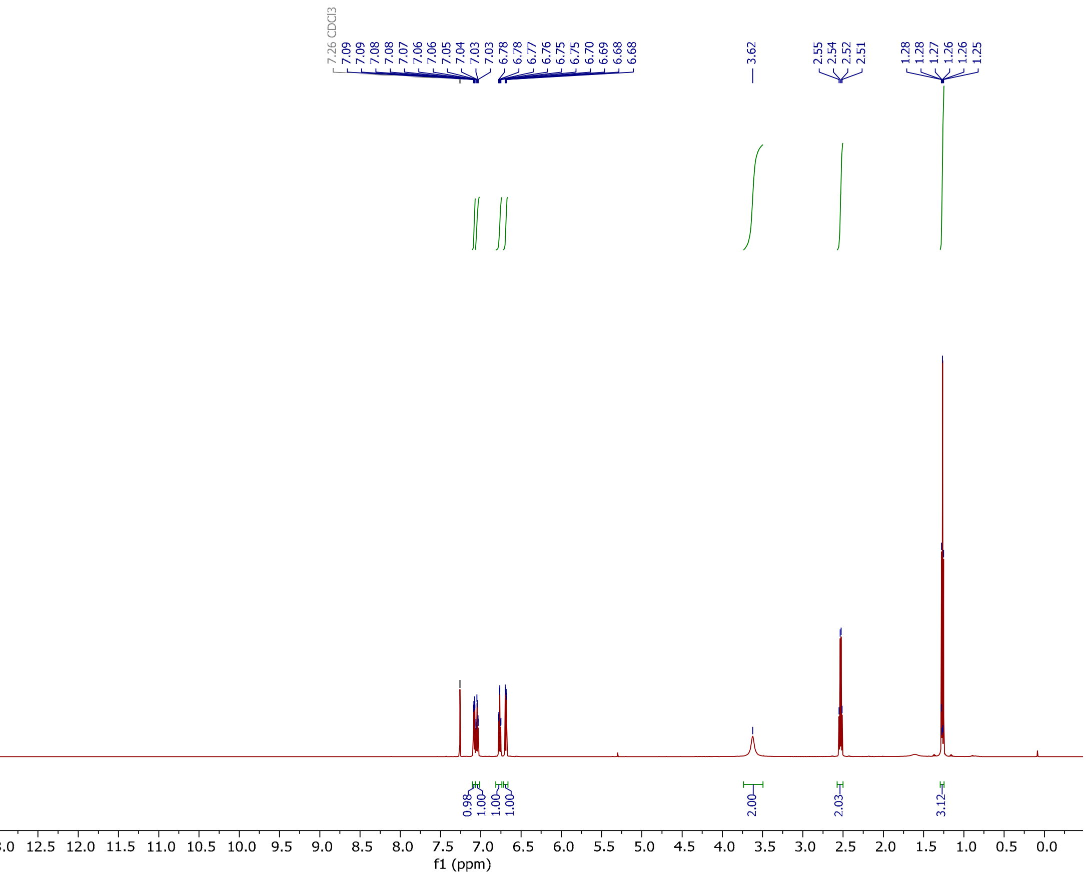
7.26 CDCl3
7.09
7.09
7.08
7.07
7.06
7.06
7.05
7.04
7.03
7.03
6.78
6.78
6.77
6.76
6.75
6.75
6.70
6.69
6.68
6.68
3.62
2.55
2.54
2.52
2.51
1.28
1.28
1.27
1.26
1.26
1.25
0.98
1.00
1.00
1.00
2.00
2.03
3.12
14.5 14.0 13.5 13.0 12.5 12.0 11.5 11.0 10.5 10.0 9.5 9.0 8.5 8.0 7.5 7.0 6.5 6.0 5.5 5.0 4.5 4.0 3.5 3.0 2.5 2.0 1.5 1.0 0.5 0.0
f1 (ppm)

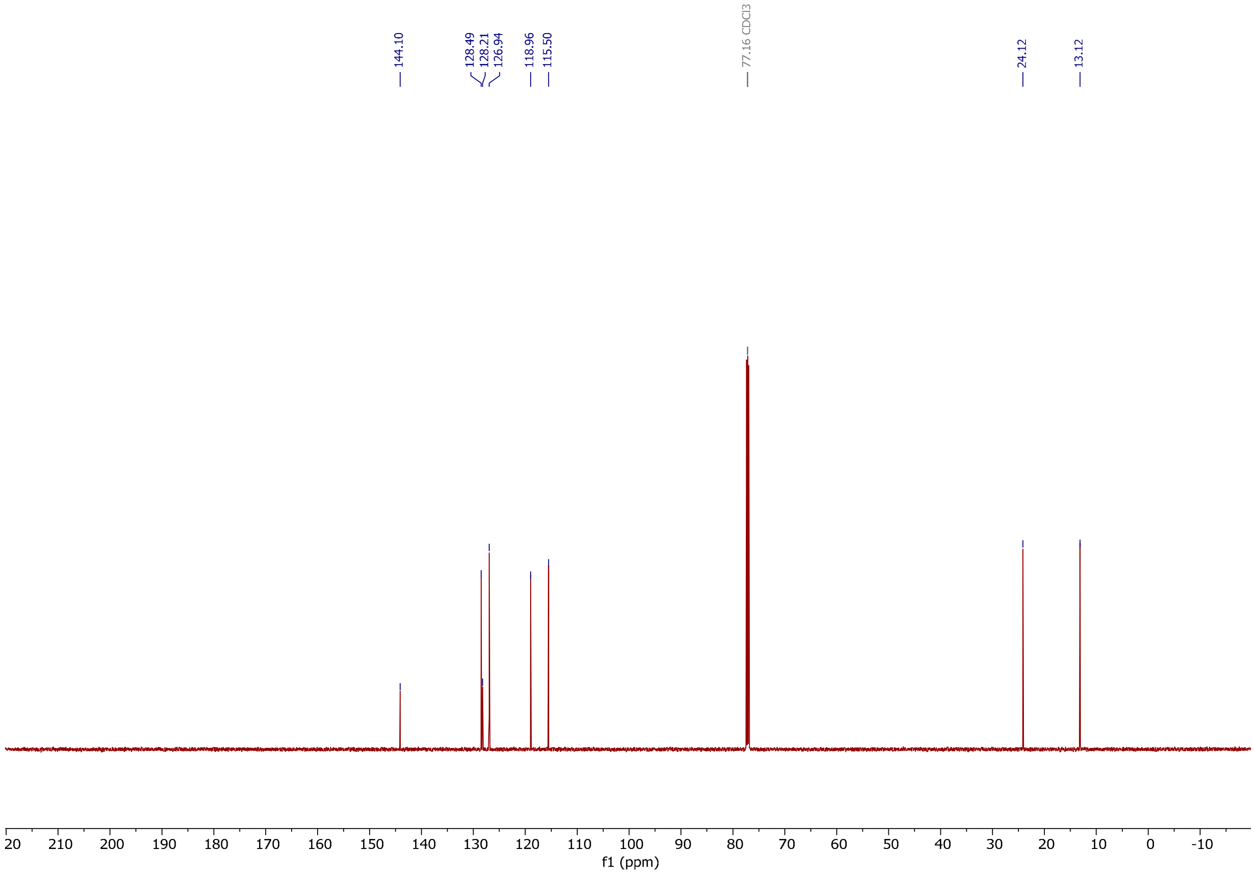
144.10
128.49
128.21
126.94
118.96
115.50
77.16 CDCl3
24.12
13.12
20 210 200 190 180 170 160 150 140 130 120 110 100 90 80 70 60 50 40 30 20 10 0 -10
f1 (ppm)

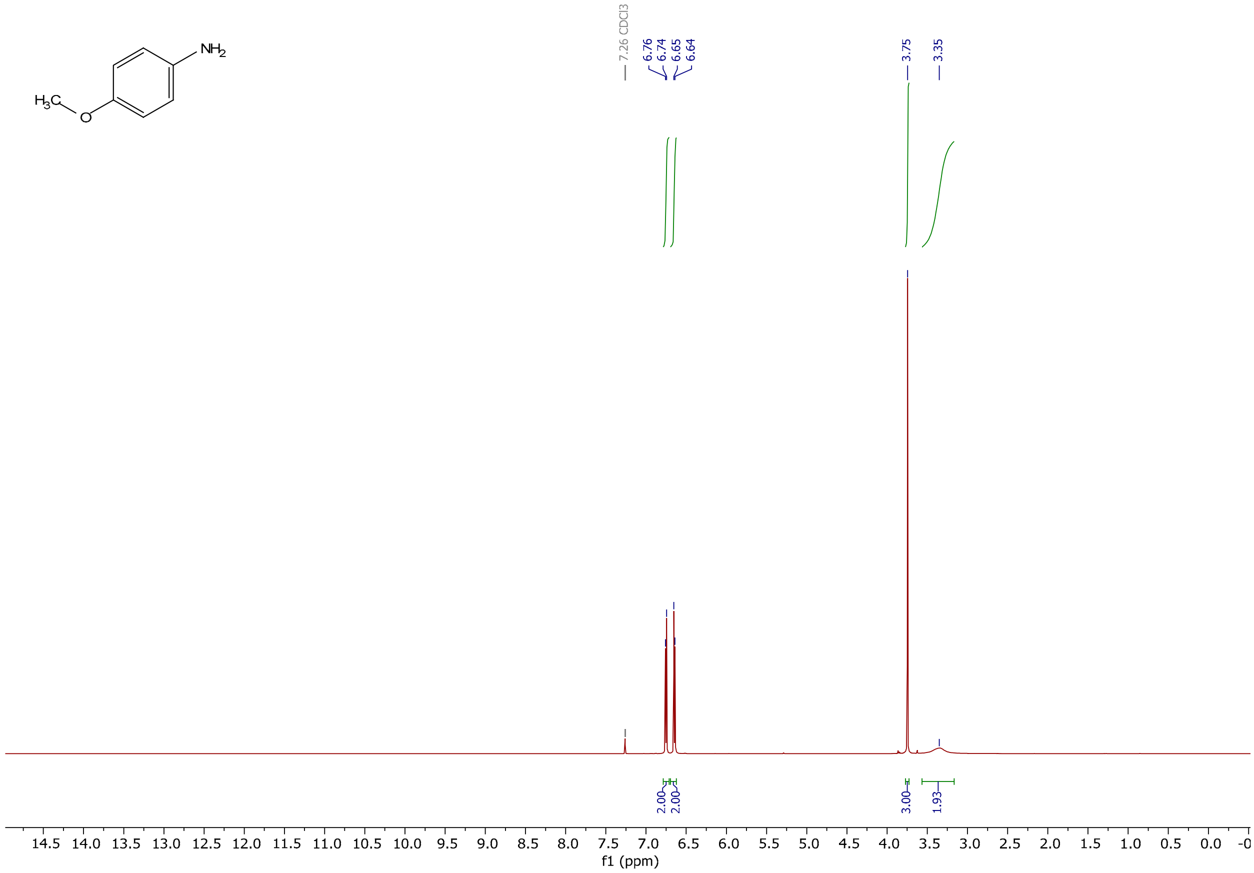
NH2
H3C
O
7.26 CDCl3
6.76
6.74
6.65
6.64
3.75
3.35
2.00
2.00
3.00
1.93
f1 (ppm)

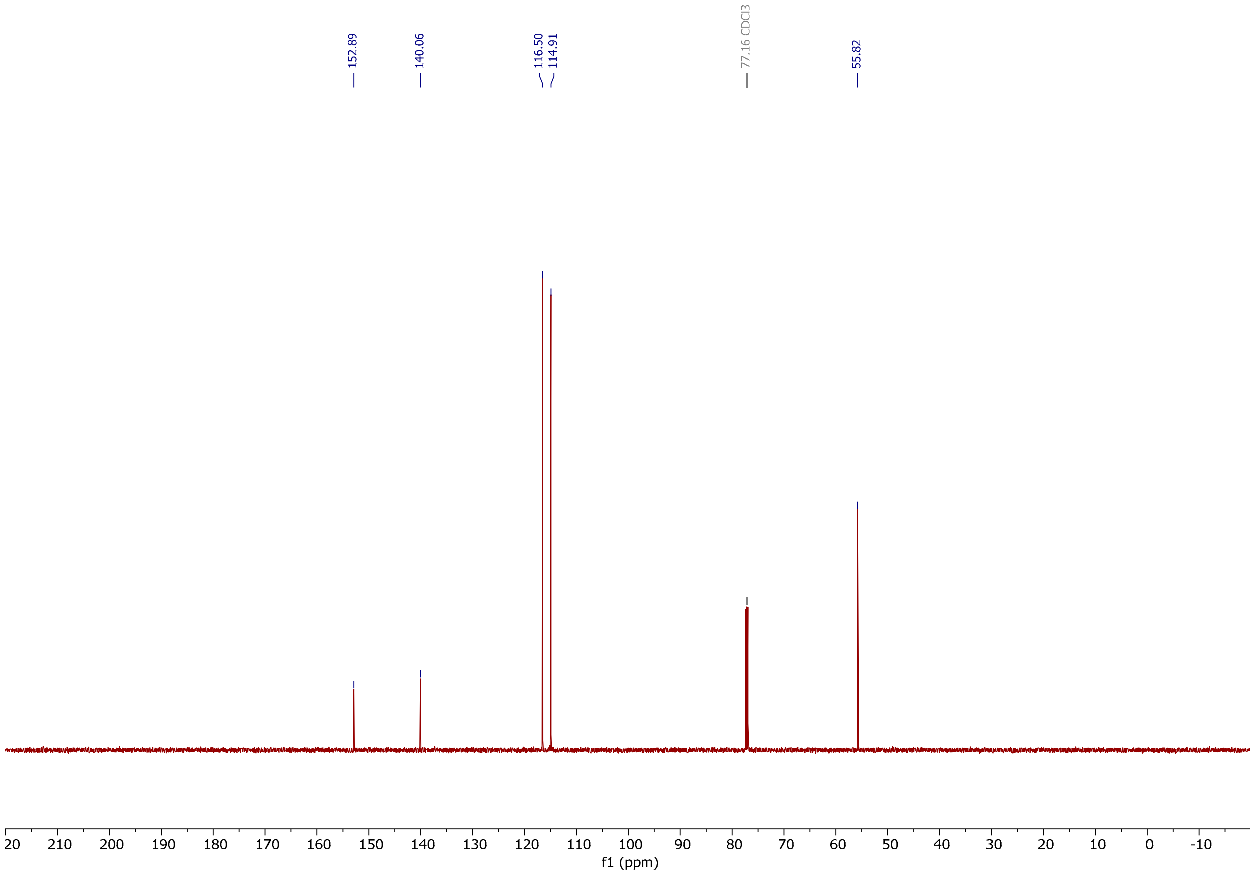
152.89
140.06
116.50
114.91
77.16 CDCl3
55.82
f1 (ppm)

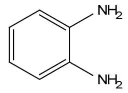
NH2
NH2

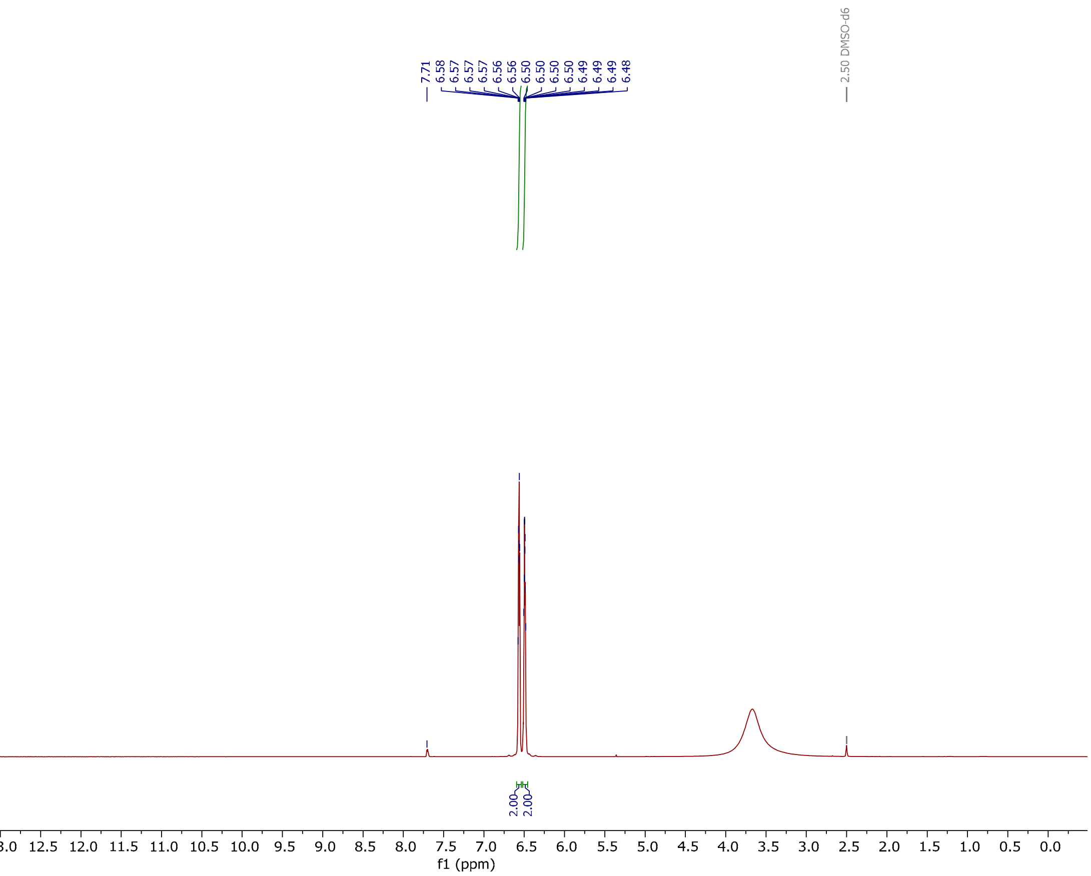
7.71
6.58
6.57
6.57
6.56
6.56
6.50
6.50
6.50
6.50
6.49
6.49
6.49
6.48
2.50 DMSO-d6
2.00
2.00
14.5 14.0 13.5 13.0 12.5 12.0 11.5 11.0 10.5 10.0 9.5 9.0 8.5 8.0 7.5 7.0 6.5 6.0 5.5 5.0 4.5 4.0 3.5 3.0 2.5 2.0 1.5 1.0 0.5 0.0
f1 (ppm)

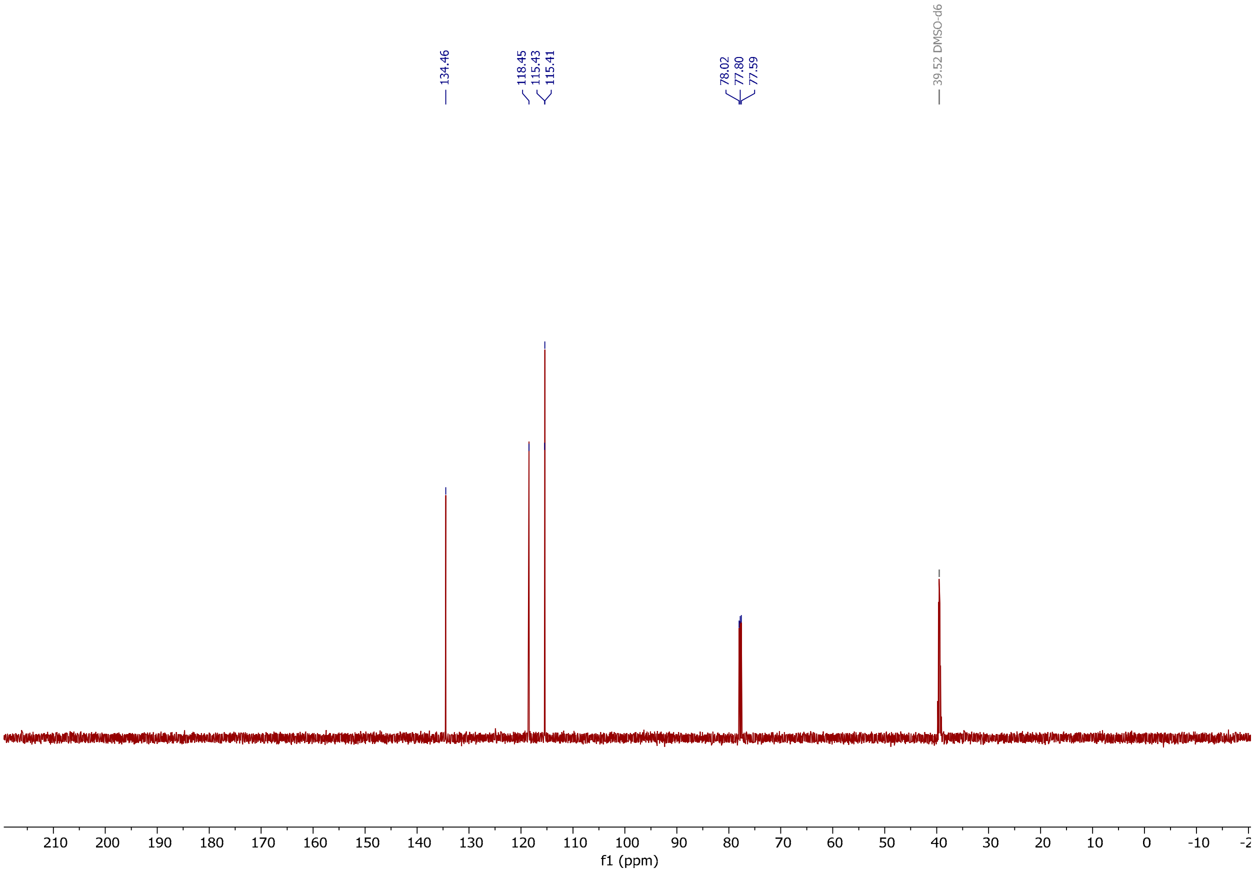
134.46
118.45
115.43
115.41
78.02
77.80
77.59
39.52 DMSO-d6
210 200 190 180 170 160 150 140 130 120 110 100 90 80 70 60 50 40 30 20 10 0 -10
f1 (ppm)

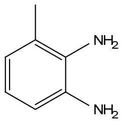
NH2
NH2

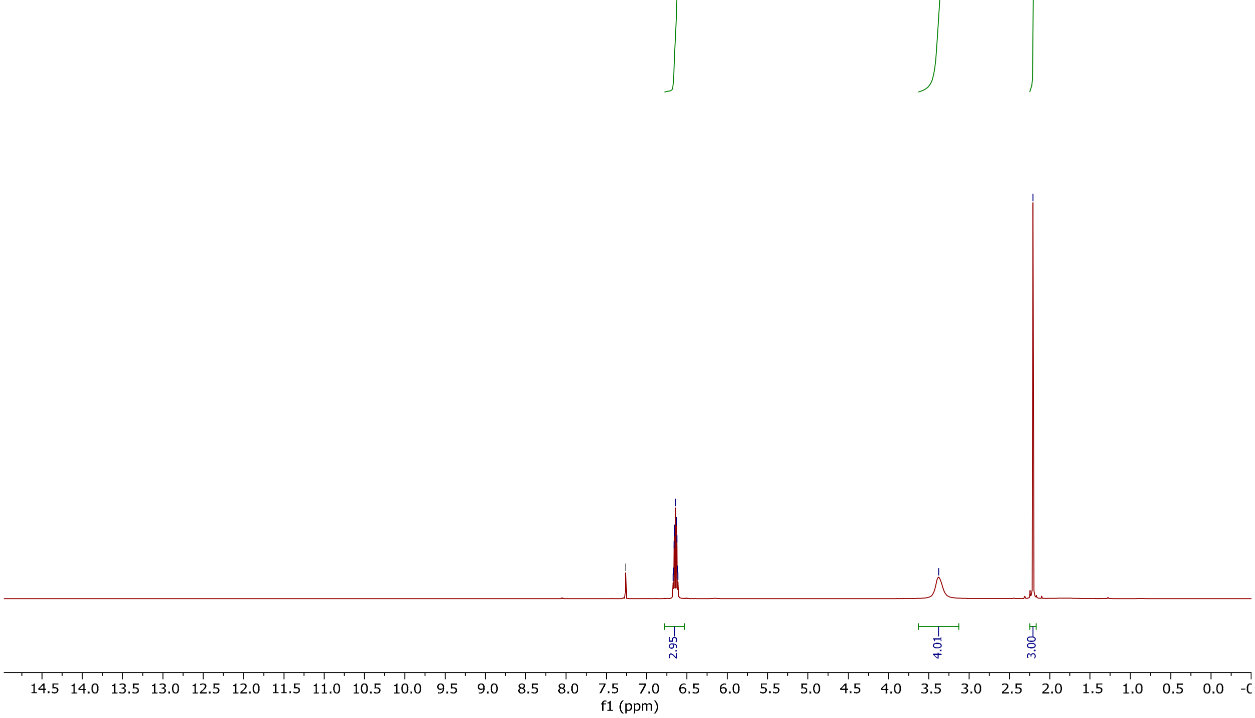
7.26 CDCl3
6.67
6.67
6.66
6.66
6.66
6.65
6.64
6.63
6.63
6.62
6.62
6.61
3.38
2.21
2.95
4.01
3.00
14.5 14.0 13.5 13.0 12.5 12.0 11.5 11.0 10.5 10.0 9.5 9.0 8.5 8.0 7.5 7.0 6.5 6.0 5.5 5.0 4.5 4.0 3.5 3.0 2.5 2.0 1.5 1.0 0.5 0.0
f1 (ppm)

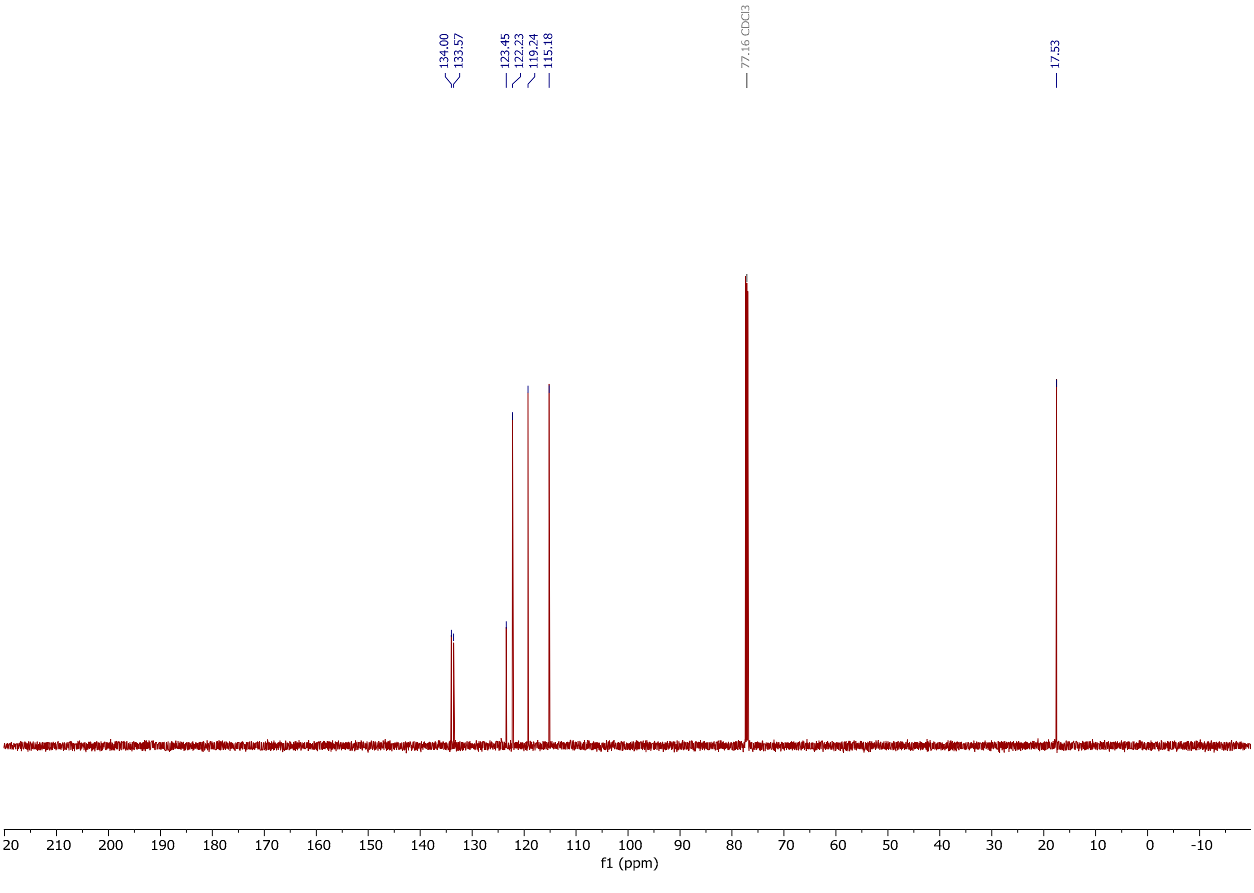
134.00
133.57
123.45
122.23
119.24
115.18
77.16 CDCl3
17.53
210 200 190 180 170 160 150 140 130 120 110 100 90 80 70 60 50 40 30 20 10 0 -10
f1 (ppm)

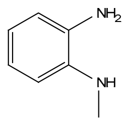
NH2
NH

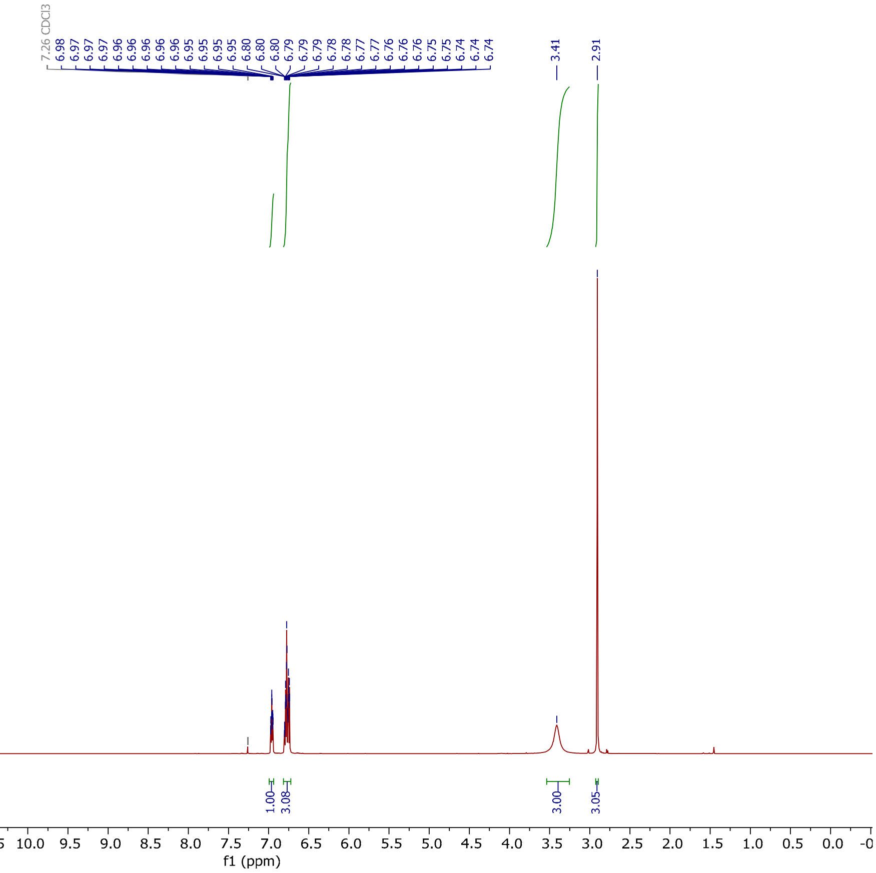
7.26 CDCl3
6.98
6.97
6.97
6.97
6.96
6.96
6.96
6.96
6.96
6.95
6.95
6.95
6.95
6.80
6.80
6.80
6.79
6.79
6.79
6.79
6.78
6.78
6.77
6.77
6.76
6.76
6.76
6.75
6.75
6.74
6.74
6.74
3.41
2.91
1.00
3.08
3.00
3.05
14.5 14.0 13.5 13.0 12.5 12.0 11.5 11.0 10.5 10.0 9.5 9.0 8.5 8.0 7.5 7.0 6.5 6.0 5.5 5.0 4.5 4.0 3.5 3.0 2.5 2.0 1.5 1.0 0.5 0.0
f1 (ppm)

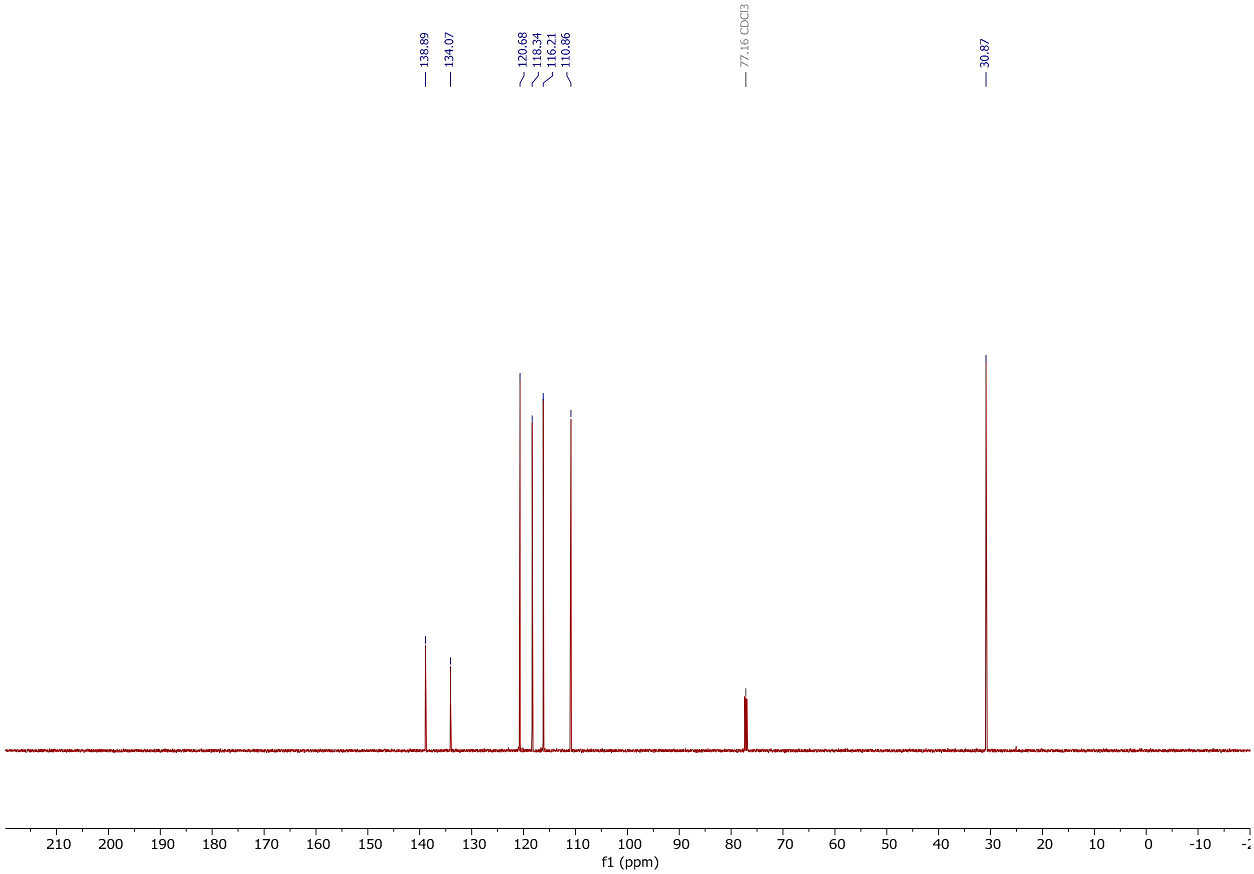
138.89
134.07
120.68
118.34
116.21
110.86
77.16 CDCl3
30.87
210 200 190 180 170 160 150 140 130 120 110 100 90 80 70 60 50 40 30 20 10 0 -10
f1 (ppm)

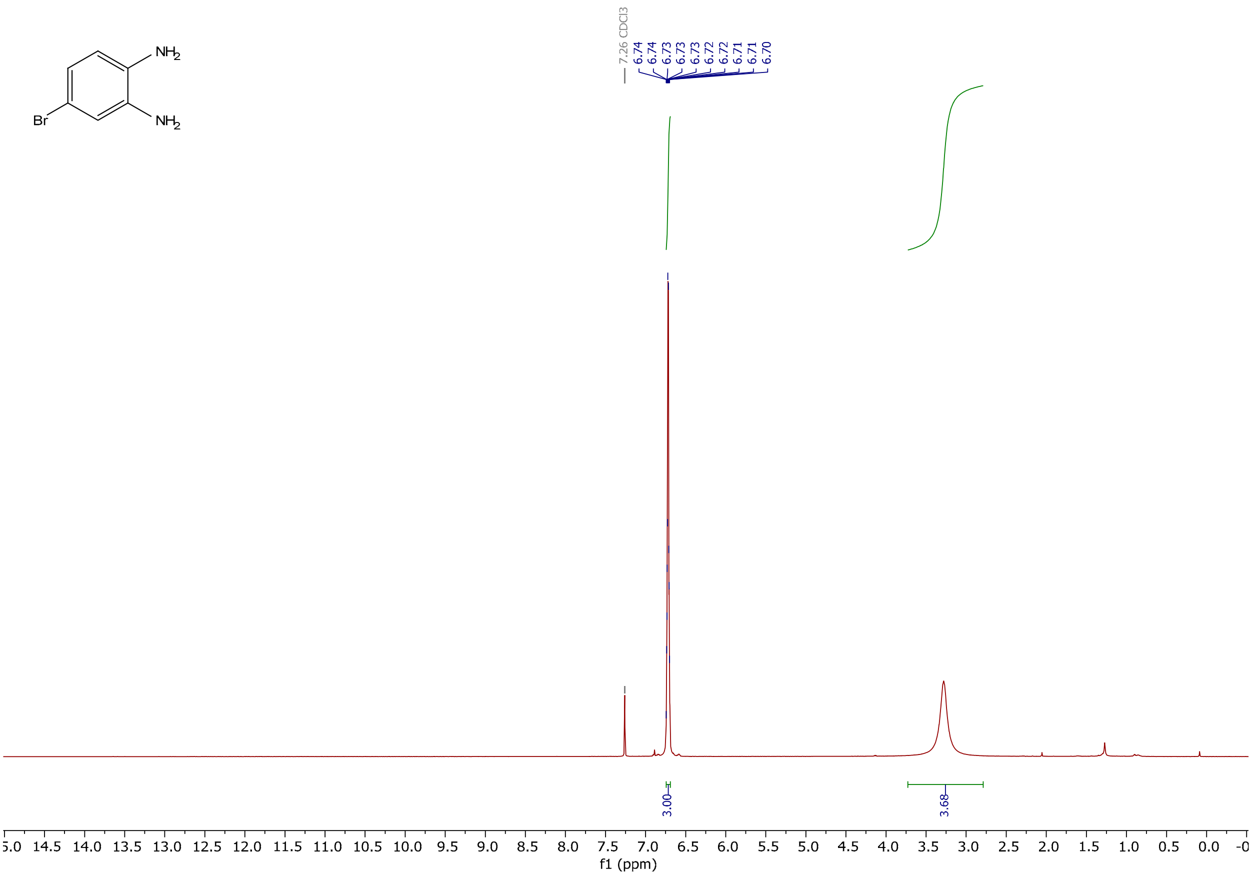
NH2
NH2
Br
7.26 CDCl3
6.74
6.74
6.73
6.73
6.73
6.72
6.72
6.71
6.71
6.70
3.00
3.68
f1 (ppm)

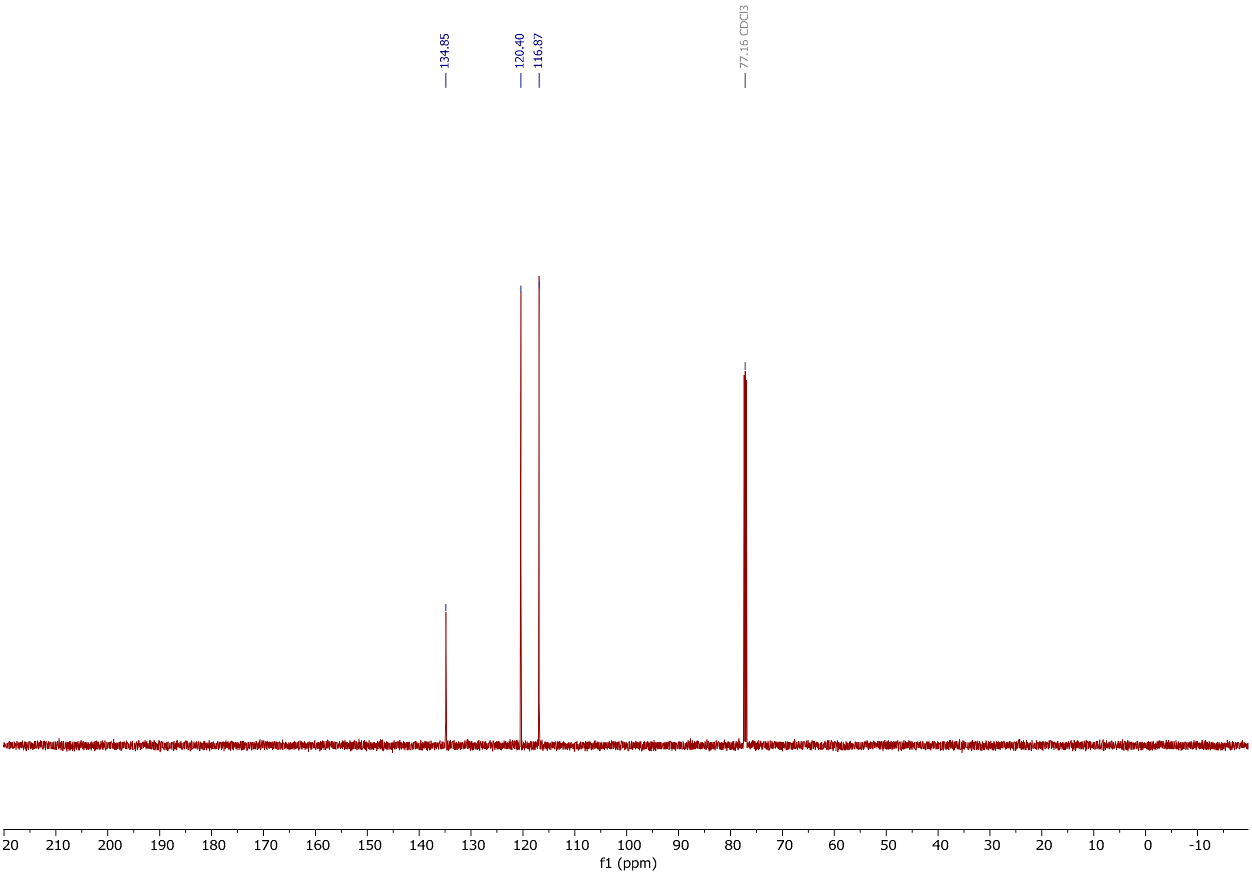
134.85
120.40
116.87
77.16 CDCl3
f1 (ppm)

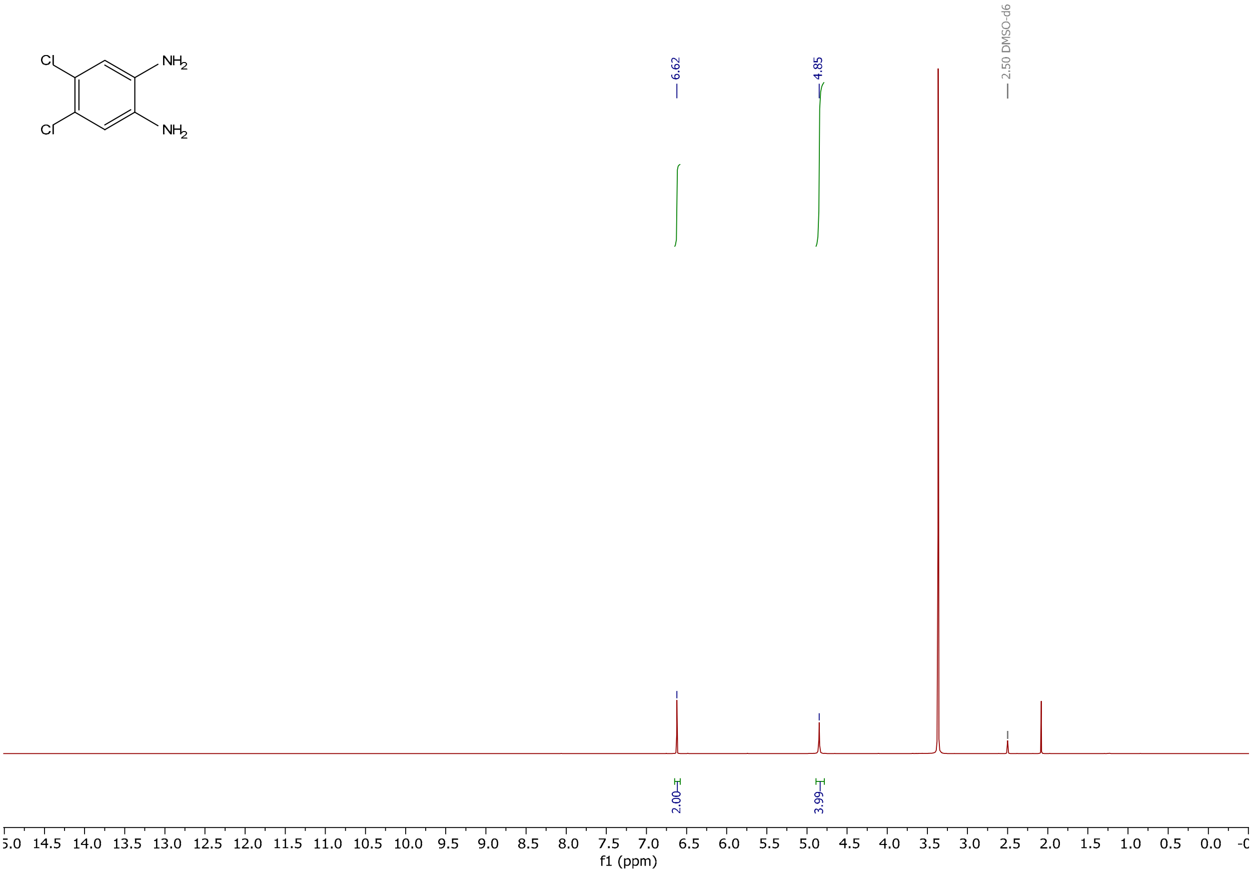
Cl
Cl
NH2
NH2
6.62
4.85
2.50 DMSO-d6
2.00
3.99
f1 (ppm)

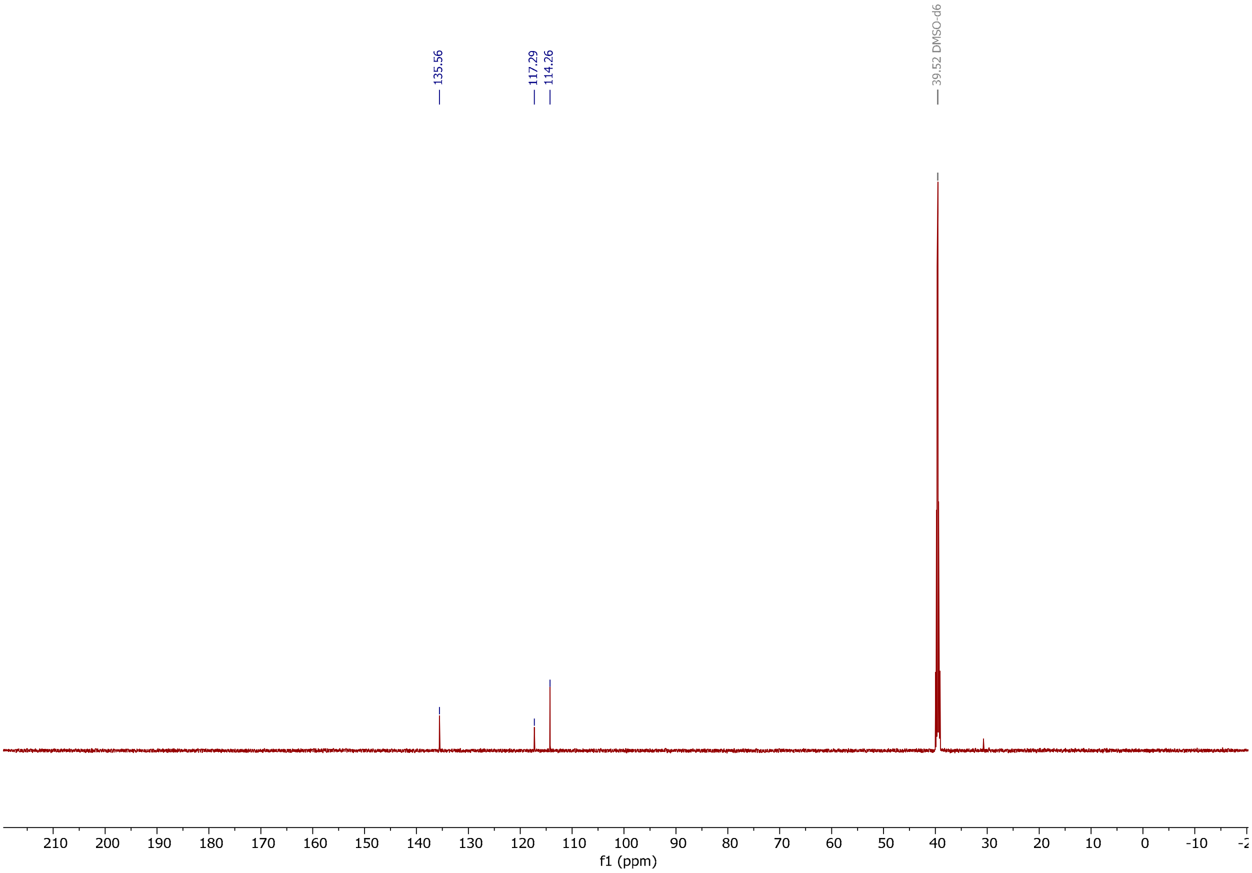
135.56
117.29
114.26
39.52 DMSO-d6
f1 (ppm)

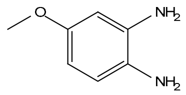
O
NH2
NH2

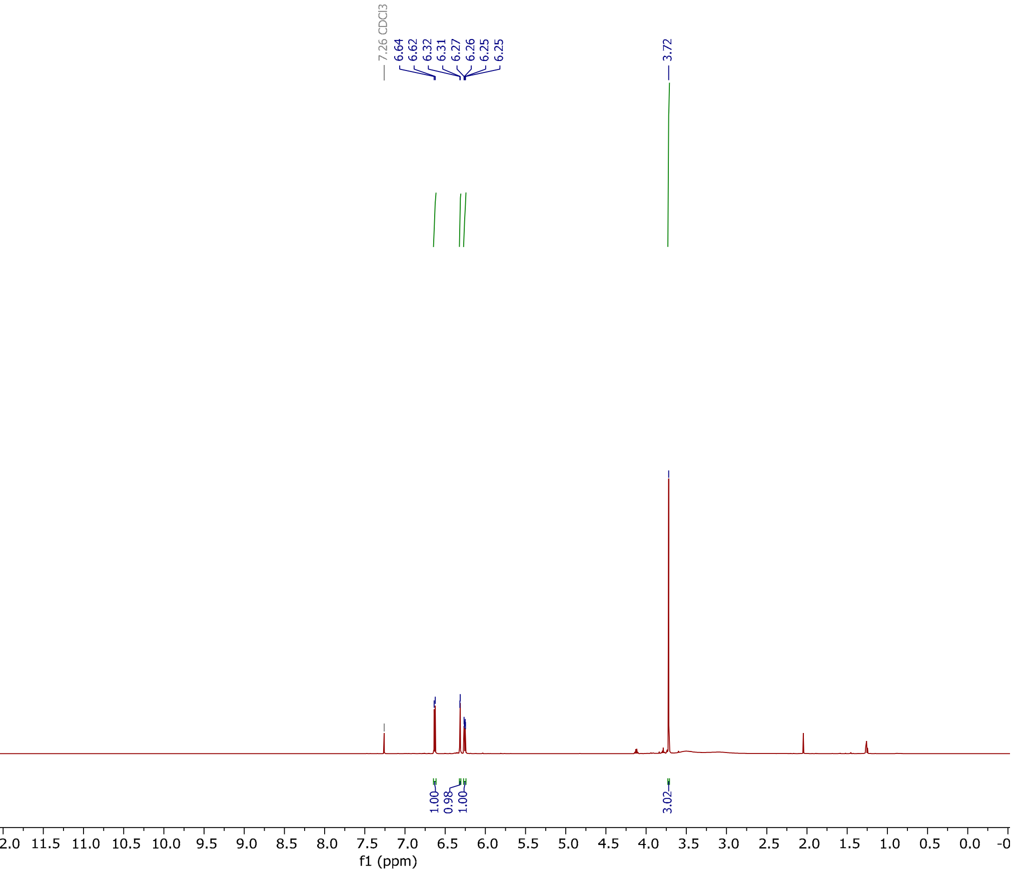
7.26 CDCl3
6.64
6.62
6.32
6.31
6.27
6.26
6.25
6.25
3.72
1.00
0.98
1.00
3.02
f1 (ppm)

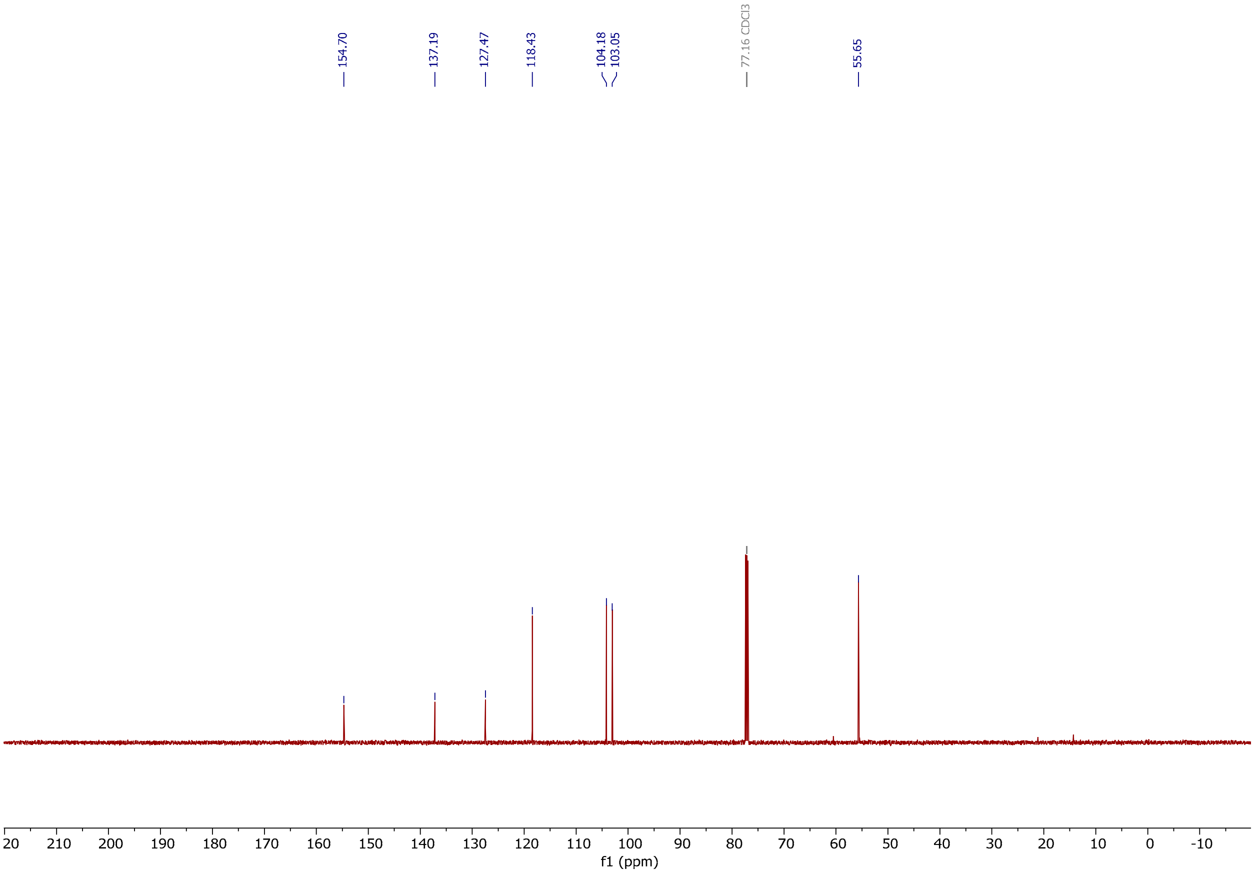
154.70
137.19
127.47
118.43
104.18
103.05
77.16 CDCl3
55.65
f1 (ppm)

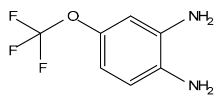

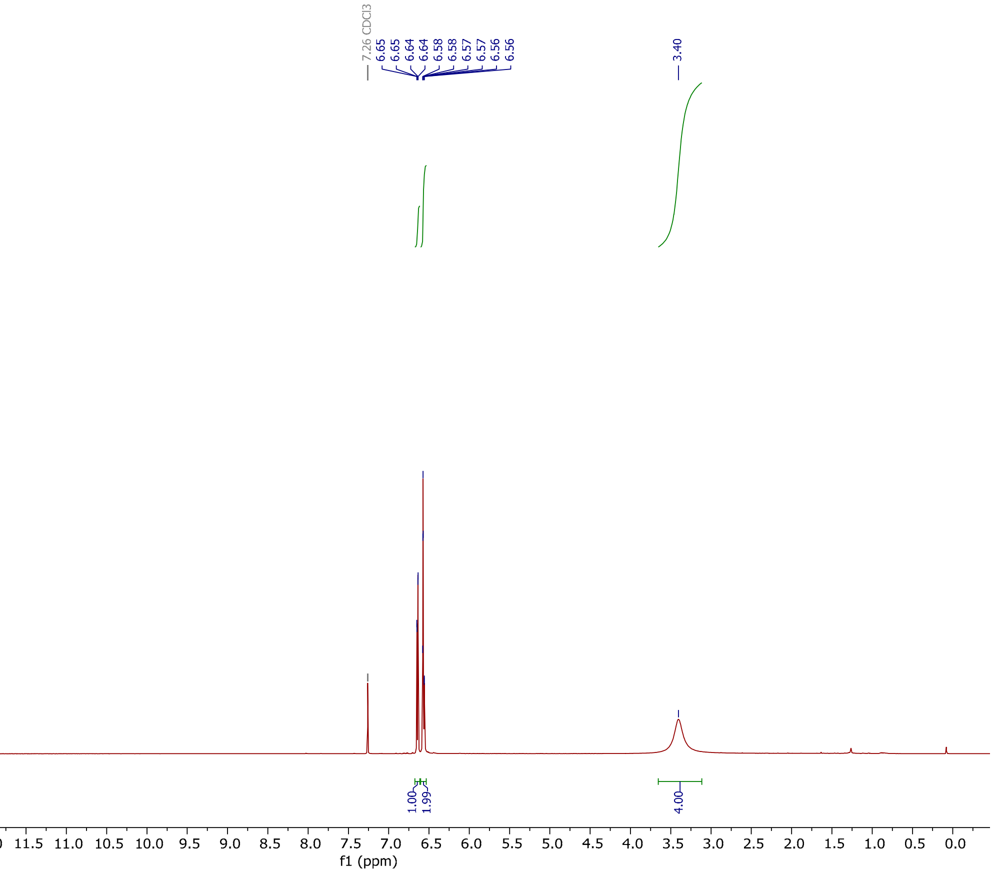
7.26 CDCl3
6.65
6.65
6.64
6.64
6.58
6.58
6.57
6.57
6.56
6.56
3.40
1.00
1.99
4.00
f1 (ppm)

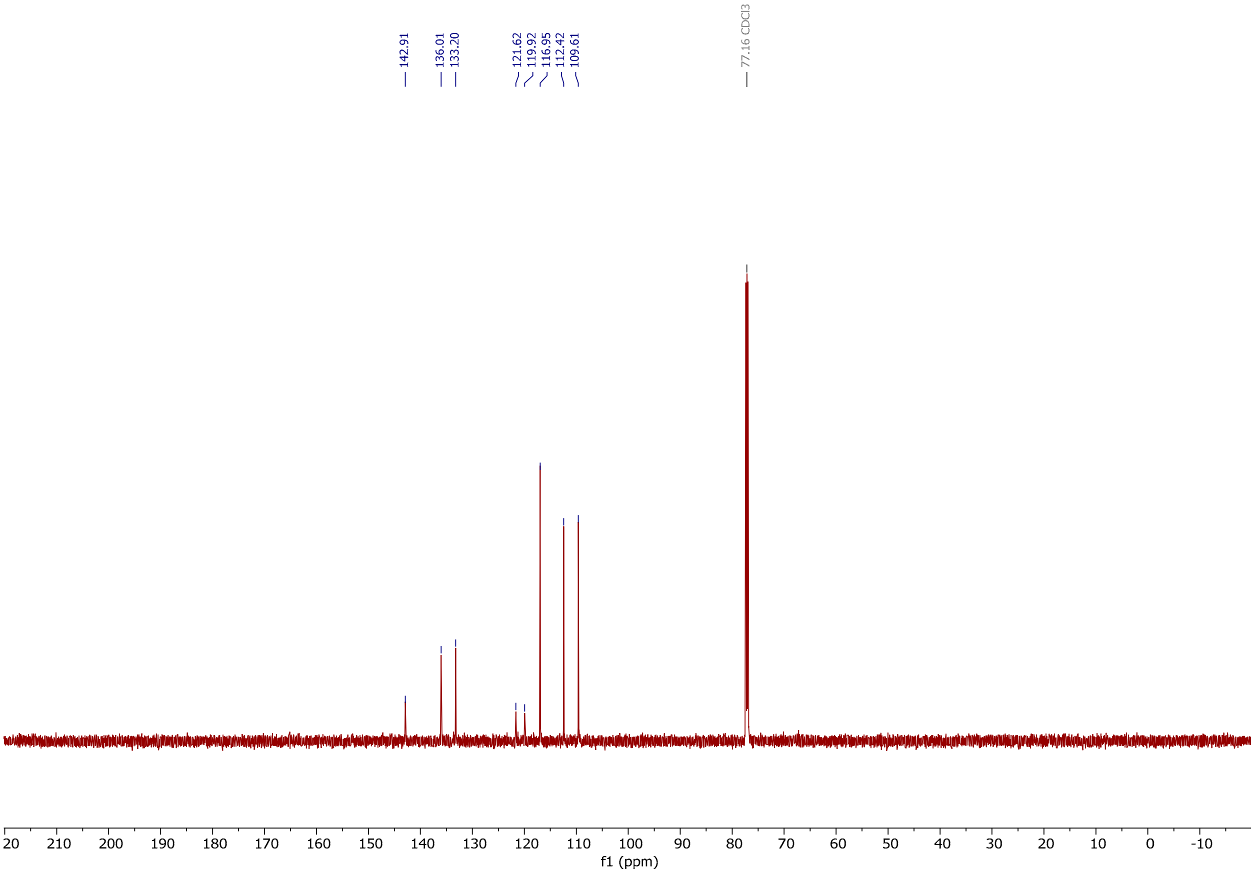
142.91
136.01
133.20
121.62
119.92
116.95
112.42
109.61
77.16 CDCl3
f1 (ppm)

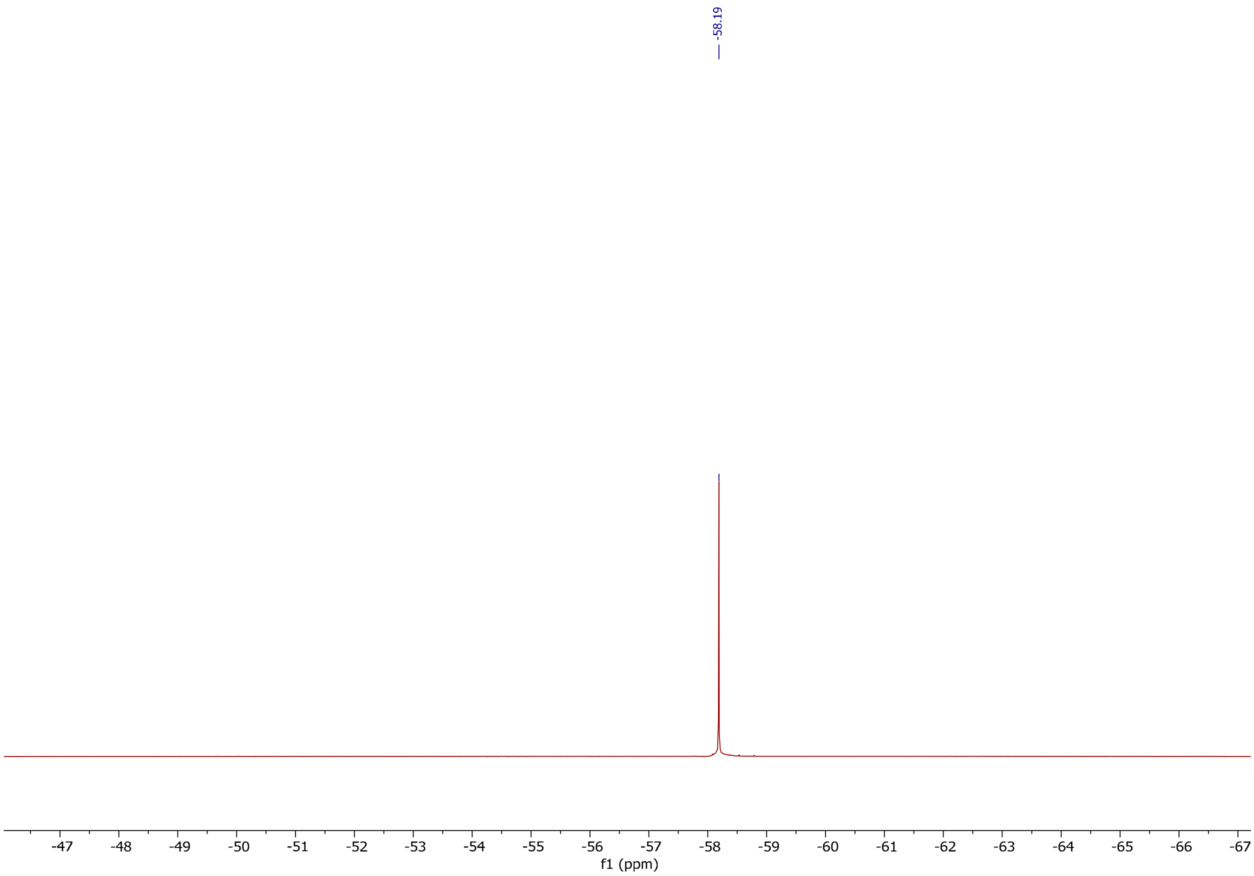

-58.19
-47
-48
-49
-50
-51
-52
-53
-54
-55
-56
-57
-58
-59
-60
-61
-62
-63
-64
-65
-66
-67
f1 (ppm)

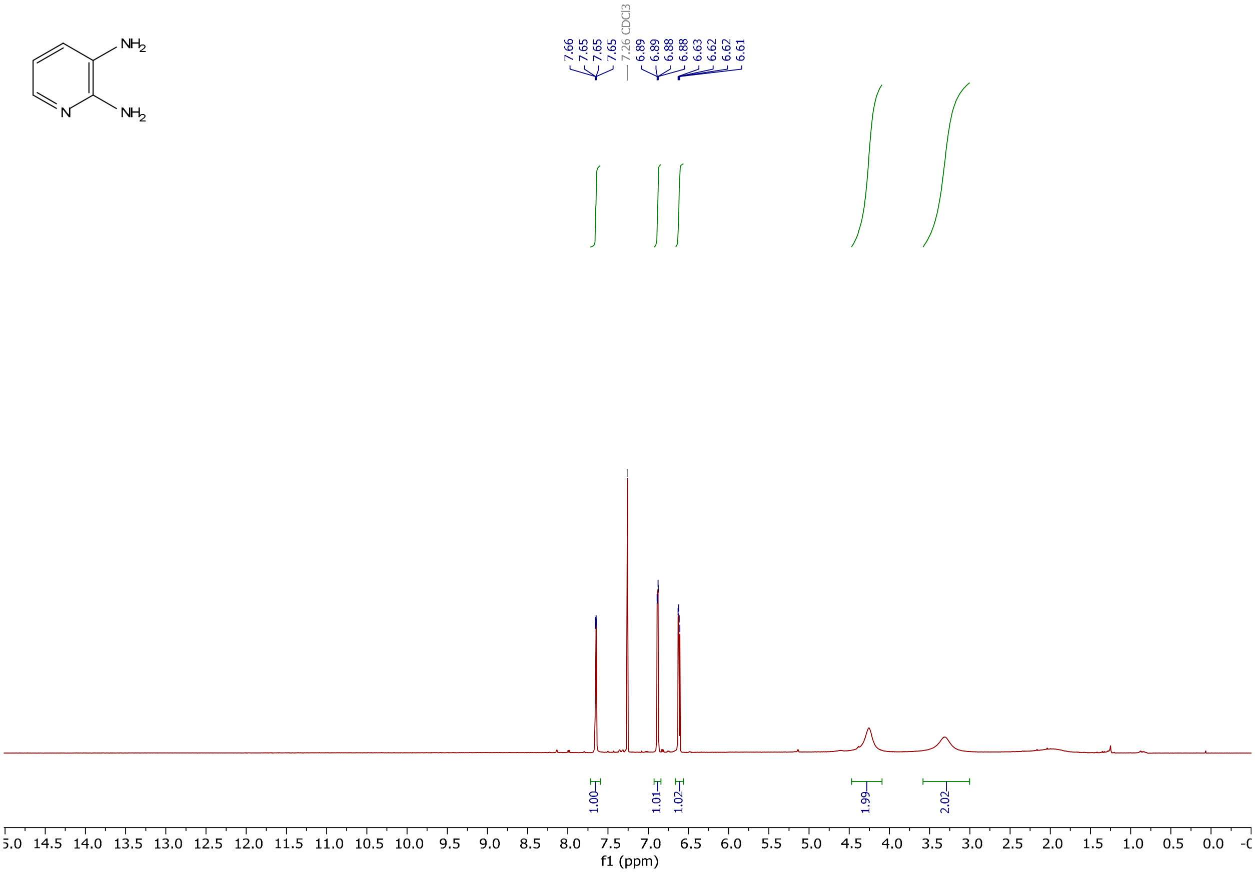
NH2
N
NH2
7.66
7.65
7.65
7.65
7.26 CDCl3
6.89
6.89
6.88
6.88
6.63
6.62
6.62
6.61
1.00
1.01
1.02
1.99
2.02
f1 (ppm)

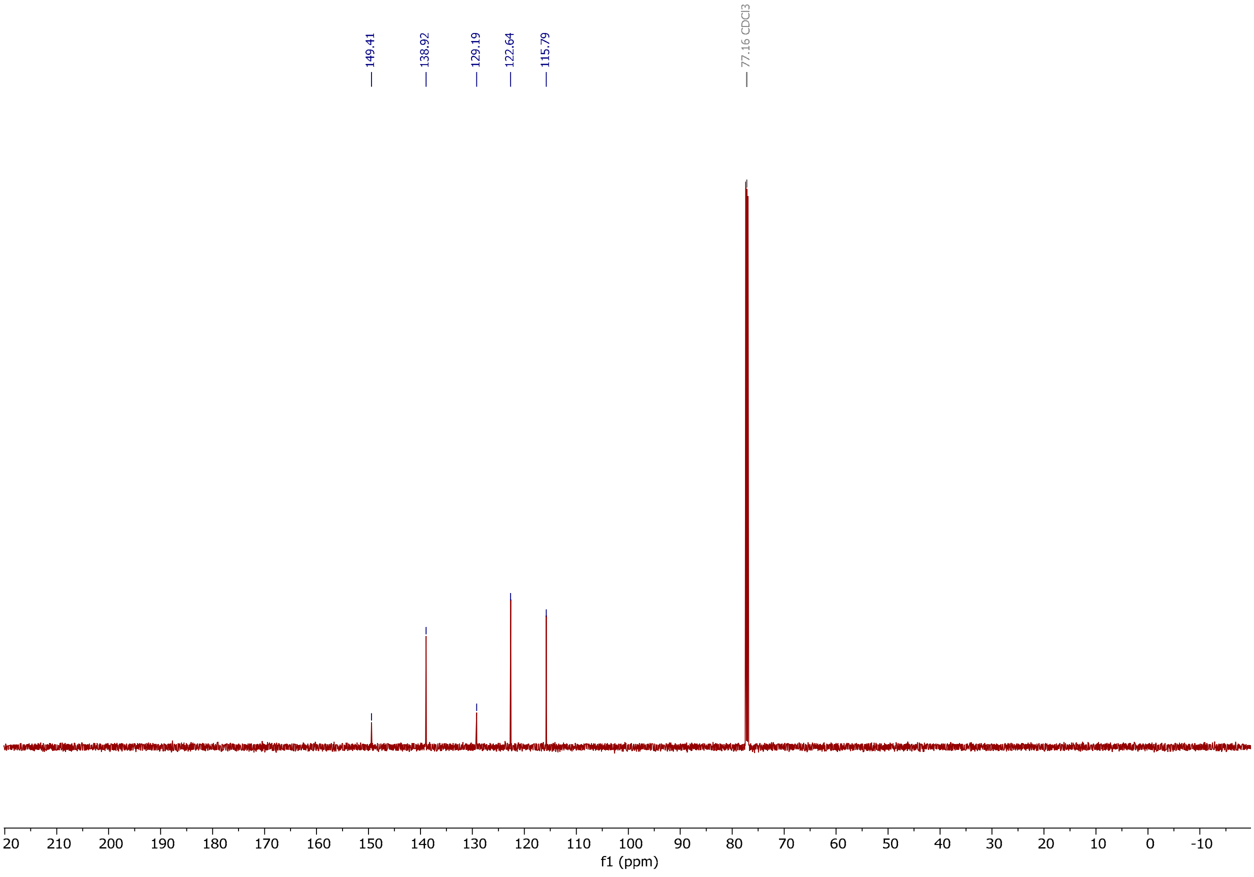
149.41
138.92
129.19
122.64
115.79
77.16 CDCl3
f1 (ppm)

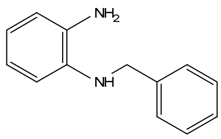
NH2
NH

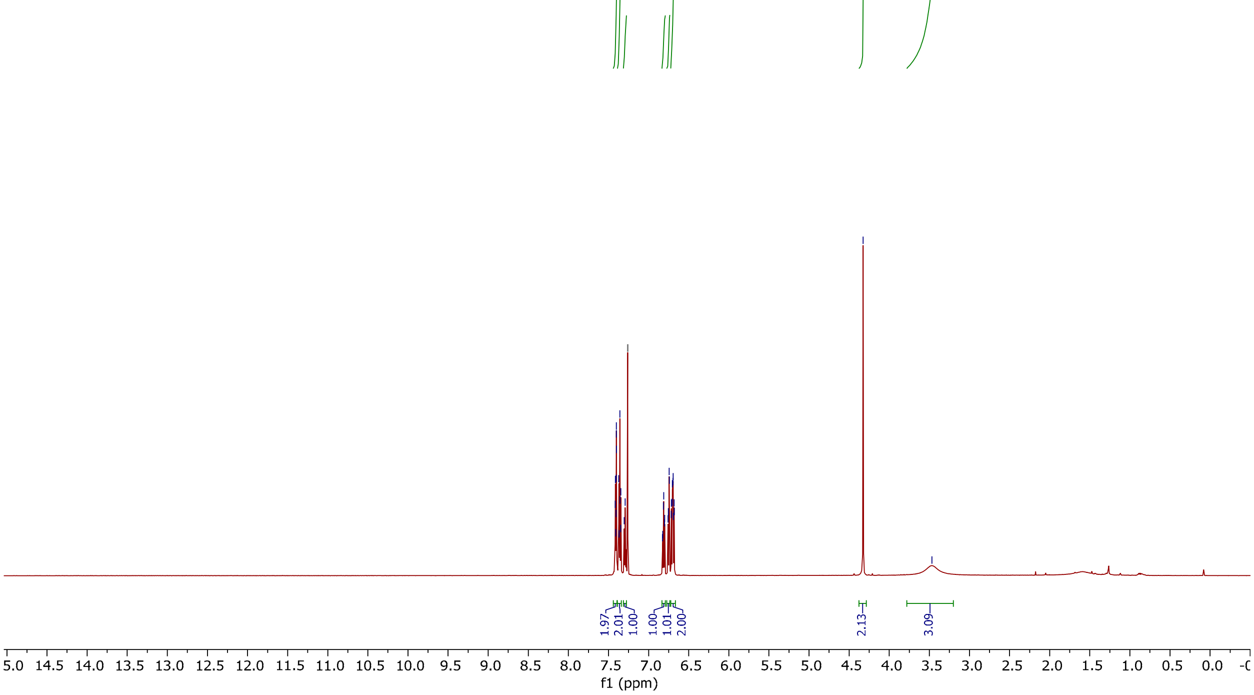
7.42 7.41 7.41 7.41 7.40 7.40 7.40 7.40 7.37 7.37 7.36 7.35 7.35 7.30 7.29 7.26 CDCl3 6.83 6.82 6.81 6.81 6.80 6.80 6.76 6.75 6.74 6.74 6.72 6.72 6.71 6.70 6.70 6.69 6.69 6.68 4.33 3.47
1.97 2.01 1.00 1.00 1.01 2.00 2.13 3.09
f1 (ppm)

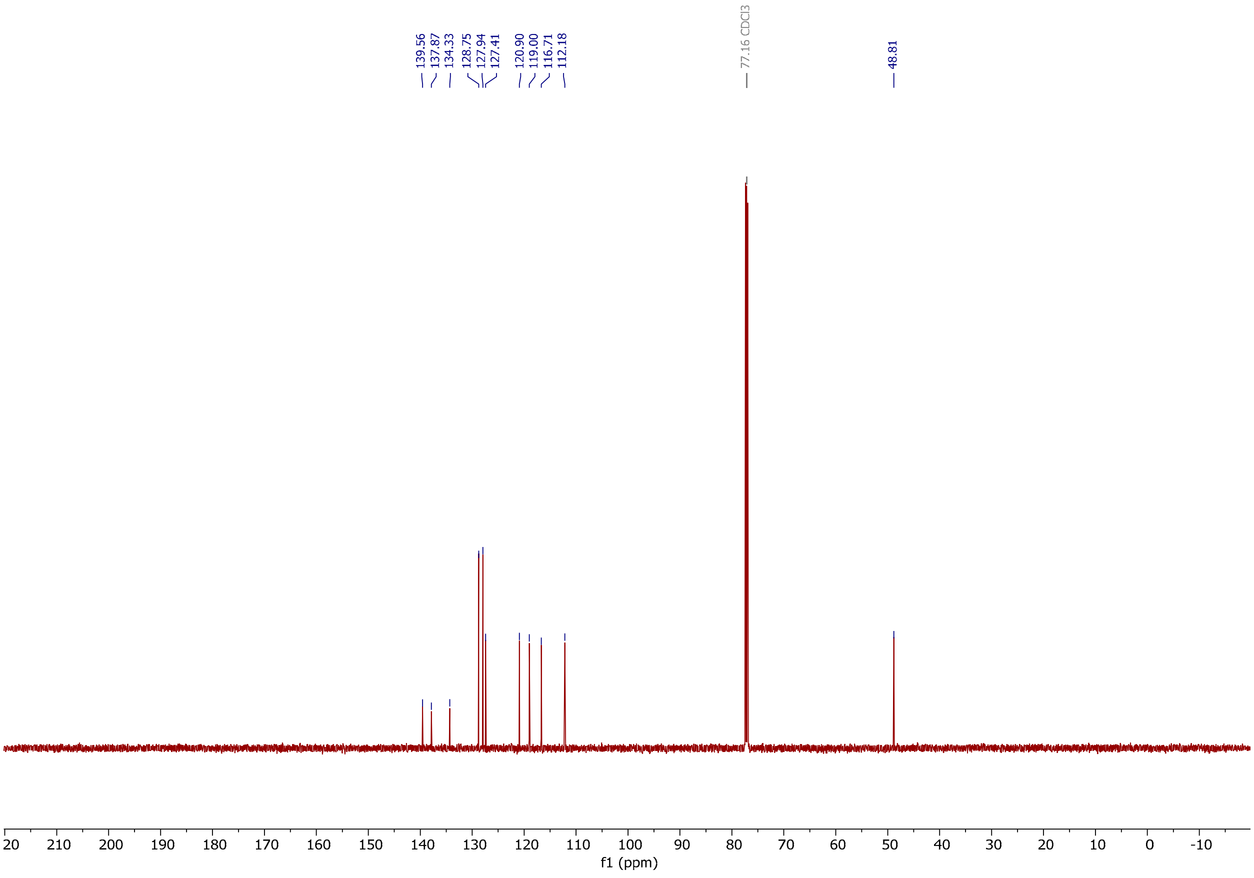
139.56 137.87 134.33 128.75 127.94 127.41 120.90 119.00 116.71 112.18
77.16 CDCl3
48.81
f1 (ppm)

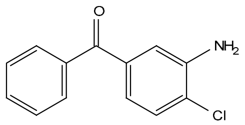
O
NH2
Cl

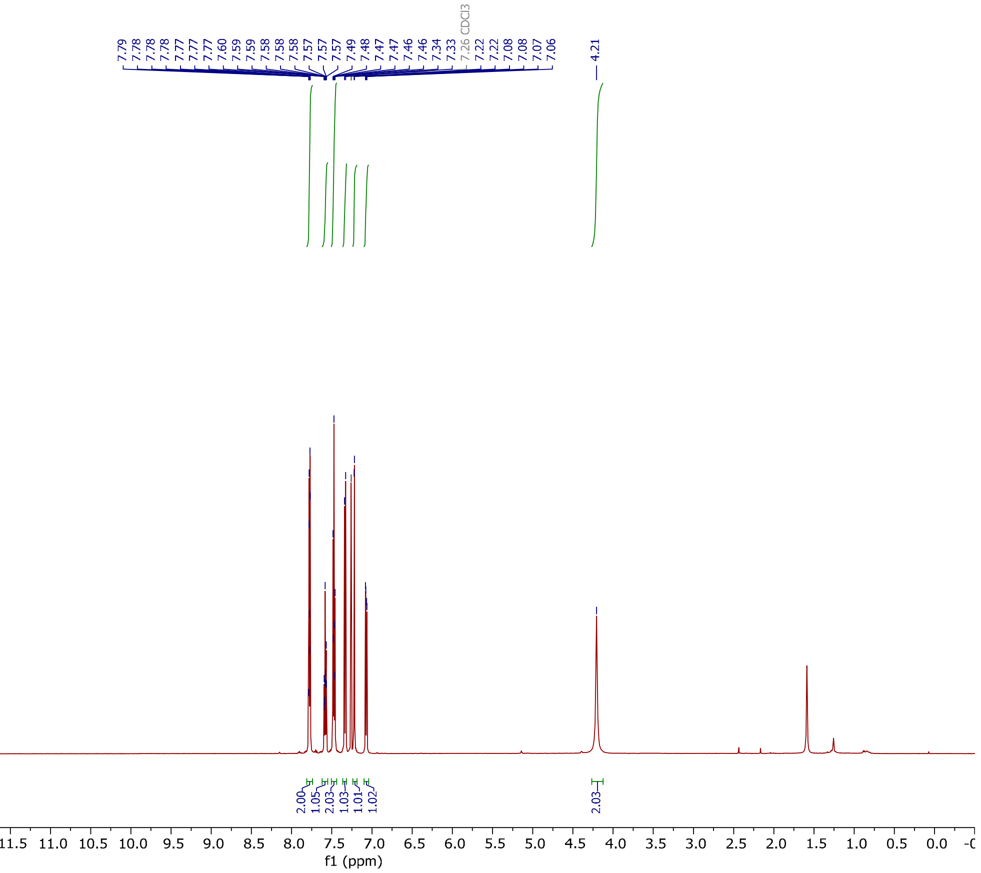
7.79
7.78
7.78
7.78
7.77
7.77
7.77
7.60
7.59
7.59
7.58
7.58
7.58
7.57
7.57
7.57
7.49
7.48
7.47
7.47
7.46
7.46
7.34
7.33
7.26 CDCl3
7.22
7.22
7.08
7.08
7.07
7.06
4.21
2.00
1.05
2.03
1.03
1.01
1.02
2.03
f1 (ppm)

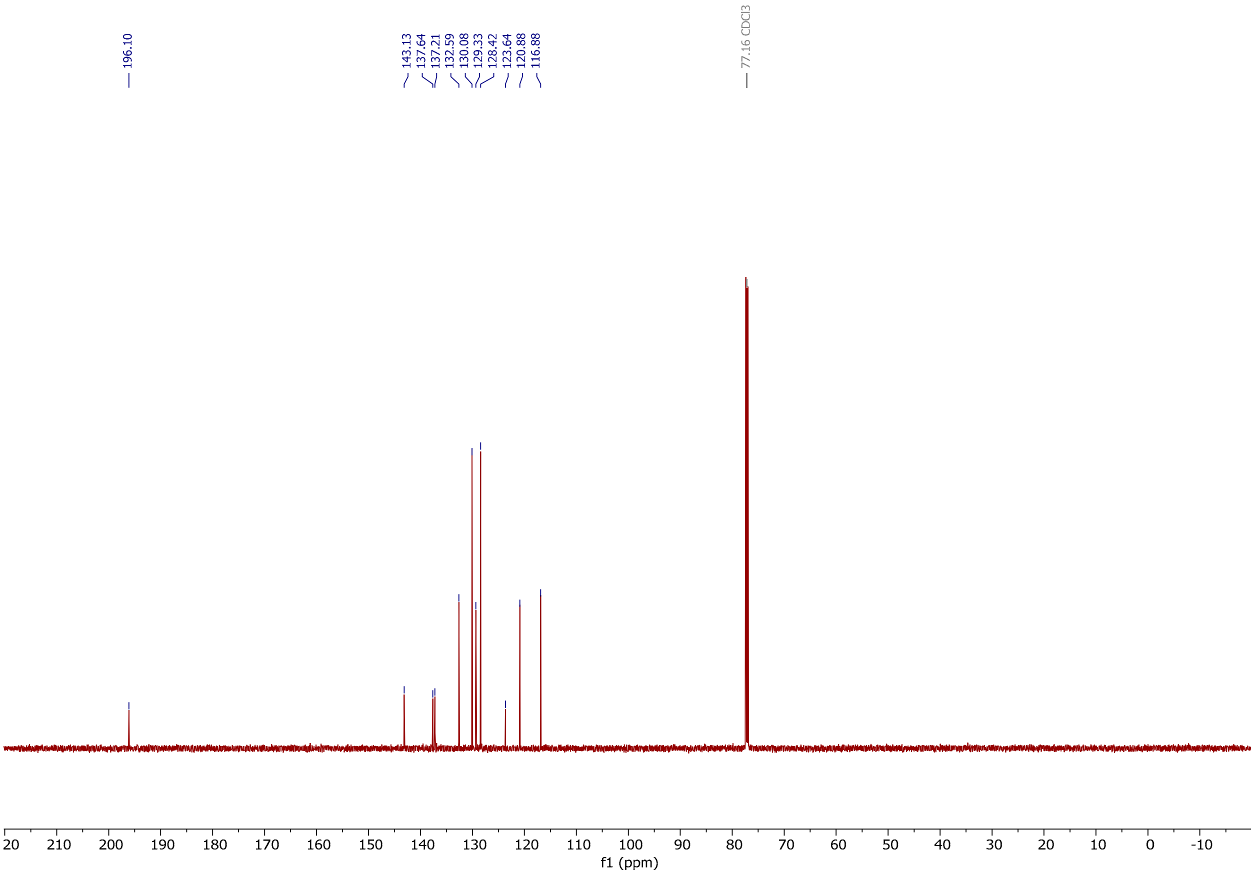
196.10
143.13
137.64
137.21
132.59
130.08
129.33
128.42
123.64
120.88
116.88
77.16 CDCl3
f1 (ppm)

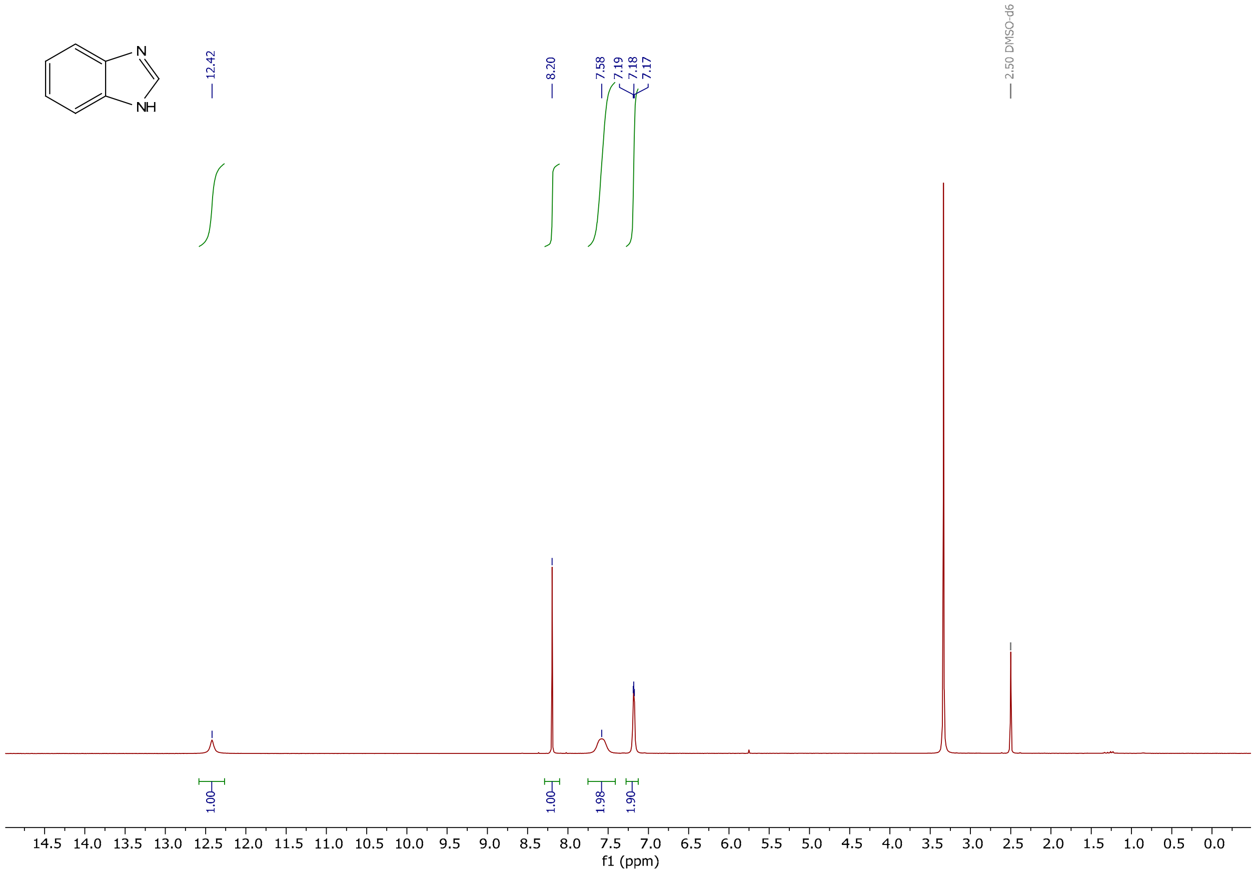
N
NH
12.42
8.20
7.58
7.19
7.18
7.17
2.50 DMSO-d6
1.00
1.00
1.98
1.90
14.5 14.0 13.5 13.0 12.5 12.0 11.5 11.0 10.5 10.0 9.5 9.0 8.5 8.0 7.5 7.0 6.5 6.0 5.5 5.0 4.5 4.0 3.5 3.0 2.5 2.0 1.5 1.0 0.5 0.0
f1 (ppm)

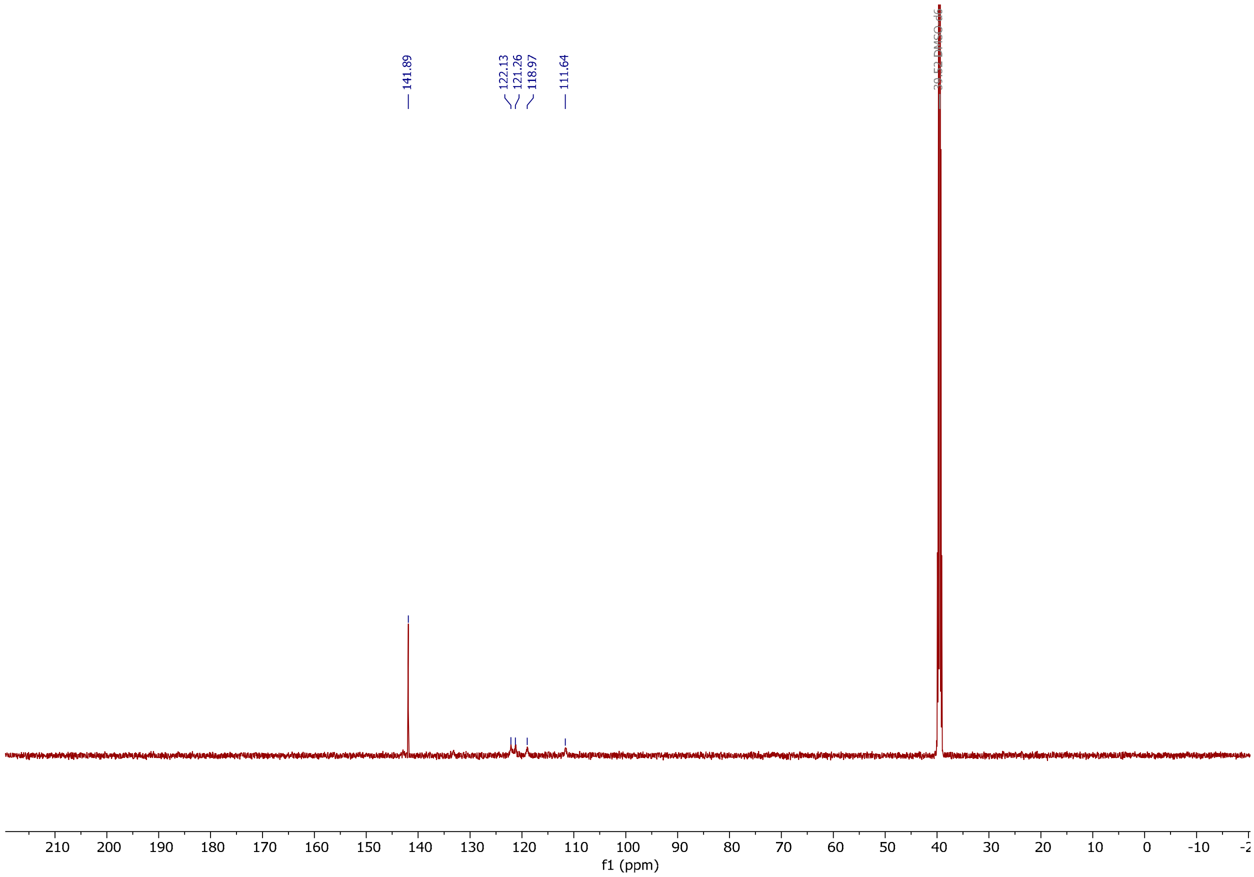
141.89
122.13
121.26
118.97
111.64
210 200 190 180 170 160 150 140 130 120 110 100 90 80 70 60 50 40 30 20 10 0 -10
f1 (ppm)

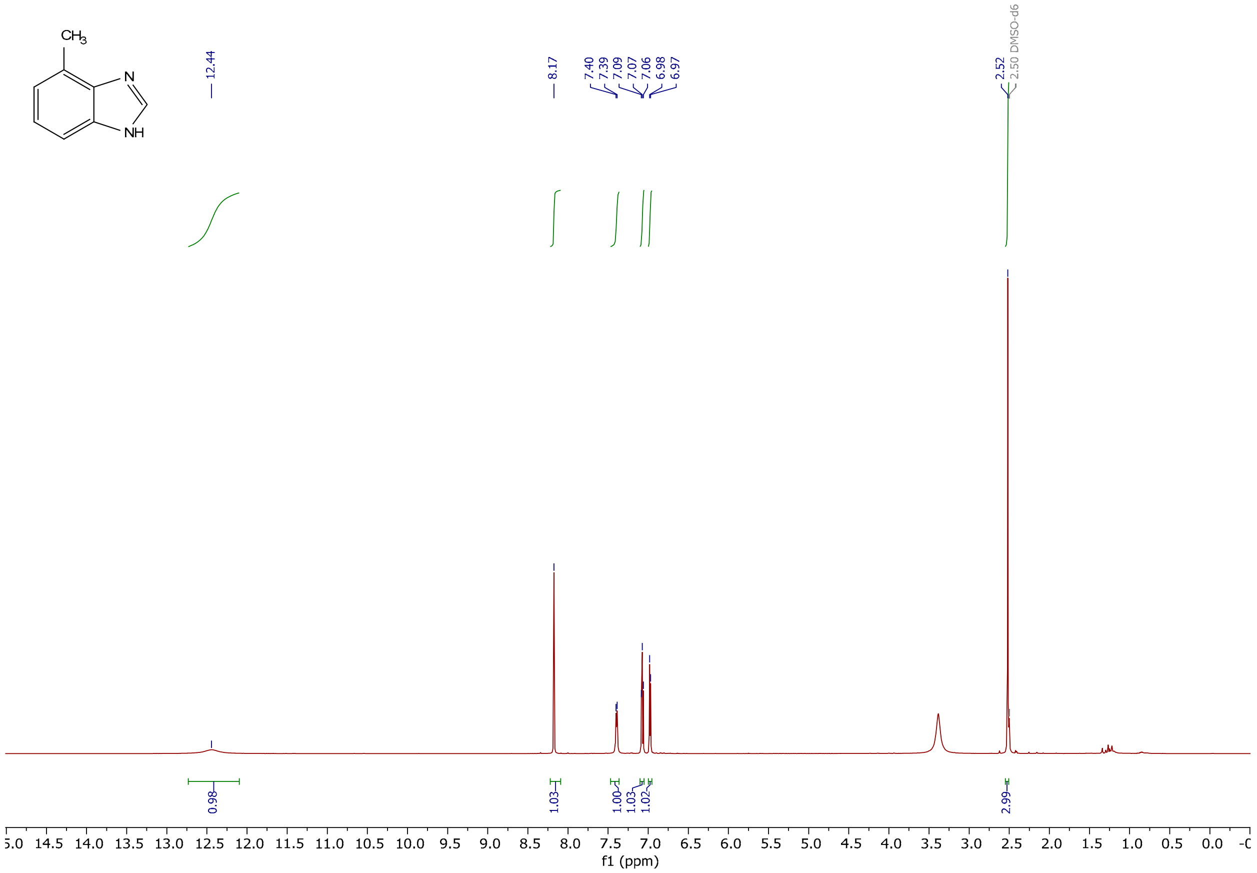
CH3
N
NH
12.44
8.17
7.40
7.39
7.09
7.07
7.06
6.98
6.97
2.52
2.50 DMSO-d6
0.98
1.03
1.00
1.03
1.02
2.99
f1 (ppm)

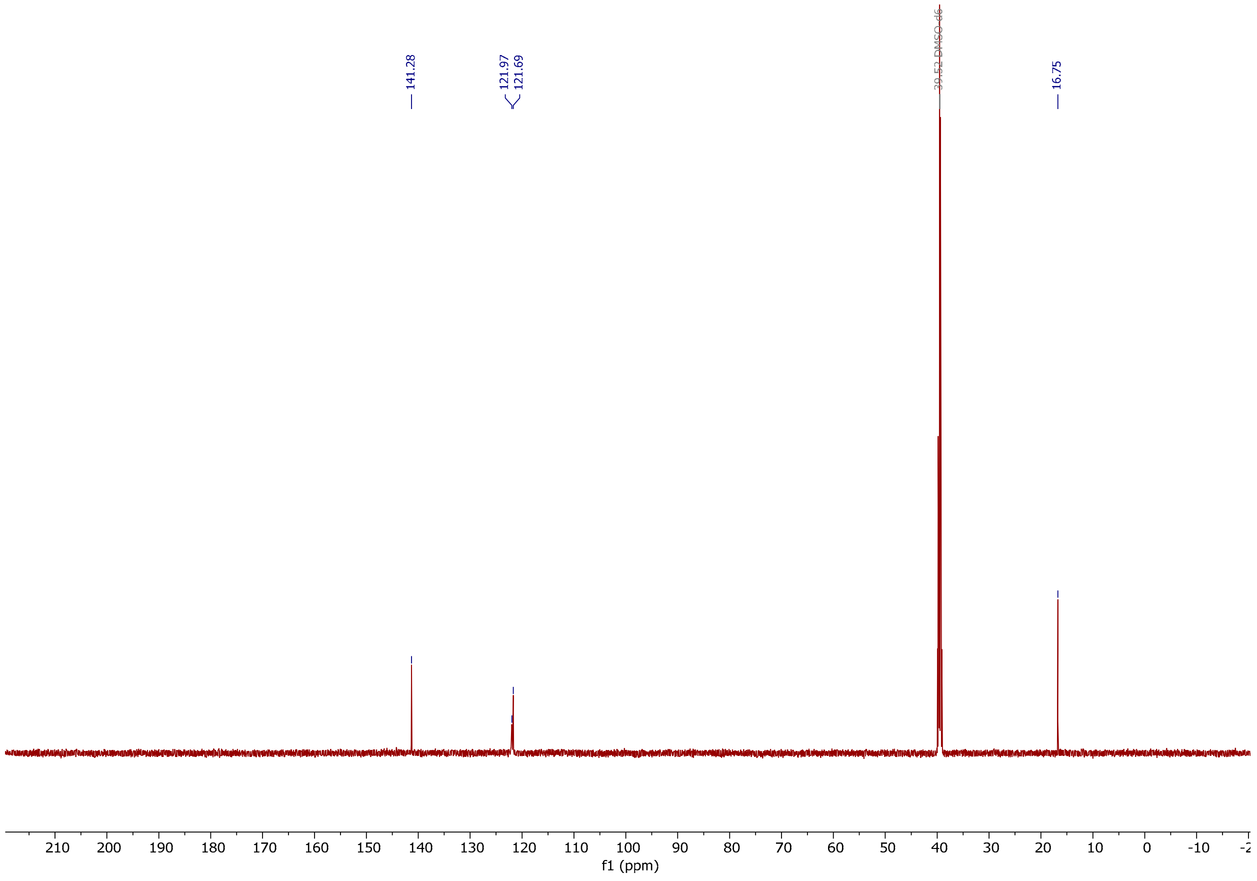
141.28
121.97
121.69
39.52 DMSO-d6
16.75
f1 (ppm)

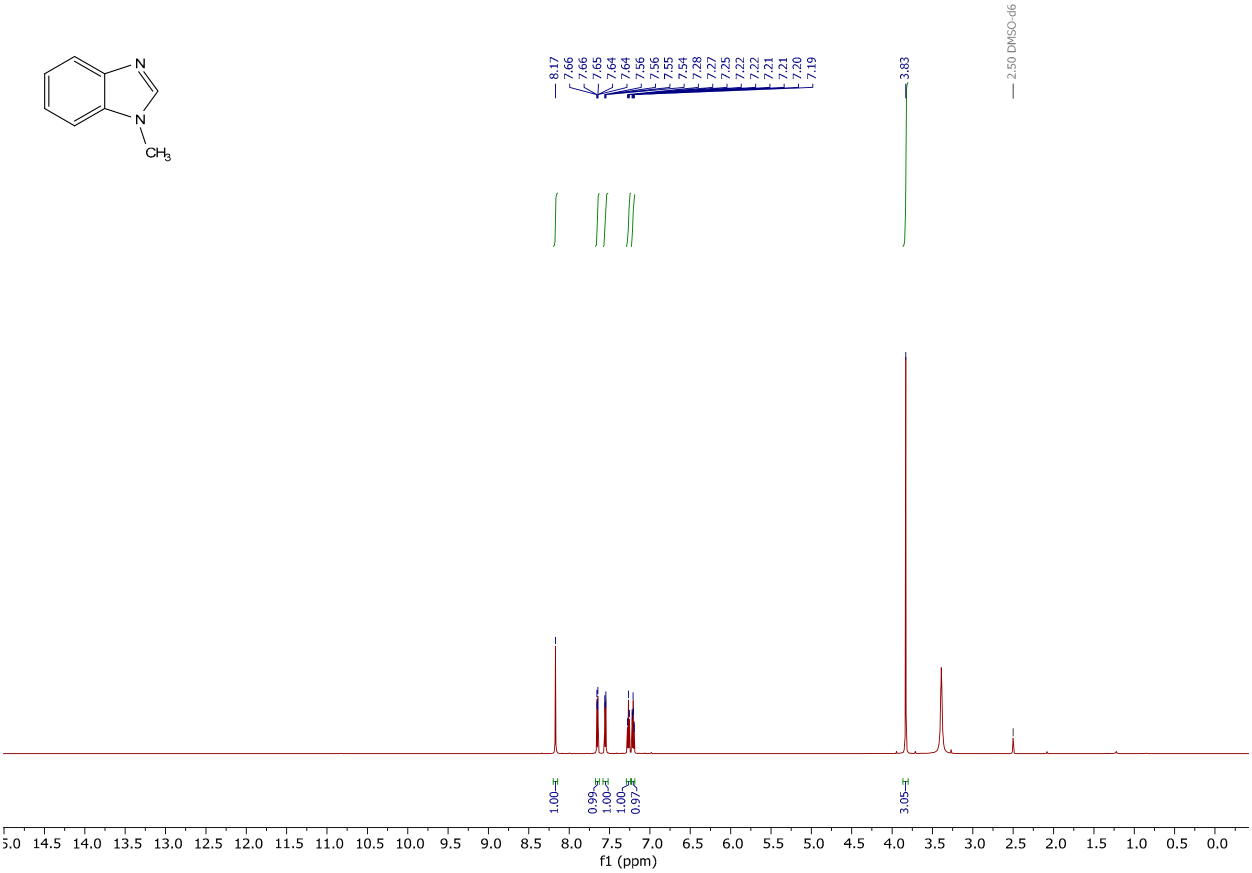
N
N
CH3
8.17
7.66
7.66
7.65
7.64
7.64
7.56
7.56
7.55
7.54
7.28
7.27
7.25
7.22
7.22
7.21
7.21
7.20
7.19
3.83
2.50 DMSO-d6
1.00
0.99
1.00
1.00
0.97
3.05
f1 (ppm)

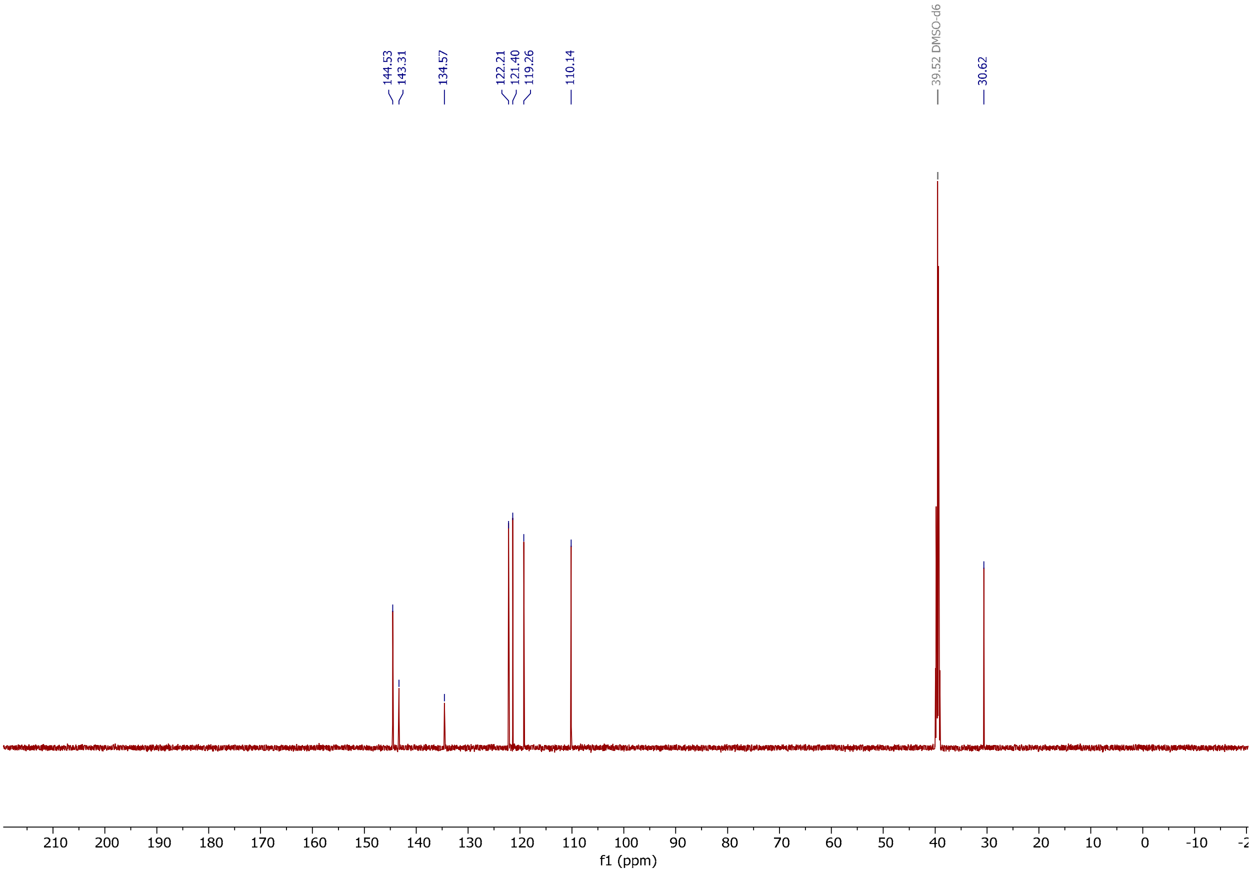
144.53
143.31
134.57
122.21
121.40
119.26
110.14
39.52 DMSO-d6
30.62
f1 (ppm)

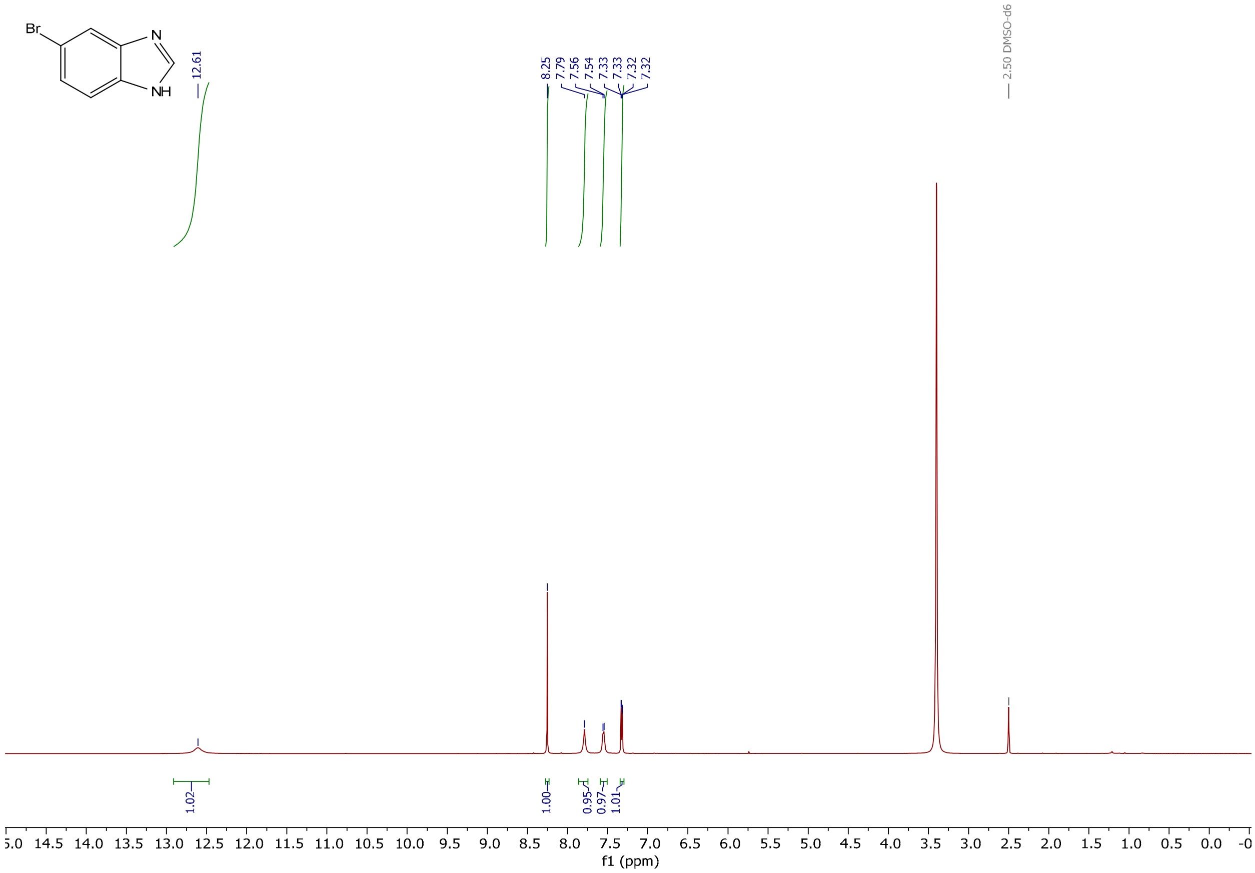
Br
N
NH
12.61
8.25
7.79
7.56
7.54
7.33
7.33
7.32
7.32
2.50 DMSO-d6
1.02
1.00
0.95
0.97
1.01
15.0 14.5 14.0 13.5 13.0 12.5 12.0 11.5 11.0 10.5 10.0 9.5 9.0 8.5 8.0 7.5 7.0 6.5 6.0 5.5 5.0 4.5 4.0 3.5 3.0 2.5 2.0 1.5 1.0 0.5 0.0
f1 (ppm)

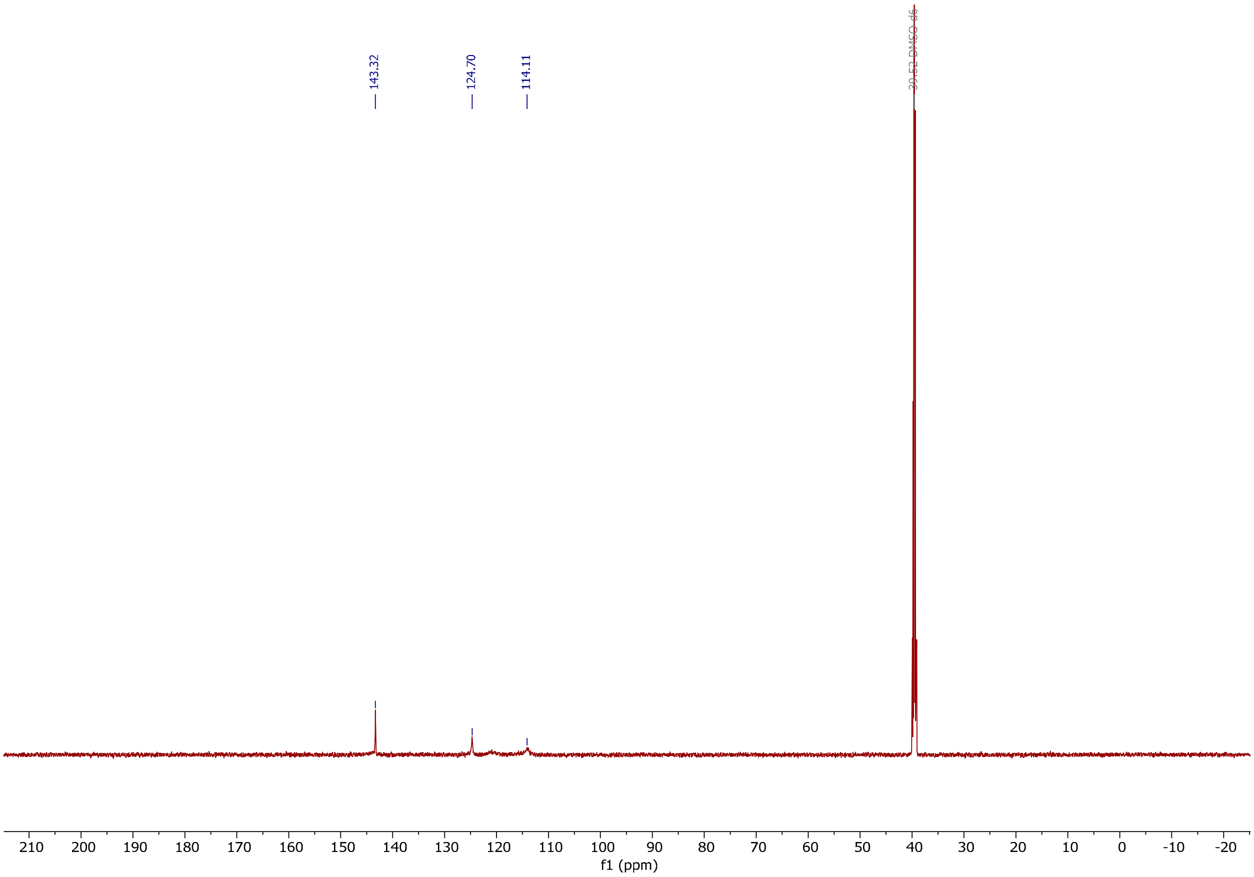
143.32
124.70
114.11
39.52 DMSO-d6
210 200 190 180 170 160 150 140 130 120 110 100 90 80 70 60 50 40 30 20 10 0 -10 -20
f1 (ppm)

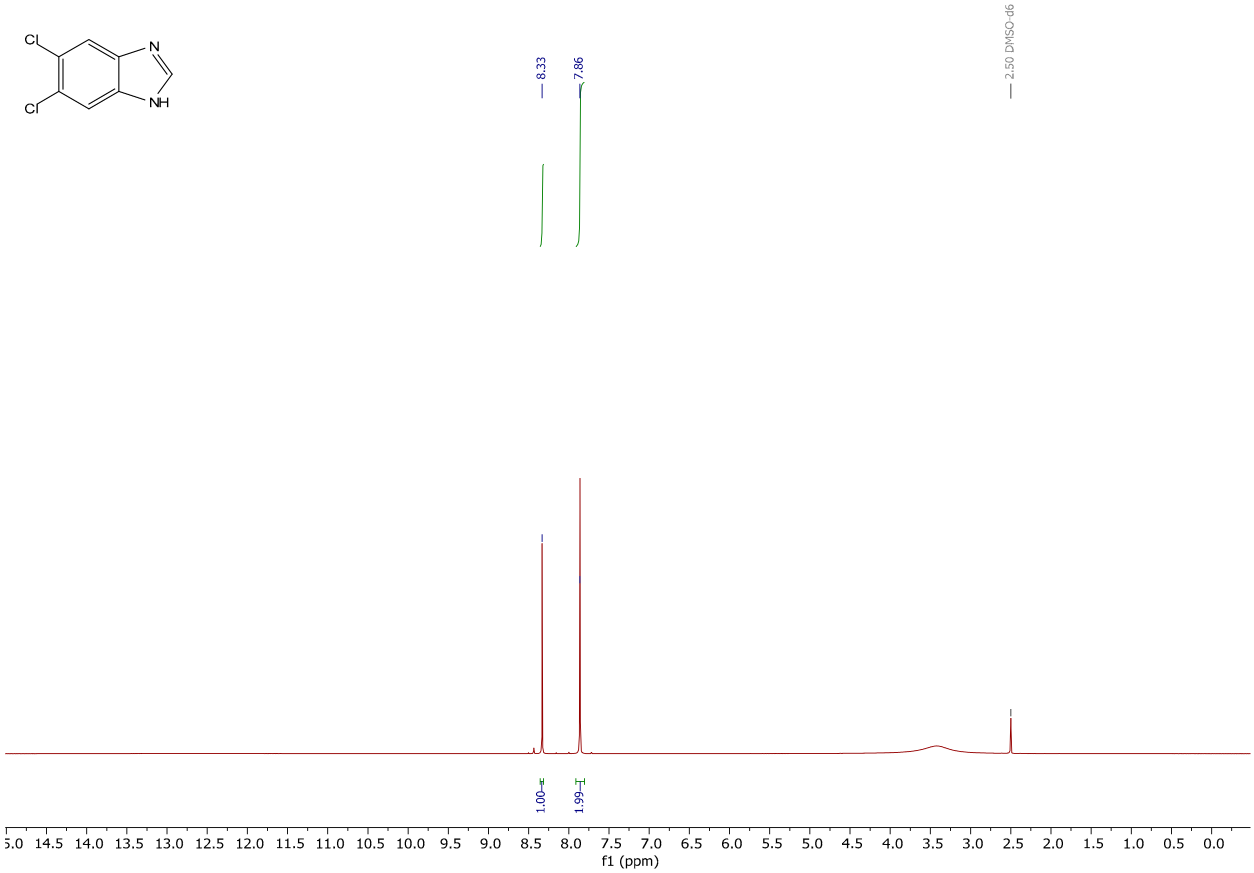
Cl
Cl
N
NH
8.33
7.86
2.50 DMSO-d6
1.00
1.99
5.0 14.5 14.0 13.5 13.0 12.5 12.0 11.5 11.0 10.5 10.0 9.5 9.0 8.5 8.0 7.5 7.0 6.5 6.0 5.5 5.0 4.5 4.0 3.5 3.0 2.5 2.0 1.5 1.0 0.5 0.0
f1 (ppm)

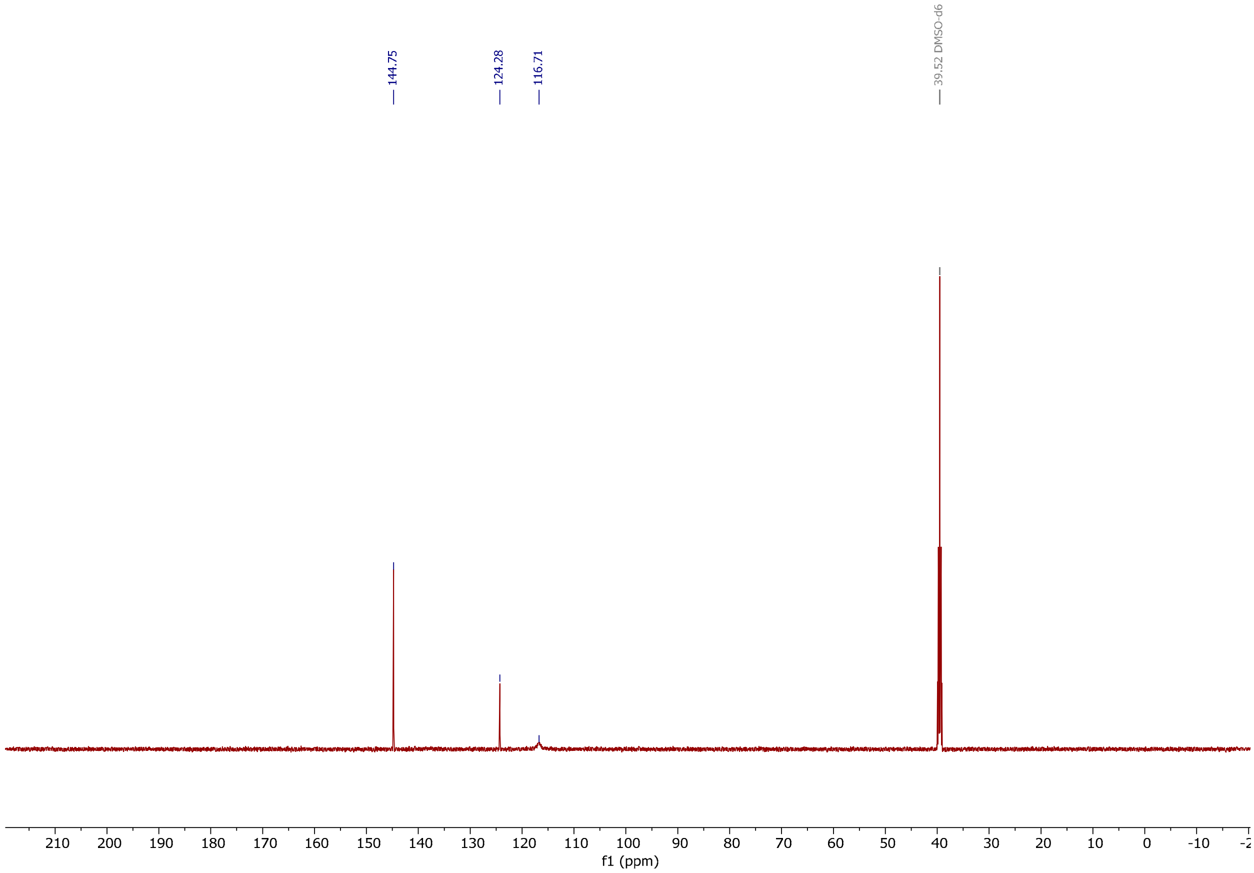
144.75
124.28
116.71
39.52 DMSO-d6
210 200 190 180 170 160 150 140 130 120 110 100 90 80 70 60 50 40 30 20 10 0 -10
f1 (ppm)

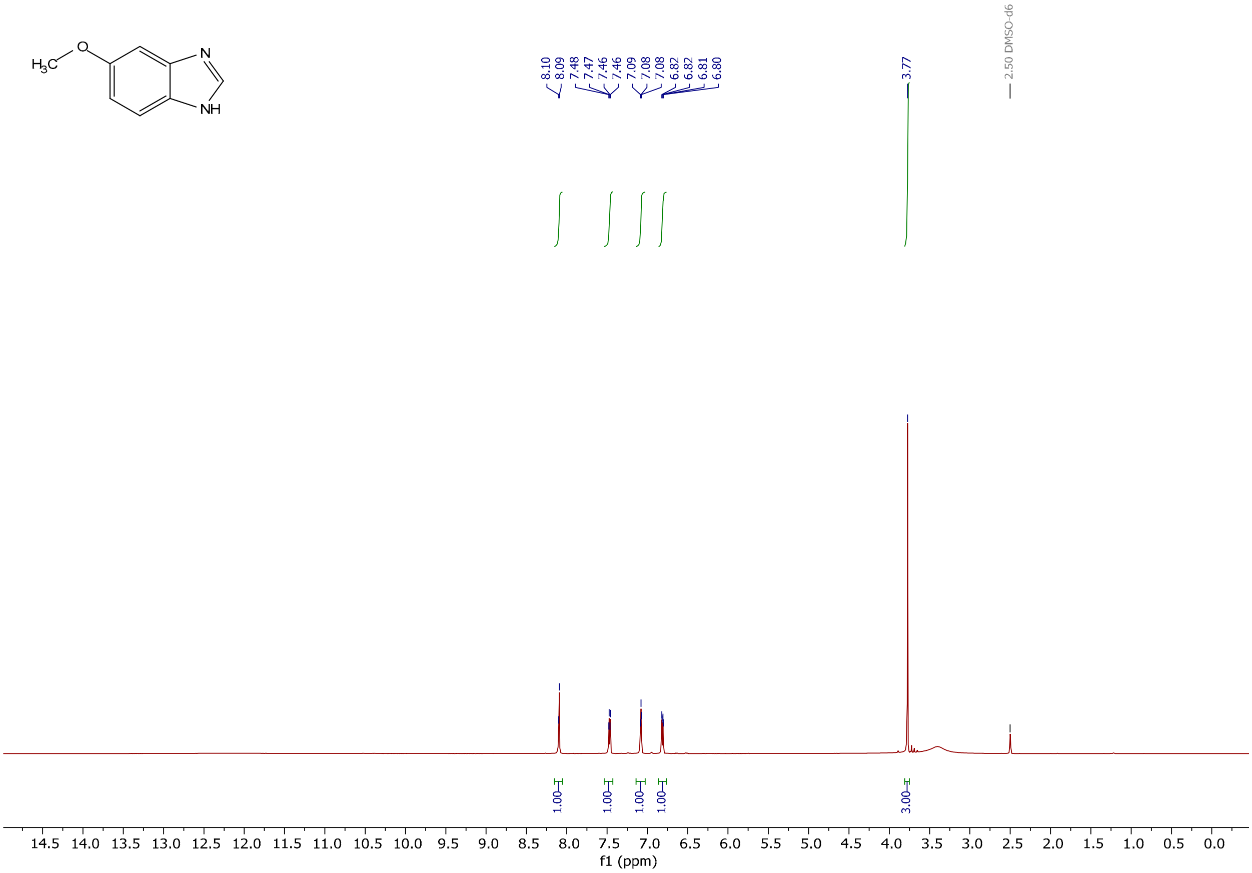
H3C O N NH
8.10
8.09
7.48
7.47
7.46
7.46
7.09
7.08
7.08
6.82
6.82
6.81
6.80
3.77
2.50 DMSO-d6
1.00
1.00
1.00
1.00
3.00
14.5 14.0 13.5 13.0 12.5 12.0 11.5 11.0 10.5 10.0 9.5 9.0 8.5 8.0 7.5 7.0 6.5 6.0 5.5 5.0 4.5 4.0 3.5 3.0 2.5 2.0 1.5 1.0 0.5 0.0
f1 (ppm)

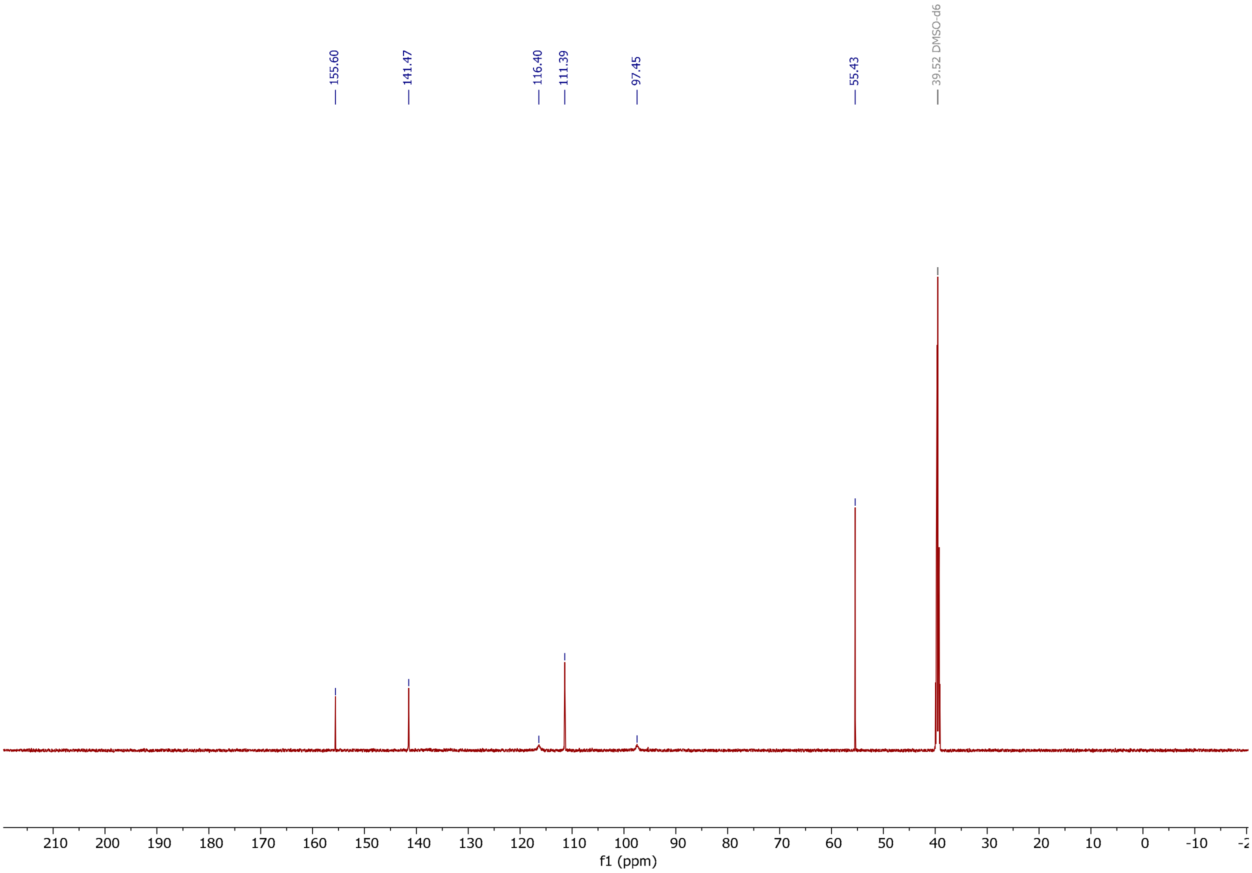
155.60
141.47
116.40
111.39
97.45
55.43
39.52 DMSO-d6
210 200 190 180 170 160 150 140 130 120 110 100 90 80 70 60 50 40 30 20 10 0 -10
f1 (ppm)

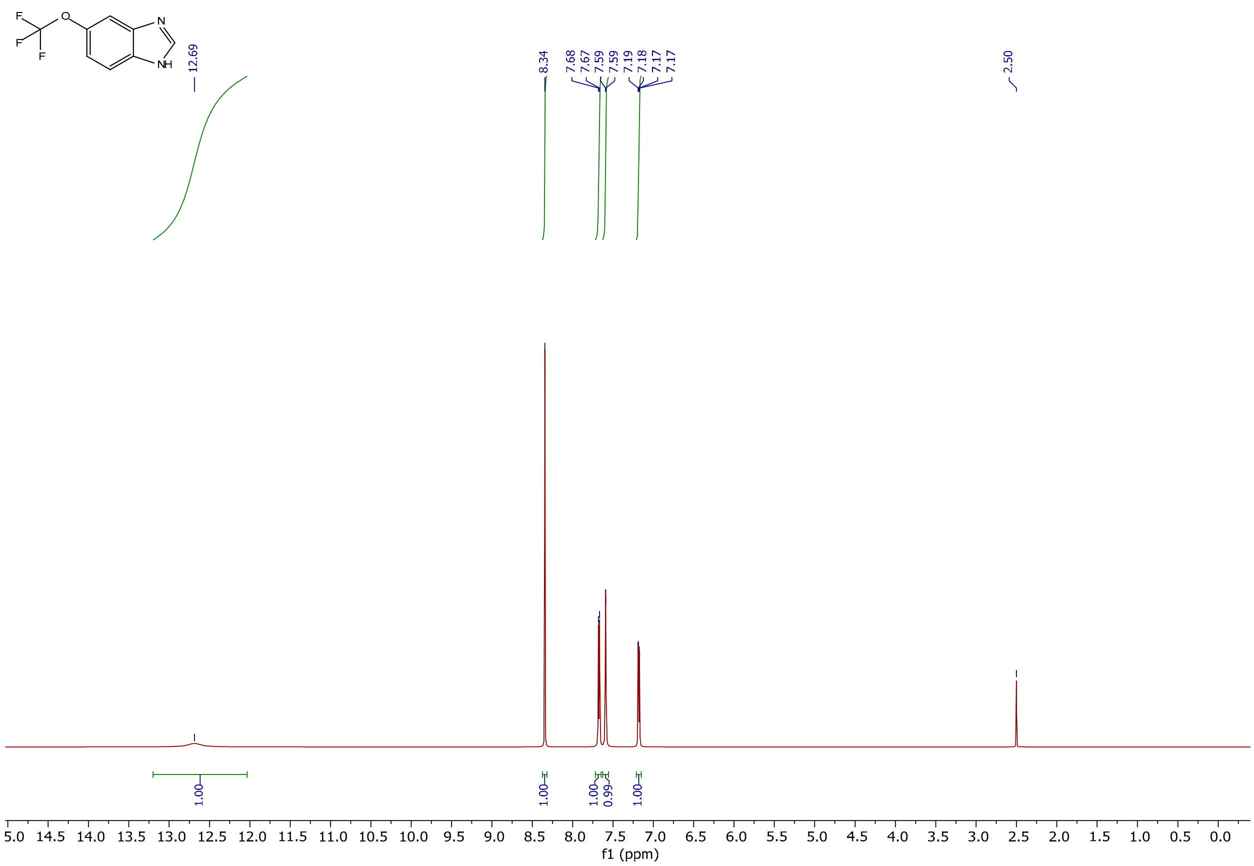
F
F
F
O
N
NH
12.69
8.34
7.68
7.67
7.59
7.59
7.19
7.18
7.17
7.17
2.50
1.00
1.00
1.00
0.99
1.00
f1 (ppm)

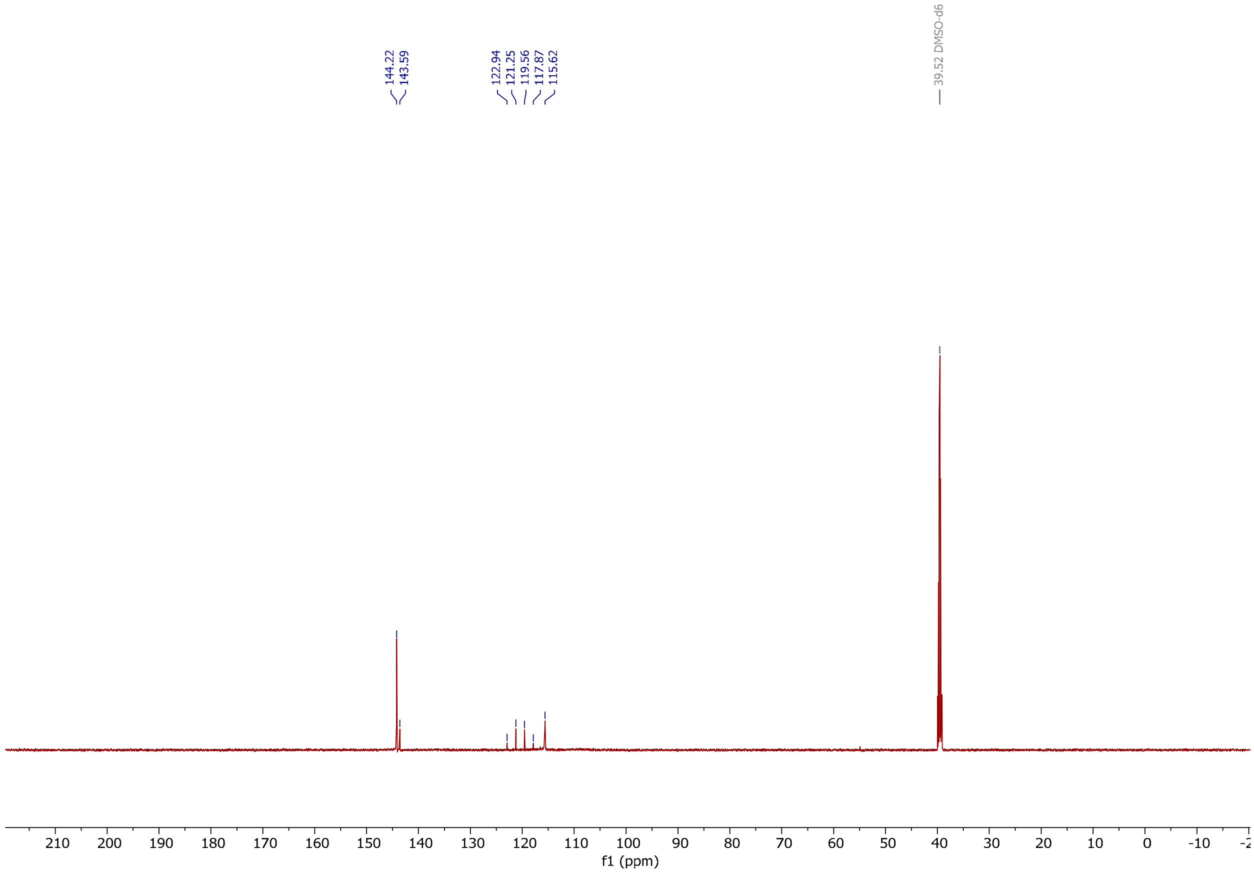
144.22
143.59
122.94
121.25
119.56
117.87
115.62
39.52 DMSO-d6
f1 (ppm)

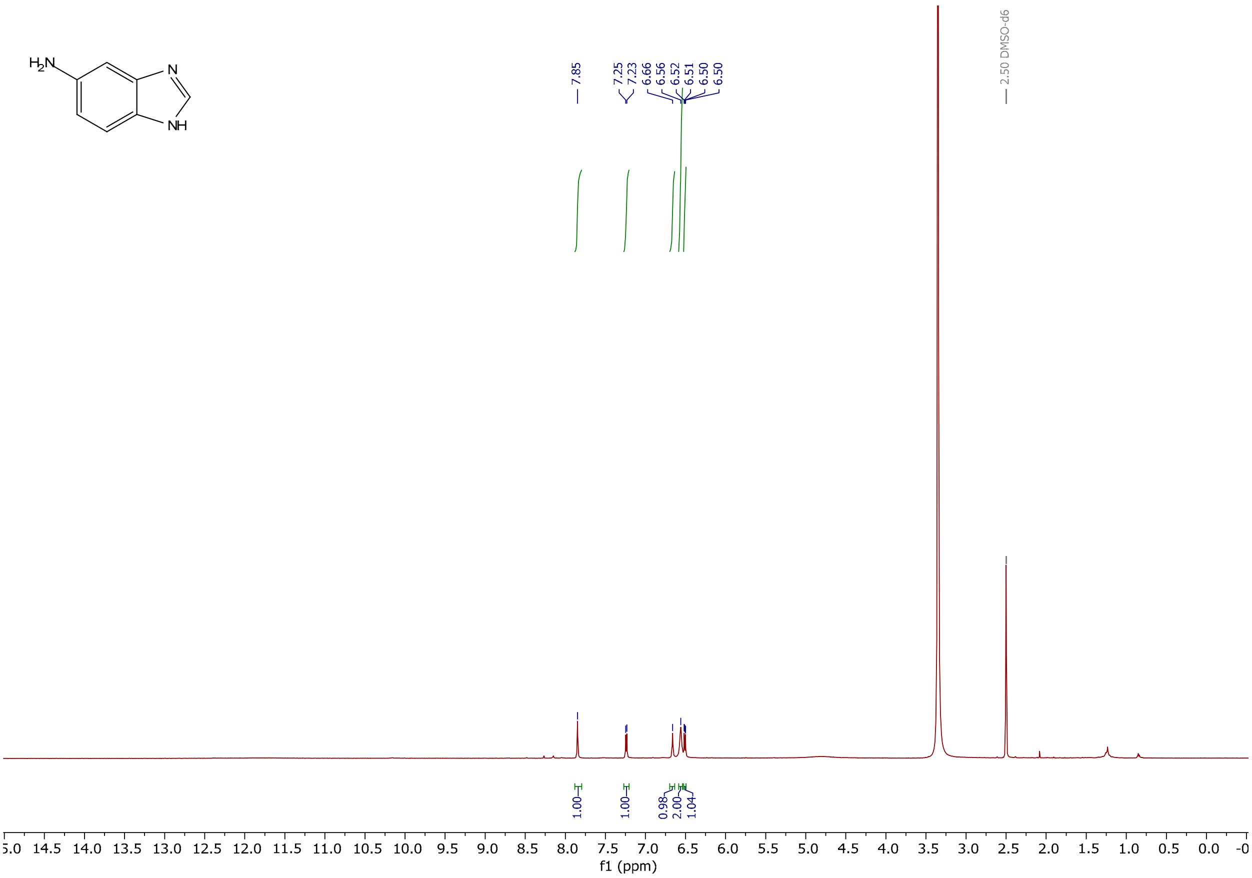
H2N
N
NH
7.85
7.25
7.23
6.66
6.56
6.52
6.51
6.50
6.50
2.50 DMSO-d6
1.00
1.00
0.98
2.00
1.04
f1 (ppm)

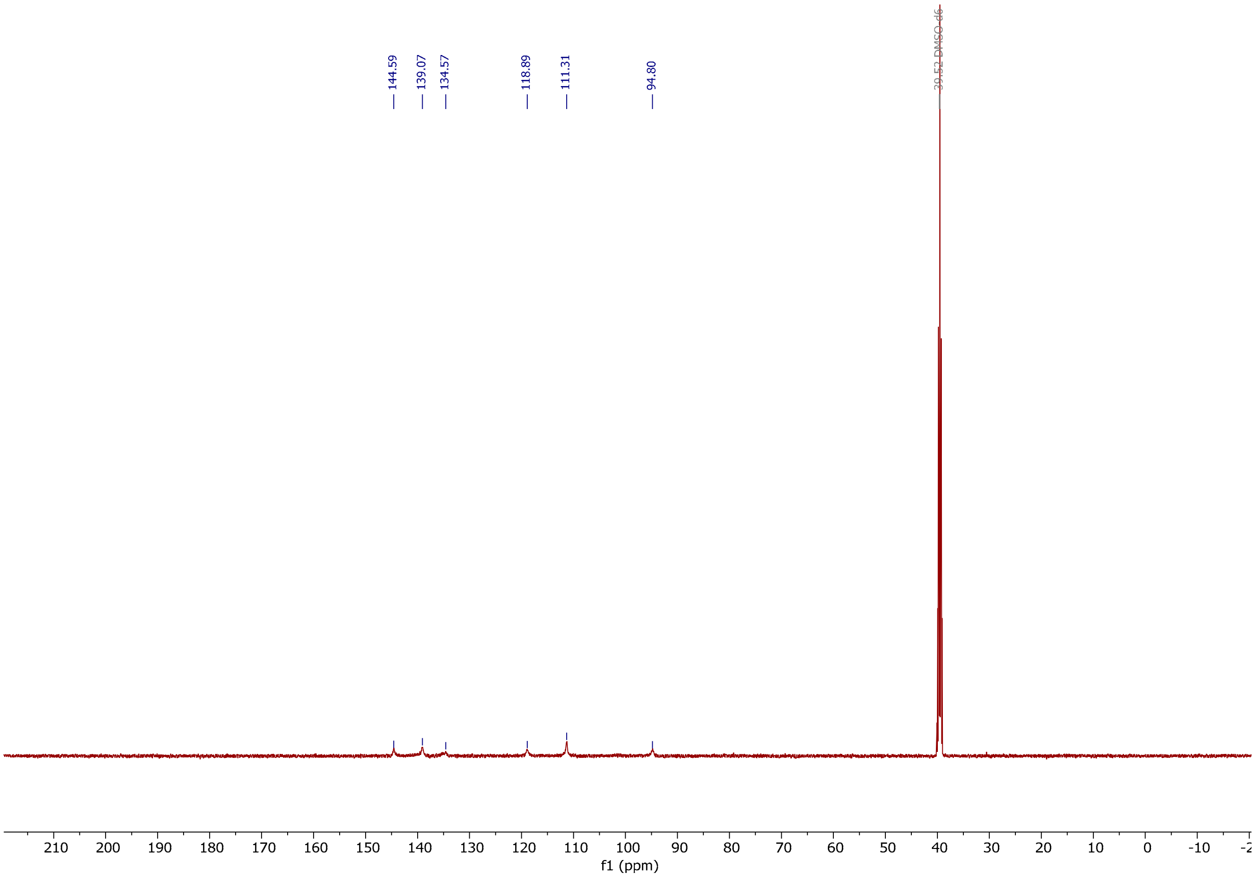
144.59
139.07
134.57
118.89
111.31
94.80
39.52 DMSO-d6
f1 (ppm)

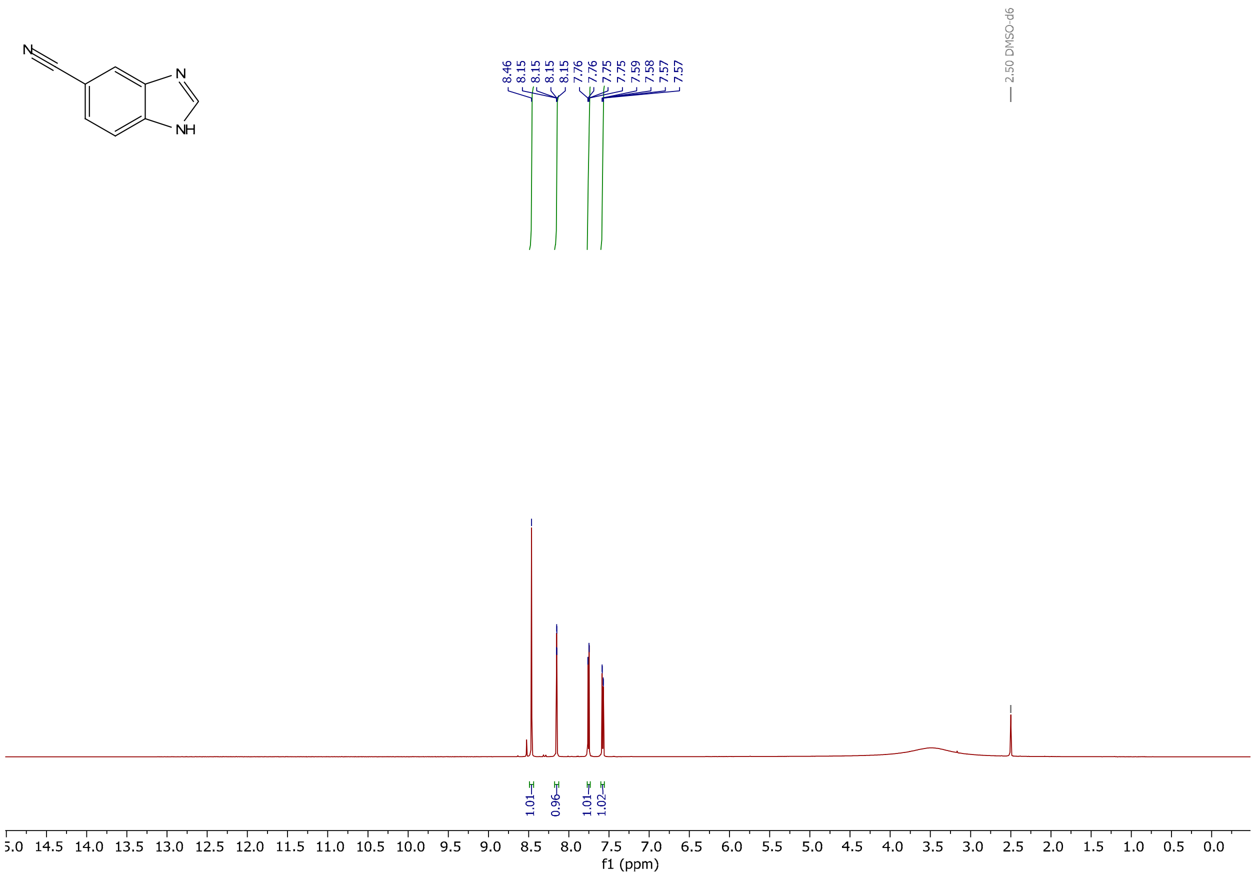
N
NH
8.46
8.15
8.15
8.15
8.15
7.76
7.76
7.75
7.75
7.59
7.58
7.57
7.57
2.50 DMSO-d6
1.01
0.96
1.01
1.02
f1 (ppm)

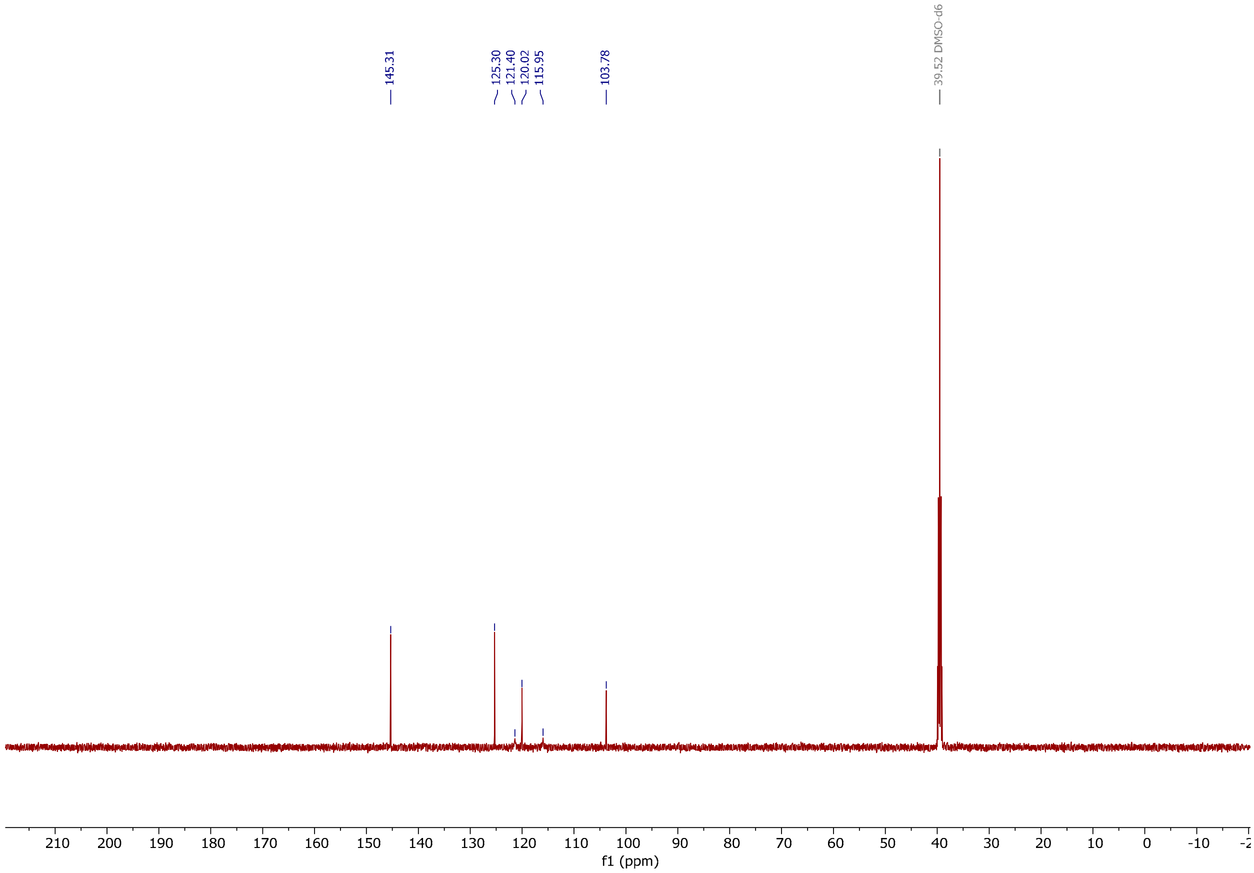
145.31
125.30
121.40
120.02
115.95
103.78
39.52 DMSO-d6
f1 (ppm)

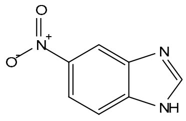
O
N+
O-
N
NH

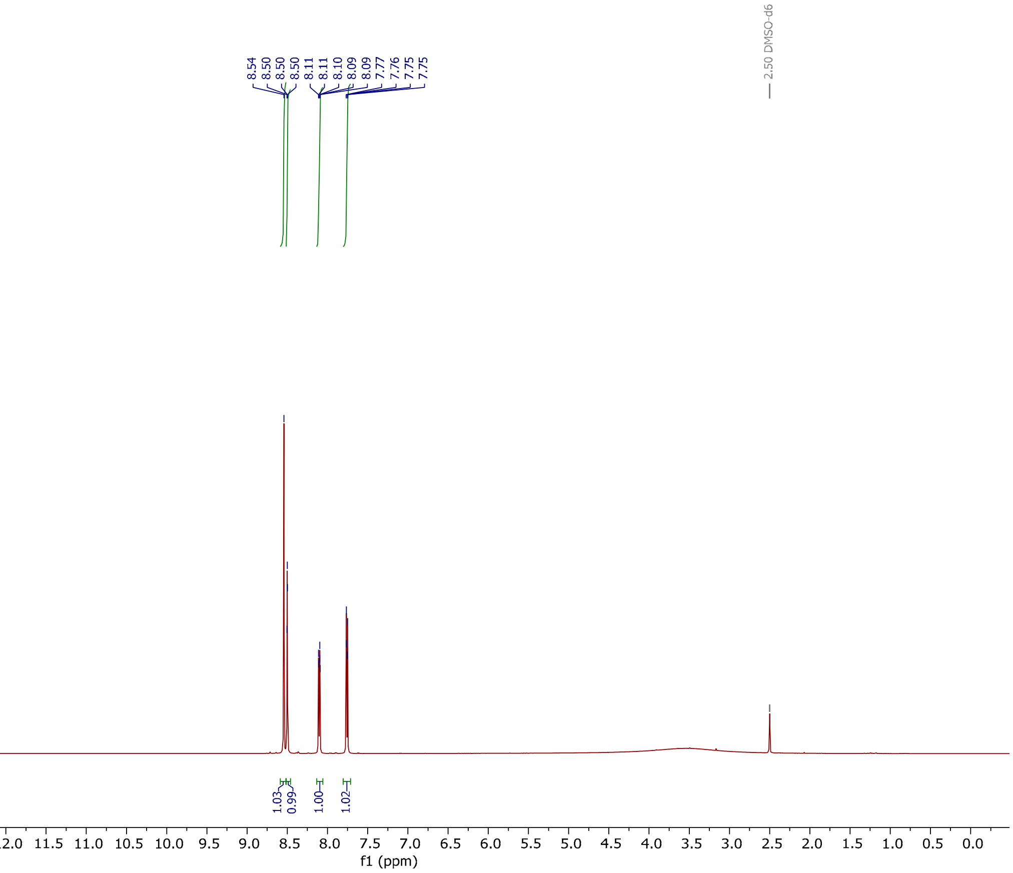
8.54
8.50
8.50
8.50
8.11
8.11
8.10
8.09
7.77
7.76
7.75
7.75
2.50 DMSO-d6
1.03
0.99
1.00
1.02
f1 (ppm)

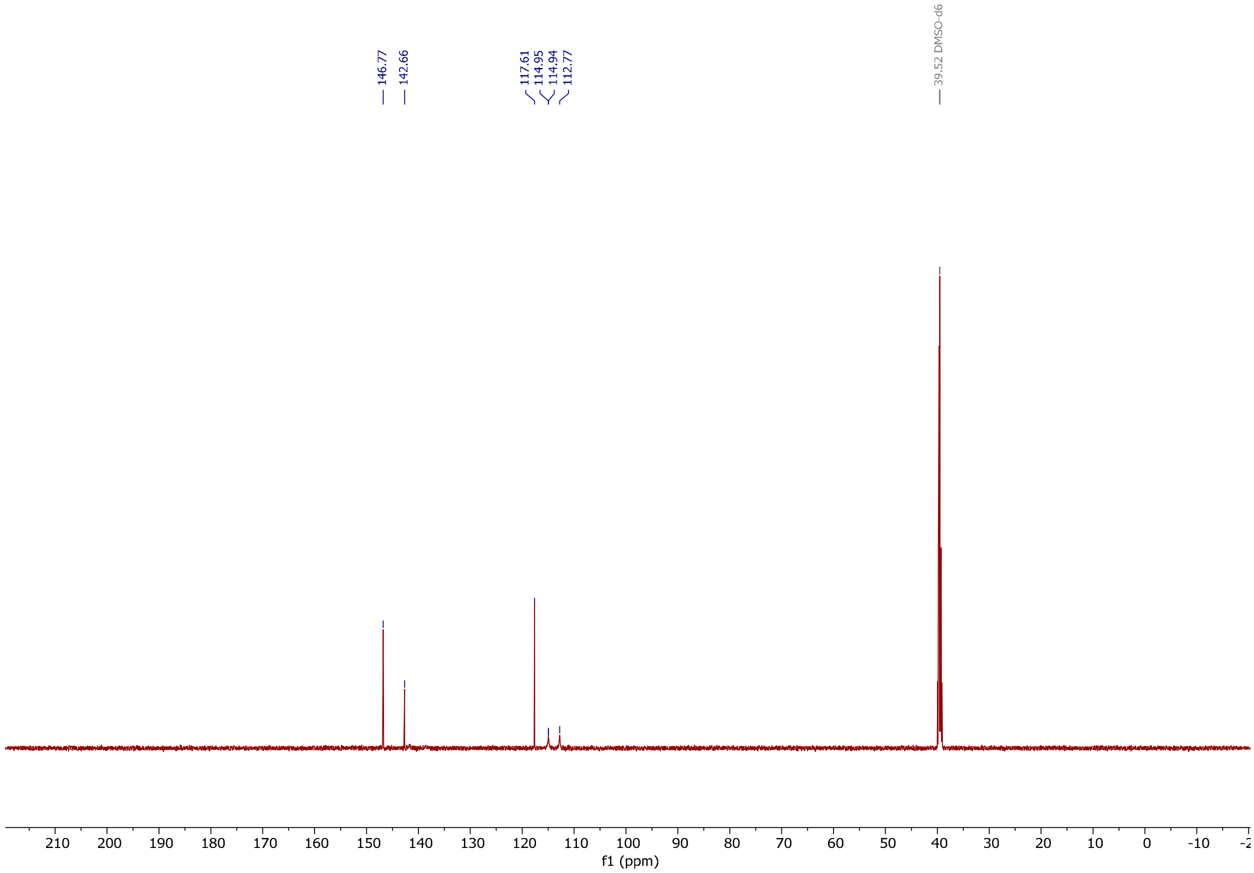
146.77
142.66
117.61
114.95
114.94
112.77
39.52 DMSO-d6
f1 (ppm)

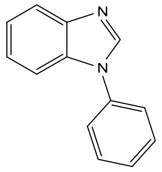
N
N

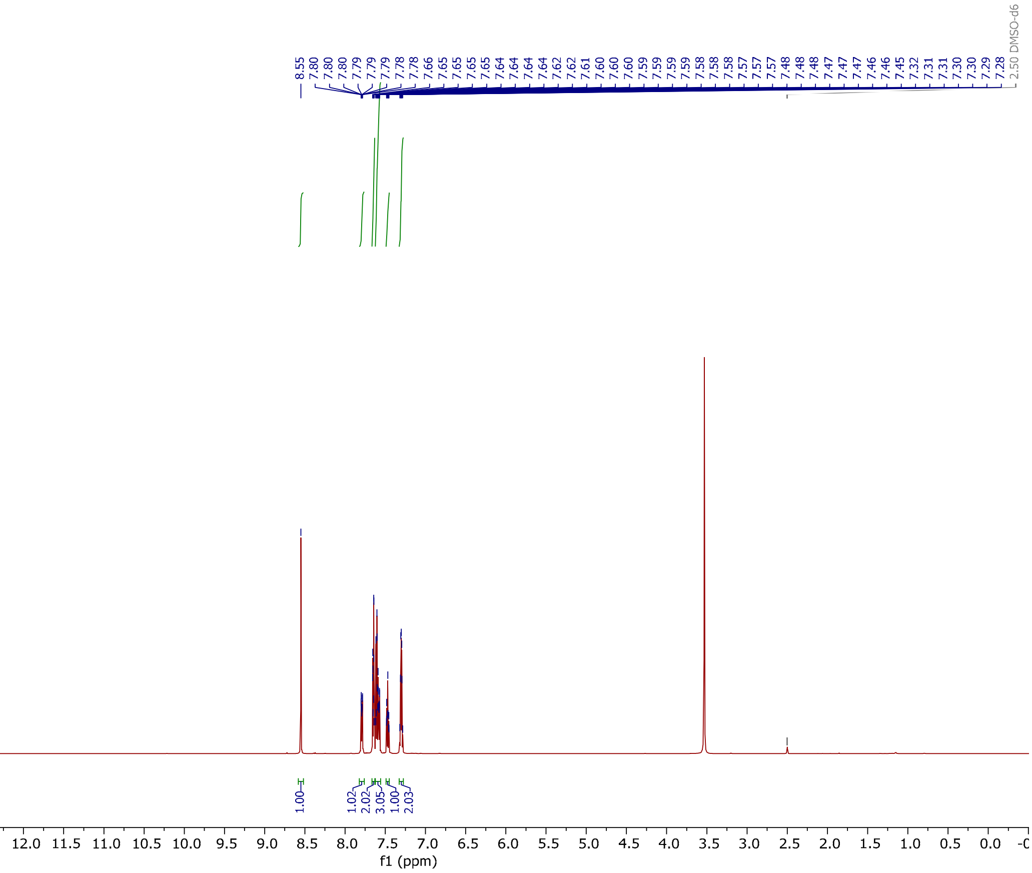
8.55
7.80
7.80
7.79
7.79
7.79
7.78
7.78
7.66
7.65
7.65
7.65
7.64
7.64
7.64
7.62
7.62
7.61
7.60
7.60
7.60
7.59
7.59
7.59
7.59
7.58
7.58
7.58
7.57
7.57
7.57
7.48
7.48
7.48
7.47
7.47
7.46
7.46
7.45
7.32
7.31
7.30
7.30
7.29
7.28
2.50 DMSO-d6
1.00
1.02
2.02
3.05
1.00
2.03
14.5 14.0 13.5 13.0 12.5 12.0 11.5 11.0 10.5 10.0 9.5 9.0 8.5 8.0 7.5 7.0 6.5 6.0 5.5 5.0 4.5 4.0 3.5 3.0 2.5 2.0 1.5 1.0 0.5 0.0
f1 (ppm)

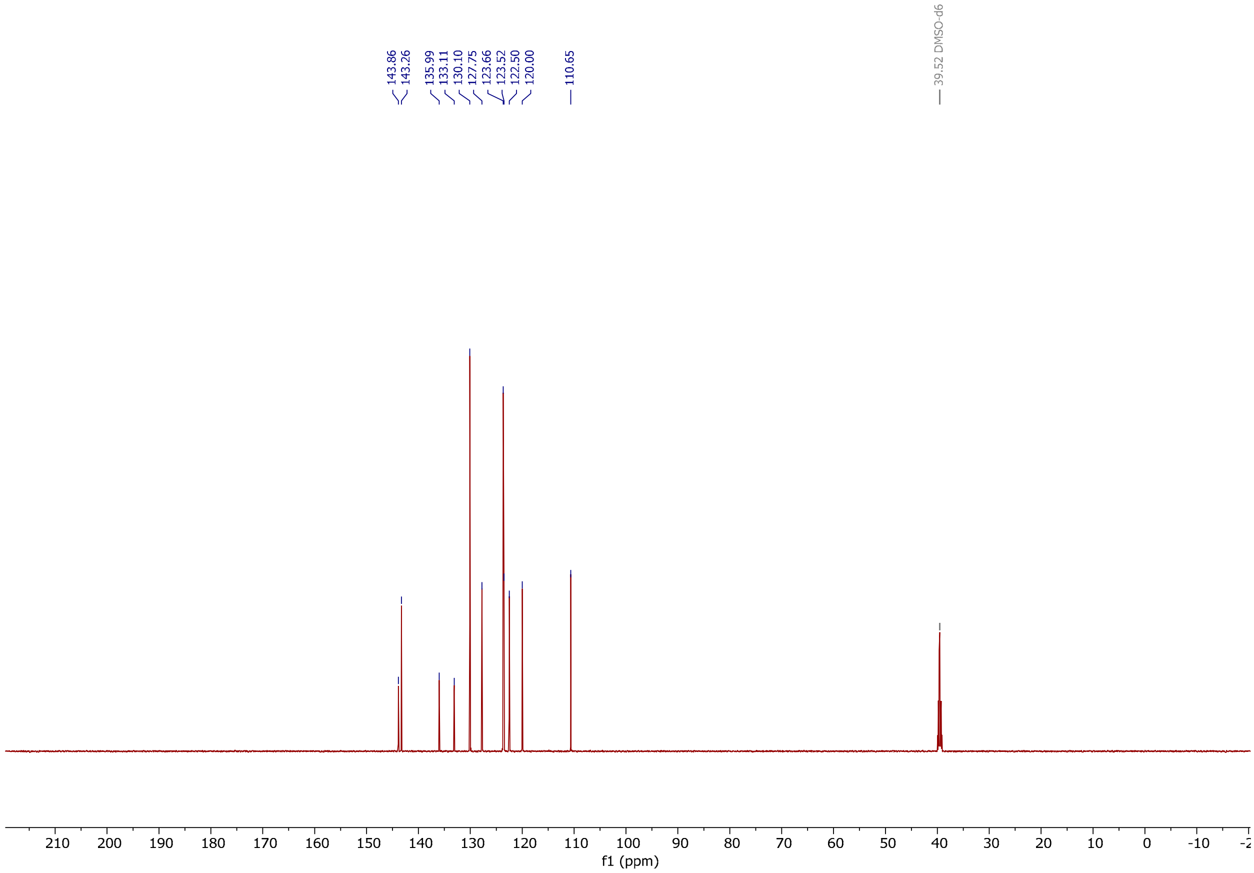
143.86
143.26
135.99
133.11
130.10
127.75
123.66
123.52
122.50
120.00
110.65
39.52 DMSO-d6
210 200 190 180 170 160 150 140 130 120 110 100 90 80 70 60 50 40 30 20 10 0 -10
f1 (ppm)

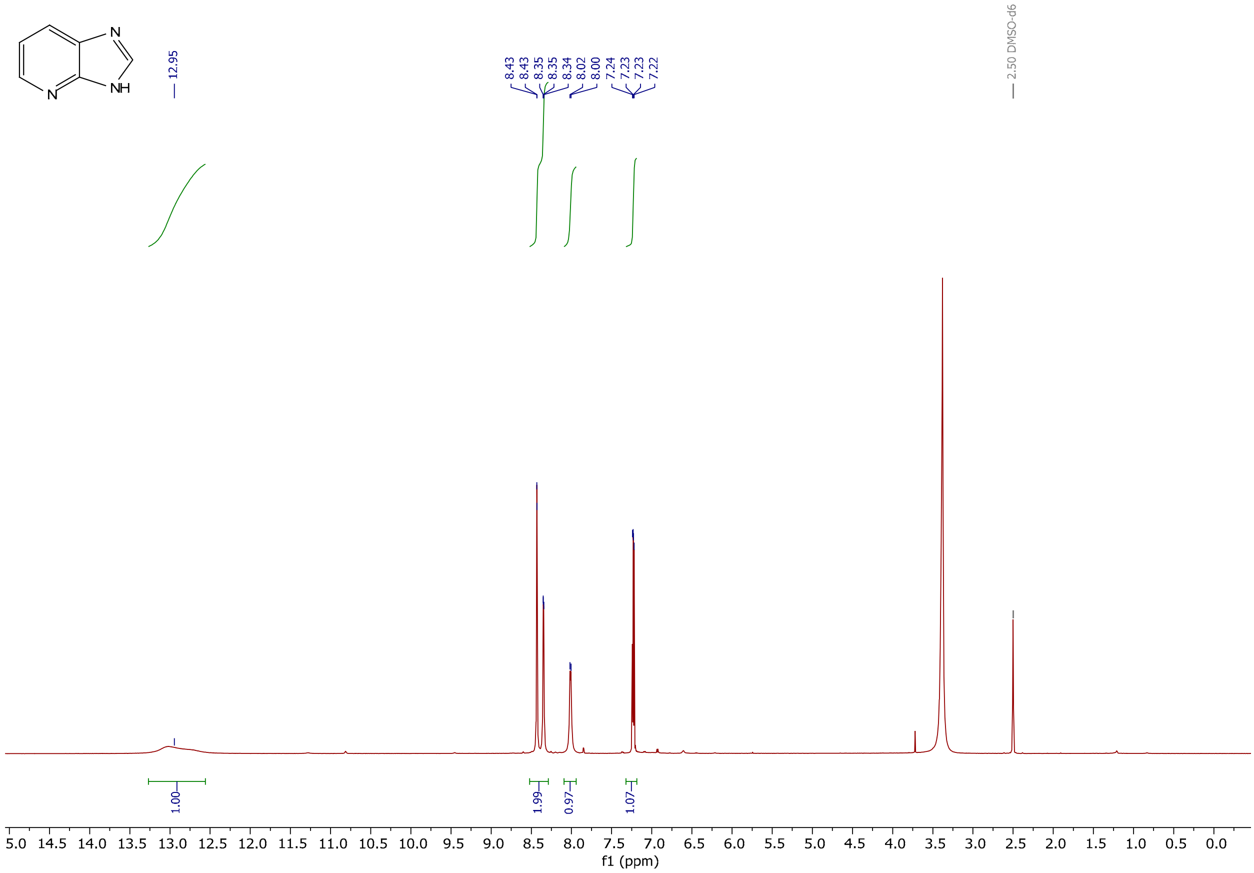
N
NH
N
12.95
8.43
8.43
8.35
8.35
8.34
8.02
8.00
7.24
7.23
7.23
7.22
2.50 DMSO-d6
1.00
1.99
0.97
1.07
f1 (ppm)

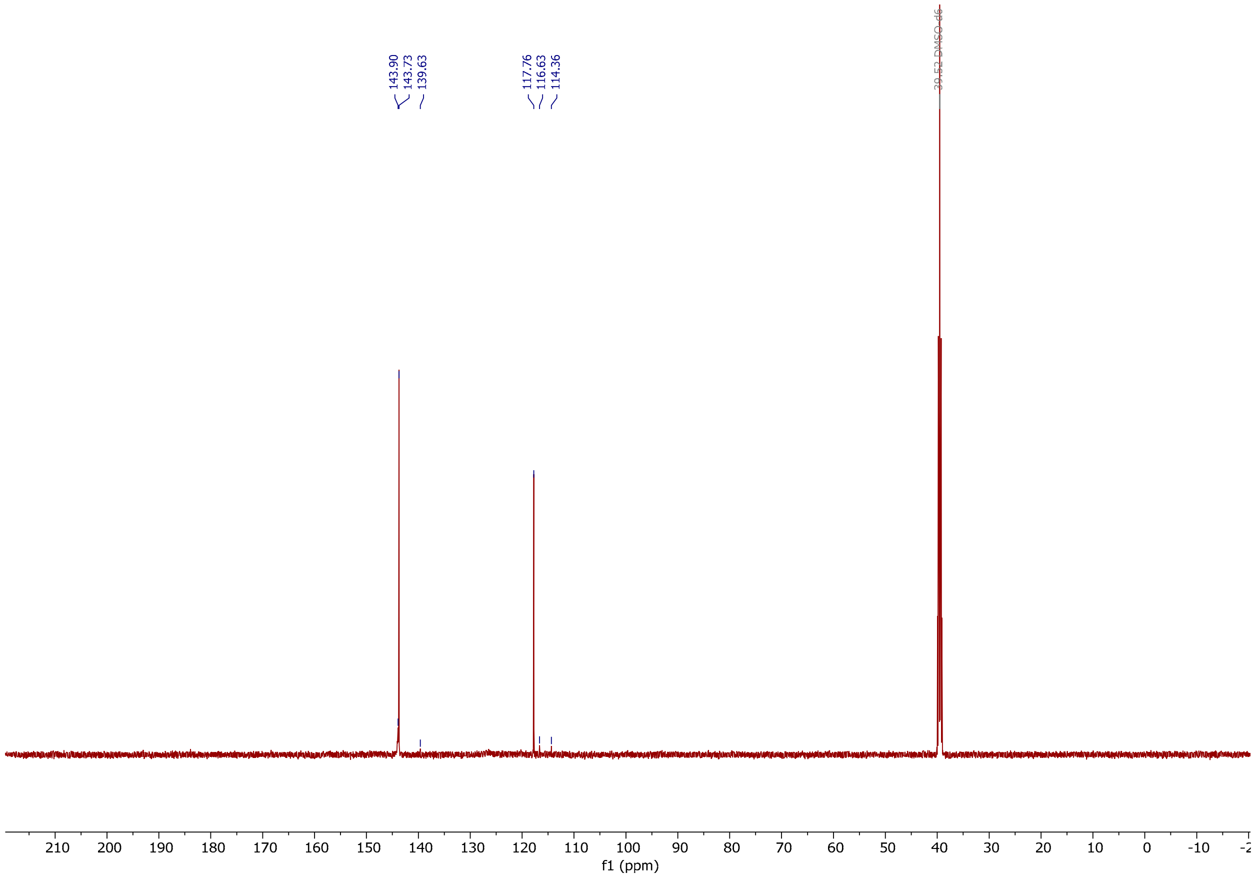
143.90
143.73
139.63
117.76
116.63
114.36
39.52 DMSO-d6
f1 (ppm)

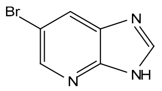
Br
N
NH
N

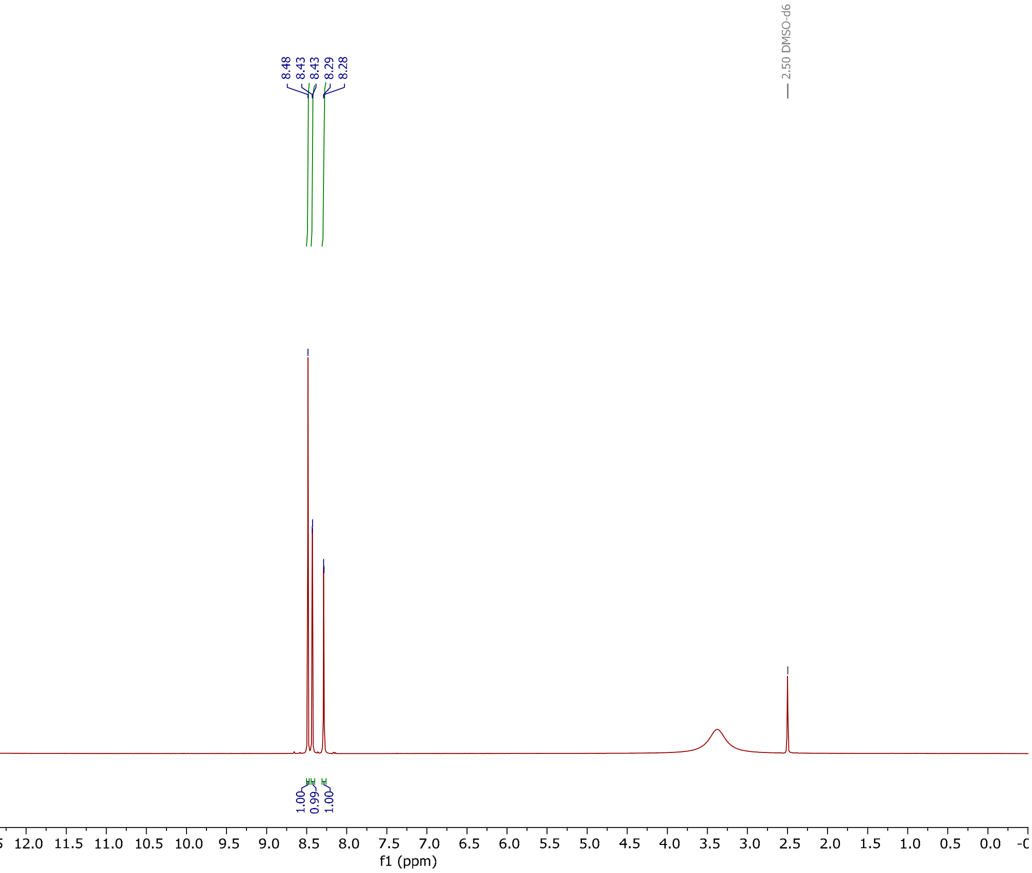
8.48
8.43
8.43
8.29
8.28
2.50 DMSO-d6
1.00
0.99
1.00
f1 (ppm)

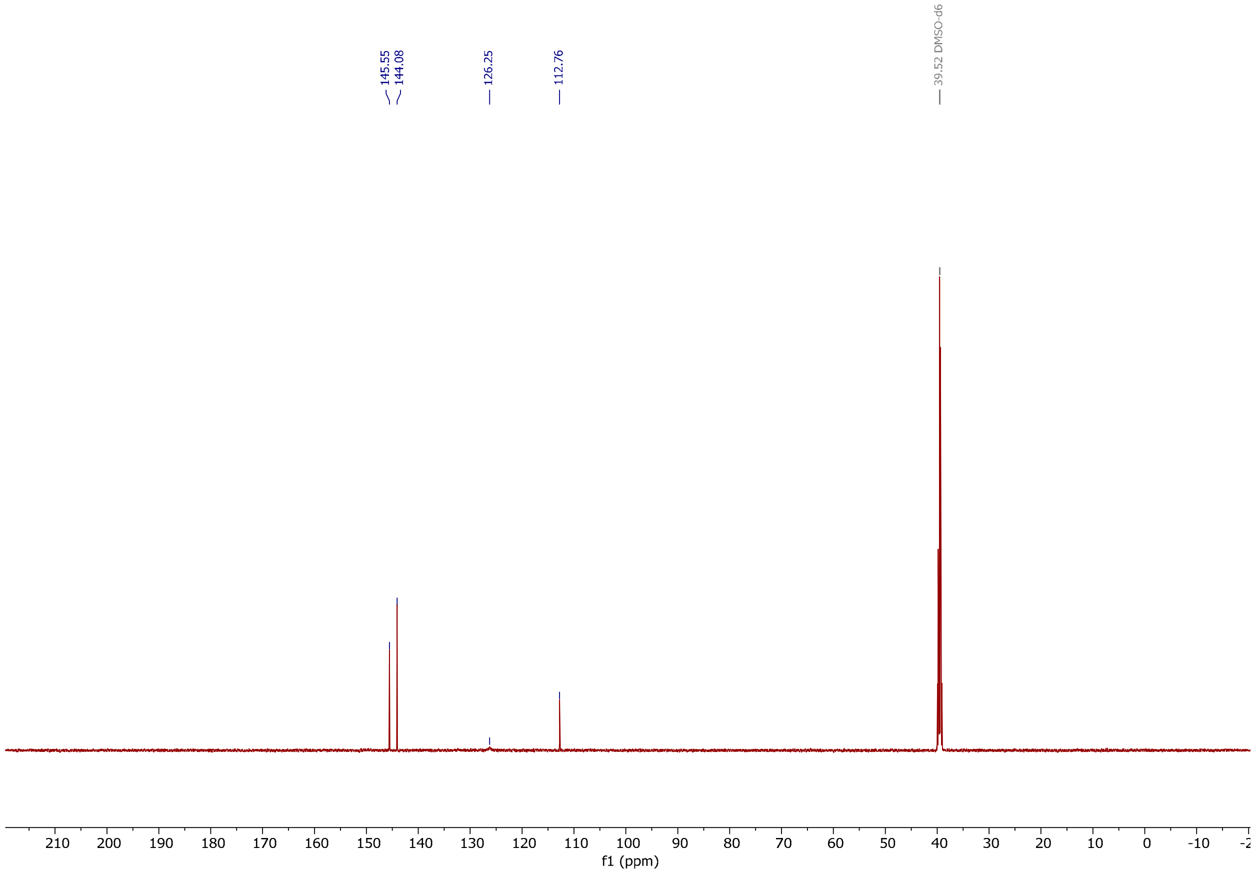
145.55
144.08
126.25
112.76
39.52 DMSO-d6
f1 (ppm)

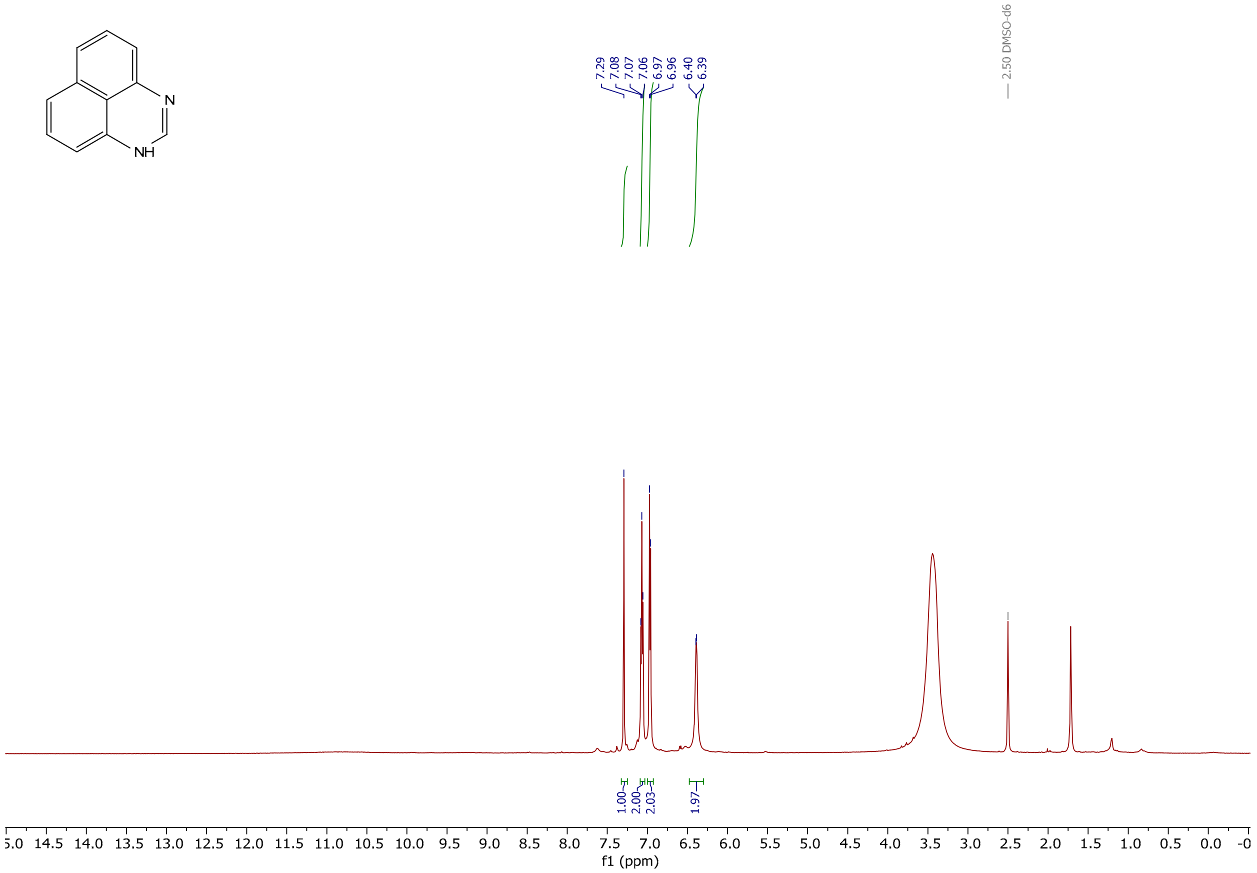
N
NH
7.29
7.08
7.07
7.06
6.97
6.96
6.40
6.39
2.50 DMSO-d6
1.00
2.00
2.03
1.97
f1 (ppm)

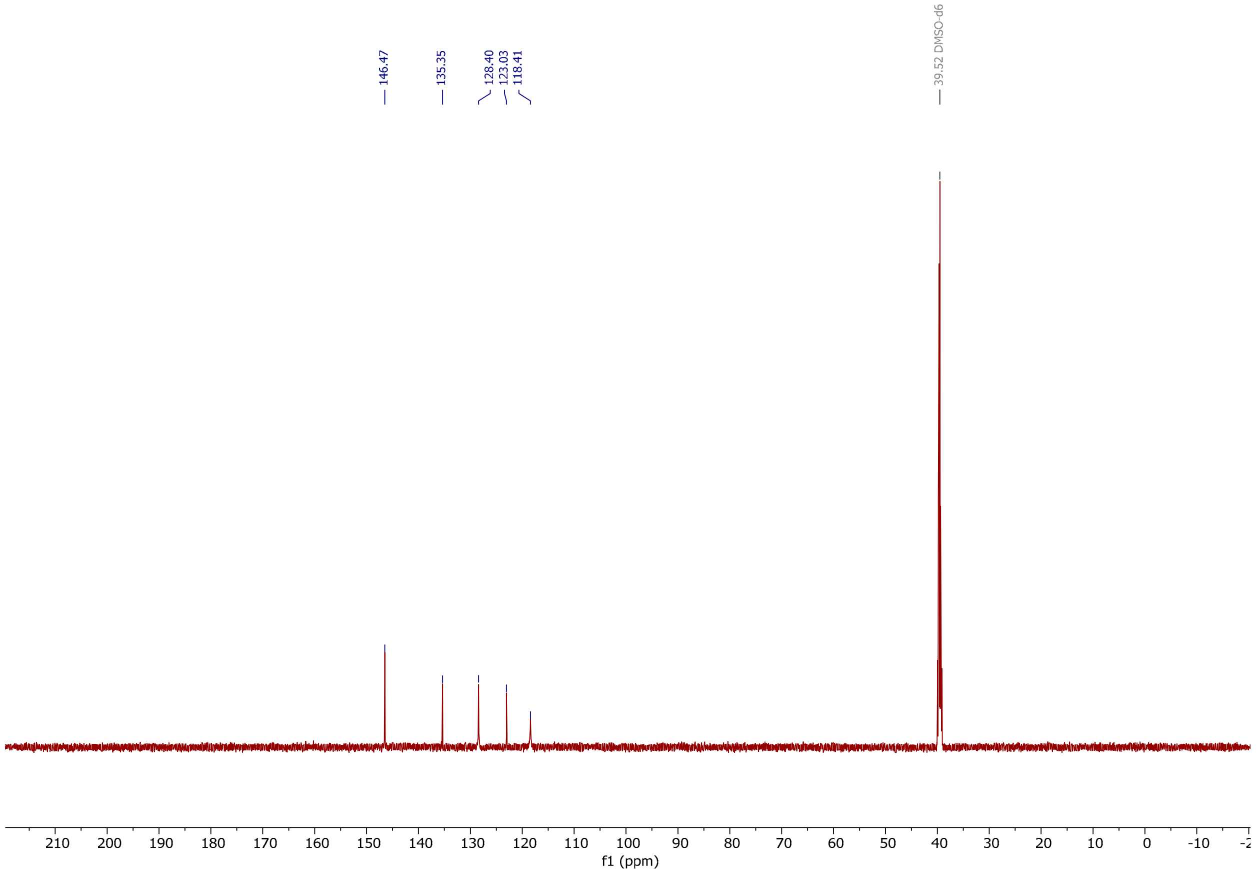
146.47
135.35
128.40
123.03
118.41
39.52 DMSO-d6
f1 (ppm)

5. References

107. Jiang, S.; Dong, X.; Qiu, Y.; Chen, D.; Wu, X.; Jiang, S. A new ligand for copper-catalyzed amination of aryl halides to primary(hetero)aryl amines. *Tetrahedron Lett.* **2020**, *61*, 151683, doi:https://doi.org/10.1016/j.tetlet.2020.151683.

108. Panja, S.; kundu, D.; Ahammed, S.; Ranu, B.C. Highly chemoselective reduction of azides to amines by Fe(0) nanoparticles in water at room temperature. *Tetrahedron Lett.* **2017**, *58*, 3457-3460, doi:https://doi.org/10.1016/j.tetlet.2017.07.076.

109. 3Liao, B.-S.; Liu, S.-T. Diamination of Phenylene Dihalides Catalyzed by a Dicopper Complex. *J. Org. Chem.* **2012**, *77*, 6653-6656, doi:10.1021/jo301244p.

110. Mo, C.; Zhang, Z.; Guise, C.P.; Li, X.; Luo, J.; Tu, Z.; Xu, Y.; Patterson, A.V.; Smaill, J.B.; Ren, X.; et al. 2-Aminopyrimidine Derivatives as New Selective Fibroblast Growth Factor Receptor 4 (FGFR4) Inhibitors. *ACS Med. Chem. Lett.* **2017**, *8*, 543-548, doi:10.1021/acsmedchemlett.7b00091.

111. Sorribes, I.; Liu, L.; Corma, A. Nanolayered Co–Mo–S Catalysts for the Chemoselective Hydrogenation of Nitroarenes. *ACS Catal.* **2017**, *7*, 2698-2708, doi:10.1021/acscatal.7b00170.

112. Romero, A.H.; Cerecetto, H. A Common, Facile and Eco-Friendly Method for the Reduction of Nitroarenes, Selective Reduction of Poly-Nitroarenes and Deoxygenation of N-Oxide Containing Heteroarenes Using Elemental Sulfur. *Eur. J. Org. Chem.* **2020**, *2020*, 1853-1865, doi:https://doi.org/10.1002/ejoc.202000064.

113. Zhang, Y.-C.; Shen, Q.; Zhu, M.-W.; Wang, J.; Du, Y.; Wu, J.; Li, J.-X. Modified Quinoxaline-Fused Oleanolic Acid Derivatives as Inhibitors of Osteoclastogenesis and Potential Agent in Anti-Osteoporosis. *ChemistrySelect* **2020**, *5*, 1526-1533, doi:https://doi.org/10.1002/slct.201904521.

114. Ji, A.; Ren, W.; Ai, H.-w. A highly efficient oxidative condensation reaction for selective protein conjugation. *Chem. Comm.* **2014**, *50*, 7469-7472, doi:10.1039/C4CC01551G.

115. Chaudhary, P.; Gupta, S.; Muniyappan, N.; Sabiah, S.; Kandasamy, J. Regioselective Nitration of N-Alkyl Anilines using tert-Butyl Nitrite under Mild Condition. *J. Org. Chem.* **2019**, *84*, 104-119, doi:10.1021/acs.joc.8b02377.

116. Penieres-Carrillo, J.-G.; Ríos-Guerra, H.; Pérez-Flores, J.; Rodríguez-Molina, B.; Torres-Reyes, Á.; Barrera-Téllez, F.; González-Carrillo, J.; Moreno-González, L.; Martínez-Zaldívar, A.; Nolasco-Fidencio, J.-J.; et al. Reevaluating the synthesis of 2,5-disubstituted-1H-benzimidazole derivatives by different green activation techniques and their biological activity as antifungal and antimicrobial inhibitor. *J .Heterocycl. Chem.* **2020**, *57*, 436-455, doi:https://doi.org/10.1002/jhet.3801.

117. Zhang, Z.; Sun, Q.; Xia, C.; Sun, W. CO2 as a C1 Source: B(C6F5)3-Catalyzed Cyclization of o-Phenylene-diamines To Construct Benzimidazoles in the Presence of Hydrosilane. *Org. Lett.* **2016**, *18*, 6316-6319, doi:10.1021/acs.orglett.6b03030.

118. Yu, B.; Zhang, H.; Zhao, Y.; Chen, S.; Xu, J.; Huang, C.; Liu, Z. Cyclization of o-phenylenediamines by CO2 in the presence of H2 for the synthesis of benzimidazoles. *Green Chem.* **2013**, *15*, 95-99, doi:10.1039/C2GC36517K.

119. Graham, T.H. Deprotection of N-benzylbenzimidazoles and N-benzylimidazoles with triethylsilane and Pd/C. *Tetrahedron Lett.* **2015**, *56*, 2688-2690, doi:https://doi.org/10.1016/j.tetlet.2015.03.127.

120. Zhu, K.; Hao, J.-H.; Zhang, C.-P.; Zhang, J.; Feng, Y.; Qin, H.-L. Diversified facile synthesis of benzimidazoles, quinazolin-4(3H)-ones and 1,4-benzodiazepine-2,5-diones via palladium-catalyzed

transfer hydrogenation/condensation cascade of nitro arenes under microwave irradiation. *RSC Adv.* **2015**, *5*, 11132-11135, doi:10.1039/C4RA15765F.

121. Xu, L.-L.; Zhu, J.-F.; Xu, X.-L.; Zhu, J.; Li, L.; Xi, M.-Y.; Jiang, Z.-Y.; Zhang, M.-Y.; Liu, F.; Lu, M.-c.; et al. Discovery and Modification of in Vivo Active Nrf2 Activators with 1,2,4-Oxadiazole Core: Hits Identification and Structure–Activity Relationship Study. *J. Med. Chem.* **2015**, *58*, 5419-5436, doi:10.1021/acs.jmedchem.5b00170.

122. Aziz, J.; Baladi, T.; Piguel, S. Direct Alkynylation of 3H-Imidazo[4,5-b]pyridines Using gem-Dibromoalkenes as Alkynes Source. *J. Org. Chem.* **2016**, *81*, 4122-4133, doi:10.1021/acs.joc.6b00406.

123. Gahlon, H.L.; Schweizer, W.B.; Sturla, S.J. Tolerance of Base Pair Size and Shape in Postlesion DNA Synthesis. *J. Am. Chem. Soc.* **2013**, *135*, 6384-6387, doi:10.1021/ja311434s.

124. Common Solvents Properties. Available online: https://macro.lsu.edu/howto/solvents/Dichloromethane.htm (accessed on 30 January 2023)
